# Safety and efficacy of whole-body chlorhexidine gluconate cleansing with or without emollient in hospitalised neonates (NeoCHG): a multicentre, randomised, open-label, factorial pilot trial

**DOI:** 10.1016/j.eclinm.2024.102463

**Published:** 2024-02-25

**Authors:** Neal Russell, Michelle N. Clements, Kazi Shammin Azmery, Adrie Bekker, Julia Bielicki, Angela Dramowski, Sally Ellis, Aaqilah Fataar, Mahbubul Hoque, Kristen LeBeau, Seamus O’Brien, Francesca Schiavone, Peter Skoutari, Mohammad Shahidul Islam, Samir K. Saha, Ann Sarah Walker, Andrew Whitelaw, Michael Sharland

**Affiliations:** a Centre for Neonatal and Paediatric Infection, St George’s University, London, United Kingdom; b MRC Clinical Trials Unit at UCL, London, United Kingdom; c Child Health Research Foundation (CHRF), Dhaka Shishu Hospital, Dhaka, Bangladesh; d Department of Paediatrics and Child Health, Faculty of Medicine and Health Sciences, Stellenbosch University, Cape Town, South Africa; e Global Antibiotic Research and Development Partnership (GARDP), Geneva, Switzerland; f Bangladesh Shishu Hospital and Institute, Dhaka, Bangladesh; g Child Health Research Foundation (CHRF), Bangladesh Shishu Hospital and Institute, Dhaka, Bangladesh; h Division of Medical Microbiology, Faculty of Medicine and Health Sciences, Stellenbosch University, Cape Town, South Africa

**Keywords:** Neonatal sepsis, Infection prevention, Antiseptic

## Abstract

**Background:**

Healthcare-associated infections account for substantial neonatal in-hospital mortality. Chlorhexidine gluconate (CHG) whole body skin application could reduce sepsis by lowering bacterial colonisation density, although safety and optimal application regimen is unclear. Emollients, including sunflower oil, may independently improve skin condition, thereby reducing sepsis. We aimed to inform which concentration and frequency of CHG, with or without emollient, would best balance safety and the surrogate marker of efficacy of reduction in bacterial colonisation, to be taken forward in a future pragmatic trial evaluating clinical outcomes of sepsis and mortality.

**Methods:**

In this multicentre, randomised, open-label, factorial pilot trial, neonates in two hospital sites (South Africa, Bangladesh) aged 1–6 days with gestational age ≥ 28 weeks and birthweight 1000–1999 g were randomly assigned in a factorial design stratified by site to three different concentrations of CHG (0.5%, 1%, and 2%), with or without emollient (sunflower oil) applied on working days vs alternate working days. A control arm received neither product. Caregivers were unblinded although laboratory staff were blinded to randomisation Co-primary outcomes were safety (change in neonatal skin condition score incorporating dryness, erythema, and skin breakdown) and efficacy in reducing bacterial colonisation density (change in total skin bacterial log_10_ CFU from randomisation to day-3 and day-8). The trial is registered at the ISRCTN registry, ISRCTN 69836999.

**Findings:**

Between Apr 12 2021 and Jan 18 2022, 208 infants were randomised and 198 were included in the final analysis. Skin condition scores were low with mean 0.1 (sd = 0.3; N = 208) at baseline, 0.1 (sd = 0.3; N = 199) at day 3 and 0.1 (sd = 0.3; N = 189) at day 8, with no evidence of differences between concentration (1% CHG vs 0.5% estimate = −0.3, 95% CI = (−1.2, 0.6), p = 0.55. 2% CHG vs 0.5% CHG estimate = 0.5 (−0.4, 1.4), p = 0.30), increasing frequency (estimate = −0.4; 95% CI = (−1.1, 0.4), p = 0.33), emollient (estimate = −0.5, (−1.2, 0.3), p = 0.23) or with control (estimate = −0.9, (−2.3, 0.4), p = 0.18). Mean log_10_ CFU was 4.9 (sd = 3.0; N = 208) at baseline, 6.3 (sd = 3.1; N = 198) at day 3 and 8.4 (sd = 2.6; N = 183) with no evidence of differences between concentration (1% CHG vs 0.5% estimate = −0.4; 95% CI = (−1.1, 0.23); p = 0.23. 2% CHG vs 0.5% CHG estimate = 0.0 (−0.6, 0.6), p = 0.96), with increasing frequency (estimate = −0.4; 95% CI = (−0.9, 0.2); p = 0.17), with emollient (estimate = 0.4, 95% CI = (−0.2, 0.9); p = 0.18) or with control (estimate = −0.2, 95% CI = (−1.3, 0.9); p = 0.73). By day-8, overall 158/183 (86%) of neonates were colonised with *Enterobacterales*, and 72/183 (39%) and 69/183 (9%) with *Klebsiella* spp resistant to third-generation cephalosporin and carbapenems, respectively. There were no CHG-related SAEs, emollient-related SAEs, grade 3 or 4 skin scores or grade 3 or 4 hypothermias.

**Interpretation:**

In this pilot trial of CHG with or without sunflower oil, no safety issues were identified, and further trials examining clinical outcomes are warranted. The relatively late start application of emollient, at a mean of 3.8 days of life, may have reduced the impact of the intervention although no subgroup effects were detected. There was no clear evidence in favour of a specific concentration of chlorhexidine, and there was rapid colonisation with Enterobacterales with frequent antimicrobial resistance, regardless of skin application regimen.

**Funding:**

The 10.13039/501100000265MRC Joint Applied 10.13039/100006090Global Health award, the Global Antibiotic Research and Development Partnership (GARDP), MRC Clinical Trials Unit core funding (10.13039/100014013UKRI) and 10.13039/501100004337St. George's, University of London.

**Research in context**

**Evidence before this study**

We systematically searched Medline and EMBASE for trials of chlorhexidine skin cleansing or bathing in hospitalised neonates (last search 1 May 2023), using MeSH and free text search terms for chlorhexidine/skin antisepsis and neonates combined with a high-sensitivity filter for randomised controlled trials. No language or date restrictions were applied. In addition, we searched the reference lists of relevant systematic reviews, including of reviews identified during the primary search. Identified trials largely focused on the use of chlorhexidine for community-based cord care or chlorhexidine bathing to prevent venous access-associated bloodstream infections. Three small LMIC single-centre trials (Nepal, India, South Africa) investigated the effectiveness of chlorhexidine on skin bacterial colonization among newborns in hospital. However, the first of these trials excluded infants admitted to neonatal intensive care and the second did not systematically apply the two trialled chlorhexidine concentrations (1% and 2%), with most infants receiving only one application. The third focused on very low birthweight infants and evaluated the combined effect of 1% chlorhexidine plus emollient compared to either alone or standard of care. All three trials found reductions in skin bacterial colonization with chlorhexidine application, but direct comparisons are difficult due to different target populations and application strategies.

**Added value of this study**

The largest burden of hospital-acquired infection is likely to be observed in low birthweight infants requiring prolonged neonatal unit stays, including periods of intensive or high-dependency care. Therefore, we aimed to establish the optimum application of chlorhexidine in terms of concentration (0.5%, 1%, or 2%), emollient use (CHG ± sunflower oil) and application frequency (working days or alternate working days) achieving the best balance of feasibility, safety and effectiveness for testing in a future pragmatic trial assessing clinical outcomes. Our pilot trial confirms the clinical safety of all tested chlorhexidine regimens, with no regimen clearly emerging as more or less effective; however, use of 2% chlorhexidine is unlikely have an added effect on colonisation and combining chlorhexidine bathing with application of emollient may improve skin condition in this vulnerable population. Of note, infants in the trial were rapidly colonised by Enterobacterales, including multidrug-resistant strains, in the first few days of life regardless of receipt of skin antisepsis.

**Implications of all the available evidence**

Neonates are rapidly colonised with potentially pathogenic bacteria in hospital settings, but there is very limited evidence on effective infection prevention and control measures impacting clinically important outcomes of sepsis and mortality. Cheap, safe and acceptable strategies likely effective for at risk infants globally, including in LMICs, are urgently required. The effectiveness of a combination of CHG and sunflower oil on reducing sepsis or mortality in high-risk low birth weight infants could feasibly be assessed in a large trial, with our findings providing reassurance on safety but also reinforcing the need for identifying suitable interventions to protect vulnerable neonates requiring hospital care. The relatively late start application of emollient, at a mean of 3.8 days of life, may have reduced the impact of the intervention although no subgroup effects were detected.

## Introduction

Neonatal sepsis is a substantial cause of neonatal mortality,^1^ especially in low and middle income countries (LMIC), with an estimated 3 million cases worldwide each year, and 10–20% mortality.^2^ Hospital-acquired infection (HAI) in LMIC accounts for a substantial proportion of neonatal sepsis,^3^ and is also an important driver of increasing trends in antimicrobial resistance (AMR),^4^ particularly in neonates,^5^ with multidrug-resistant Gram-negative bacilli of particular concern. With increasing HAI and decreasing treatment options, new prevention strategies are urgently needed to achieve Sustainable Development Goal targets for reducing neonatal mortality.

Colonisation with pathogenic bacteria precedes invasive infections, and preterm low birthweight neonates with immature skin are particularly vulnerable to trans-epithelial bacterial invasion. Antiseptics such as chlorhexidine gluconate (CHG), which reduce bacterial load on the skin, may hence reduce the risk of neonatal sepsis, thereby reducing neonatal mortality. CHG is active against Gram positive and Gram negative organisms and is commonly used for skin disinfection in neonates for invasive procedures,^6^ but wider skin application could potentially prevent colonisation with multi-resistant hospital-acquired organisms.

Randomised trials have demonstrated that 4% CHG cord application reduces mortality in neonates in community settings with high background neonatal mortality rates^7^ and CHG skin application may have a similar effect by reducing bacterial colonisation,^8^ but recent systematic reviews of CHG skin application for reducing neonatal sepsis and mortality have been inconclusive.^7^^,^^9^ The antiseptic effect of CHG is likely temporary,^10^ therefore more frequent application of higher concentrations may be more effective in reducing bacterial colonisation. However, this may lead to a greater risk of complications such as skin damage, especially in preterm neonates,^11^^,^^12^ and the trade-off between greater efficacy with more frequent application, and skin safety, is unclear.

Emollient application may improve skin barrier function, and sunflower oil, among other vegetable oils, has shown potential to reduce episodes of neonatal sepsis and improve growth in preterm neonates^13^ and, although recent systematic reviews of emollients for mortality reduction have been inconclusive,^14^^,^^15^ the WHO has recently made a conditional recommendation for use of emollient therapy.^16^ The potential benefit of combining both CHG and emollient requires further study. This pilot trial aimed to investigate which concentration of CHG, used at what frequency, with or without emollient, would best balance risks and benefits such that its safety and effectiveness could be tested in a large randomised trial with neonatal mortality as the primary endpoint in hospital-based LMIC populations.

## Methods

### Study design

The trial used an unblinded, randomised controlled factorial design with equal allocation across 12 intervention arms and 1 control arm (Supplementary S1). The control arm was included as a benchmark to be able to distinguish between all antiseptics being equally effective and equally ineffective.

Neonates were recruited in two centres in South Africa (Tygerberg Hospital, Cape Town) and Bangladesh (Bangladesh Shishu Hospital and Institute (formally, Dhaka Shishu Hospital), Dhaka with implementation by Child Health Research Foundation (CHRF)), selected from an existing research network conducting the NeoObs observational study of neonatal sepsis.^17^ Both centres have extensive experience in neonatal trials including chlorhexidine and emollient interventions. Neither site practised skin-to-skin care at the time the trial was conducted. The trial received ethical approval from both sites and the Research Ethics Committee at St George’s University of London, UK (2020.0059). The protocol and statistical analysis plan (SAP) are available at https://www.isrctn.com/ISRCTN69836999, and also available in the Supplementary materials. There were no major deviations from protocol.

### Participants

Eligible neonates were aged 1–6 days (post-natally) at randomisation with gestational age ≥ 28 weeks at birth and birth-weight (or current weight if unknown) 1000–1999 g. Additionally, parents had to be willing to avoid routine use of emollients other than allocated at randomisation. Exclusion criteria were poor skin condition (skin score 2 or more in any of three domains; Supplementary S2), known congenital or acquired skin disorder or defect at randomisation, anticipated length of hospital stay <7 days, and CHG or emollient application determined inappropriate in the opinion of the enrolling clinician. Parents of eligible neonates were approached for consent after birth. Written informed consent was obtained from all neonates’ guardians or parents prior to trial entry.

### Randomisation and masking

Neonates were randomized using a list computer-generated by the Trial Statistician (MNC) using random permuted blocks of size 13 (12 treatment arms plus control) stratified by site. Neonates were enrolled by in-hospital clinicians and allocation was assigned using the randomization module in a secure, web-based RedCAP database. The system had controlled access with an authorised username and password; only delegated and trained members of the site team had permission to randomise neonates into the trial and were given access to the randomisation system. Due to the nature of the interventions, blinding to frequency or emollient was not possible and blinding to concentration was not deemed feasible given the small team running the pilot trial. However, the efficacy co-primary endpoint was assessed by laboratory staff blinded to randomisation.

### Procedures

The intervention arms assessed three different concentrations of aqueous CHG (0.5%, 1% and 2%), with or without emollient (sunflower oil) across two different application schedules (working days vs alternate working days). The control arm received neither CHG nor sunflower oil (current standard of care). Alternate working days were defined in the trial protocol as alternate weekdays starting on the first weekday of each week, depending on the standard working pattern in each country (Sunday in Bangladesh, Monday in South Africa). CHG was applied to the whole body with soaked cotton wool, excluding the face and scalp, and consistency between sites was ensured by the creation of video procedures. A teaspoon (4 g) per kg of sunflower oil (commercially available Sunflower natural carrier oil (*Helianthus annuus*) from EOil.co.za a brand of Amp Trading for skin care with >60% Linoleic acid) was applied with a gloved hand from the neck downwards. Treatment and Follow-up were daily until the earlier of either discharge or day-14, with a final phone call at day-28 (Supplementary S3).

### Outcomes

Two co-primary outcome measures assessed safety and efficacy. Safety was assessed via a modified neonatal skin condition score adapted from Lund & Osbrne,^18^ which included domains for skin dryness, erythema and breakdown, graded 0–4 based on percentage body surface area affected (Supplementary S2). This was assessed before each CHG application, or on alternate working days in controls, until the earlier of day 14 or discharge, analysed as the daily change from baseline. Efficacy was assessed via skin bacterial load, defined as total (sum) log colony forming units (CFUs; Supplementary S4) in the nose (1 swab), cervical skin folds and umbilicus (1 pooled swab), and peri-rectal area (1 swab), analysed as the change from randomisation to day-3 and day-8. All outcomes were assessed at the respective swab collection sites. In this pilot trial, skin bacterial load reduction was chosen as a potential surrogate marker, whereas a larger trial would examine clinical outcomes of sepsis and mortality.

Secondary outcome measures were temperature after CHG application, acquisition and loss of specific bacterial species at day-3 and day-8, and Serious Adverse Events (SAE) measured until day-28 (via phone contact if discharged). Temperature was analysed as change from before application to after application (after swabs taken in controls), change from baseline after randomisation, and as graded toxicity. The pre-specified bacterial groups/species of interest were Enterobacterales, *Acinetobacter baumnnii*, *Pseudomonas aeruginosa, Staphylococcus aureus*, Beta haemolytic streptococci (groups A and B), *Enterococcus* spp, and *Candida* spp. DAIDS grading was used to define SAEs, including specifically solicited adverse events such as skin damage, hypothermia, and neurotoxicity (Supplementary S5).^19^ Exploratory efficacy outcomes specified in the Statistical Analysis Plan were log CFUs of Gram positive, Gram negative and yeast species. Post-hoc resistance rates were determined for specific organisms and antibiotics.

### Statistical analysis

182 neonates with complete day-3 and day-8 swabs provided 90% power to detect a difference of 0.66 standard deviations (SDs) between concentrations and 0.47 SDs between different frequencies and emollient (two-sided α = 0.05) (80% power for 0.58 and 0.41 SDs, respectively), and 90% power to detect a difference of 1.04 SD between each concentration and control, and 0.96 SD between each level of frequency/emollient and control (80% power for 0.92 and 0.96 SDs, respectively). For each drug/frequency combination the sample size provided 90% power to detect a difference of 1.70 SDs between treatment and control and 1.97 SDs between each treatment combination (80% power for 1.52 SD and 1.78SDs respectively). This sample size was judged by the clinical team to be sufficient given previous observed data showing decreases in log CFU from baseline to 24 h ranging from 0.2 SD to 2 SD.^10^^,^^20^ A Data Monitoring Committee assessed safety endpoints halfway through recruitment.

All neonates with at least one post-baseline measurement were included in the analyses of each co-primary outcome. Skin score change was analysed using mixed effects ordinal logistic regression as >80% responses were zero. Total log_10_ CFU change was analysed using mixed effect models with normally distributed errors. Both models used robust variance estimation, fitted individual as a random effect and included fixed effects for concentration (0.5%, 1%, 2%); application frequency (week days, alternate week days); emollient (yes, no); site (Bangladesh, South Africa); day of assessment (day-3/day-8 for efficacy and day of trial for safety); and baseline value (estimate not interpretable due to regression to the mean; fitted as a linear term as mfp modelling did not detect any departures from linearity), making comparisons using Wald tests. The distribution of model residuals were checked visually for departures from Normality. The reference group was 0.5% CHG, applied alternate working days with no emollient as this strategy would be most straightforward to implement. As the goal was to identify an optimal cleansing regimen, the standard of care arm was included only as a benchmark, with smaller sample size, and was therefore not used as the reference. Skin score analysis used all available data although scores recorded closest to day-3 and day-8 are presented for comparison with CFUs.

Acquisition and loss of specific bacterial species was analysed using equivalent binomial generalised linear mixed models (logit link) with change from baseline as the response. Temperature change from baseline to post-application and from pre-to post-application were analysed in separate models as per log_10_ CFU. SAEs and AEs were summarised overall and by MedDRA System Order Class (SOC) and Preferred Term (PT) and compared using exact logistic models.

Bayesian ACCEPT analysis^21^ estimated the probability that the efficacy co-primary outcome (pre-specified) and the percentage with any SAE (post-hoc requested by the DMC) truly differed from the reference group by different values (priors in Supplementary S6; elicited before the trial for log_10_ CFU, uninformative for SAEs as trial team had already reviewed blinded SAEs). ACCEPT analyses were not done on skin scores as differences between the arms were very small.

Post-hoc resistance rates in all neonates were estimated using mixed effects logistic regression with response resistance yes/no and fixed effects of randomised group (as above), site and day as a numeric variable. Analyses of resistance given growth used logistic regression as mixed effects models did not converge due to the low sample sizes. All analyses were ‘intention-to-treat’ (‘treatment policy’ in the Estimands framework with at least baseline and one other outcome measure) and there was no adjustment for multiple testing.

Interactions between drug, frequency and emollient were assessed one at a time in separate models and sensitivity of the models was assessed by analysing the first sample 48 h post-baseline only. Additional sensitivity analyses specified in the statistical analysis plan (SAP) were the final sample for each individual, days since last CHG application fitted as an additional continuous variable and a binary factor of whether or not the baby received antibiotics in the 24 h before assessment. Subgroup analyses specified in the SAP were antibiotic exposure pre-enrolment, rupture of membranes during delivery, very low birth weight (<1500 g) as a binary factor and age at enrolment as a two-level factor split at the median. Subgroup analyses fitted the interaction between each arm and the relevant subgroup factor one at a time in separate models.

The NeoCHG trial was registered on the ISRCTN register as study ISRCTN69836999 (https://doi.org/10.1186/ISRCTN69836999). Results conform to CONSORT guidelines.

### Role of the funding source

The funder of the trial (MRC) had no role in trial design, data collection, data analysis, data interpretation, writing of the report or the decision to submit for publication. GARDP provided in-kind support to the trial, specifically input to the protocol development and manuscript but did not have any role in data collection, data analysis or writing the first draft of the report. MC and ASW had access to the dataset. MC and NR had final responsibility to submit for publication.

## Results

**Fig. 1:**
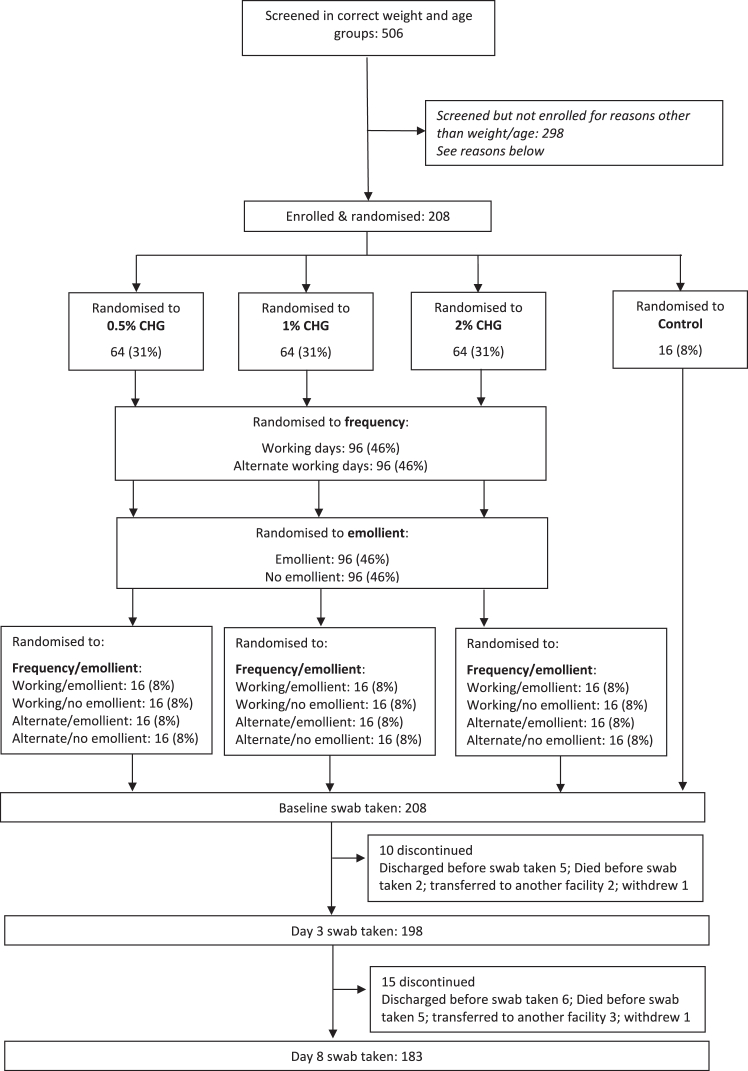
CONSORT diagram. Note that all babies that had a swab taken also had previous swabs taken. Reasons screened but not enrolled (excluding weight/age criteria): Expected to be discharged to another facility within 7 days 84 (28%); Expected to be discharged home before 7 days 75 (25%); Lab capacity related reasons 39 (13%); Not expected to survive 7 days 18 (6%); Parent/guardian did not consent (i.e. did not wish for their baby to participate in the trial) 16 (5%); Enrolment into another study 10 (3%); Known congenital or acquired skin disorder or defect at time of enrolment 5 (2%); Parents are not willing to avoid routine use of emollients other than those indicated by the randomisation 3 (1%); Poor skin condition (skin score of 2 or more in any of three domains) at the time of enrolment 3 (1%); Chlorhexidine/emollient application determined inappropriate in the opinion of the enrolling clinician 3 (1%); Other 42 (14%). Details of other reasons for screened but not enrolled: Could not reach parent/guardian for consent 18 (43%); Staff members on leave 9 (21%); Database issues 5 (12%); Parents do not speak languages used in trial 3 (7%); Baby is for minimal handling 1 (2%); Unknown 6 (14%).

Between 12th April 2021 and 18th January 2022, 208 neonates were randomised (Fig. 1), with all neonates having at least the baseline swab taken, 198/208 (95%) neonates having two swabs (baseline and day-3) taken and 183/208 (88%) having all three swabs taken (baseline, day-3 and day-8). Recruitment ended when the pre-specified sample size of at least 182 neonates with three swabs (baseline, day-3, and day-8) was reached.

Overall, 127/208 (61%) neonates were born by emergency C-section (Table 1; additional baseline data in Supplementary S7). At baseline, 139/208 (67%) neonates had received antibiotics since birth. 130/208 (62%) neonates were randomised in South Africa.

**Table 1:** Baseline characteristics.

var	Level/metric	Overall	0.5% CHG (N = 64)	1% CHG (N = 64)	2% CHG (N = 64)	Alternate wd (N = 96)	Working days (N = 96)	Emollient (N = 96)	No emollient (N = 96)	Control (N = 16)
Site	Bangladesh	78 (38%)	24 (38%)	24 (38%)	24 (38%)	36 (38%)	36 (38%)	36 (38%)	36 (38%)	6 (38%)
	South Africa	130 (62%)	40 (62%)	40 (62%)	40 (62%)	60 (62%)	60 (62%)	60 (62%)	60 (62%)	10 (62%)
Sex	Female	92 (44%)	27 (42%)	30 (47%)	28 (44%)	45 (47%)	40 (42%)	43 (45%)	42 (44%)	7 (44%)
	Male	116 (56%)	37 (58%)	34 (53%)	36 (56%)	51 (53%)	56 (58%)	53 (55%)	54 (56%)	9 (56%)
Age at randomisation (days)	Mean (SD)	3.8 (1.7)	3.8 (1.7)	3.7 (1.6)	4.0 (1.7)	3.6 (1.6)	4.0 (1.7)	3.8 (1.6)	3.8 (1.7)	3.9 (1.6)
Gestational age at birth (weeks)	Mean (SD)	31.8 (2.8)	31.5 (2.8)	32.0 (2.6)	32.2 (3.0)	31.9 (2.7)	31.9 (2.9)	32.0 (2.6)	31.8 (3.0)	31.1 (2.7)
Birth weight (g)	Mean (SD)	1447 (278)	1463 (283)	1440 (257)	1462 (291)	1452 (266)	1458 (287)	1450 (262)	1460 (291)	1349 (285)
Weight at randomisation (g)	Mean (SD)	1414 (275)	1426 (279)	1410 (256)	1425 (291)	1420 (267)	1420 (283)	1418 (266)	1422 (285)	1340 (283)
Total skin score at randomisation	Mean (SD)	0.10 (0.30)	0.06 (0.24)	0.12 (0.33)	0.06 (0.24)	0.11 (0.32)	0.05 (0.22)	0.10 (0.31)	0.06 (0.24)	0.25 (0.45)
Temperature at randomisation (oC)	Mean (SD)	36.7 (0.4)	36.7 (0.6)	36.7 (0.4)	36.7 (0.3)	36.6 (0.4)	36.7 (0.5)	36.7 (0.5)	36.6 (0.4)	36.8 (0.5)
Intrapartum antibiotics	Yes	171 (82%)	54 (84%)	52 (81%)	52 (81%)	82 (85%)	76 (79%)	79 (82%)	79 (82%)	13 (81%)
	No	33 (16%)	9 (14%)	10 (16%)	11 (17%)	11 (11%)	19 (20%)	15 (16%)	15 (16%)	3 (19%)
	Unknown	4 (2%)	1 (2%)	2 (3%)	1 (2%)	3 (3%)	1 (1%)	2 (2%)	2 (2%)	
Antibiotics since birth at baseline		139 (67%)	47 (73%)	37 (58%)	43 (67%)	59 (61%)	68 (71%)	60 (62%)	67 (70%)	12 (75%)
Antibiotics intrapartum or since birth		205 (99%)	64 (100%)	62 (97%)	63 (98%)	94 (98%)	95 (99%)	95 (99%)	94 (98%)	16 (100%)
Prematurity		195 (94%)	60 (94%)	61 (95%)	58 (91%)	92 (96%)	87 (91%)	90 (94%)	89 (93%)	16 (100%)
Mode of delivery	Elective (planned) caesarean section	9 (4%)	5 (8%)	1 (2%)	3 (5%)	5 (5%)	4 (4%)	4 (4%)	5 (5%)	
	Emergency caesarean section	127 (61%)	34 (53%)	41 (64%)	41 (64%)	61 (64%)	55 (57%)	62 (65%)	54 (56%)	11 (69%)
	Vaginal delivery (assisted)	5 (2%)	3 (5%)	1 (2%)		2 (2%)	2 (2%)	2 (2%)	2 (2%)	1 (6%)
	Vaginal delivery (spontaneous)	67 (32%)	22 (34%)	21 (33%)	20 (31%)	28 (29%)	35 (36%)	28 (29%)	35 (36%)	4 (25%)
Total log10 CFU at baseline	Mean (SD)	4.9 (3.0)	5.1 (3.0)	4.6 (3.0)	5.0 (2.9)	4.8 (3.1)	5.0 (2.9)	4.9 (3.0)	5.0 (3.1)	4.2 (3.1)

Note: neonates assigned to CHG (i.e. not assigned to Control) appear in the tables three times—split by CHG concentration, split by frequency and split by emollient/no emollient. Wd = working days.

There were no instances of CHG or emollient being given outside times indicated by the protocol (Supplementary S8). Temporary treatment interruptions occurred for 49/192 (26%) neonates randomised to CHG, mainly on public holidays 38/49 (78%) with the remainder due to the neonate’s condition or parent/guardian refusal. Permanent treatment discontinuation was reported in four of 208 neonates (2%), three when the neonate was too ill for CHG treatment and one when the neonate was abandoned. There were no CHG or emollient overdoses or use of non-trial emollient. No neonates in the control arm received CHG or emollient and no neonates in the non-emollient arm received emollient. 60/208 (29%) neonates started antibiotics after baseline with 139 receiving any antibiotics post-baseline.

There was clear separation between the frequency arms by day-3 swab: 88/88 (100%) neonates randomised to alternate working days had one CHG application and 88/94 (94%) neonates randomised to working days had two CHG applications before the swab was taken (p < 0.0001; Supplementary S8). Of those neonates with a day-8 swab, 79/79 (100%) neonates randomised to alternate working days had two or three applications before the swab was taken and 80/88 (91%) neonates randomised to working days had four or five applications (p < 0.0001). The median number of days with skin score assessments was 5 (IQR: 3,6) in the alternate working days arm and 8 (IQR: 5, 10; p < 0.0001) in the working days arm; the median day of last skin score assessment was day 10 (IQR: 5, 12; p = 0.62) in both. In contrast there was no evidence of difference in number of applications or days skin scores were assessed by CHG concentration (p > 0.5) or emollient (p > 0.38).

**Fig. 2:**
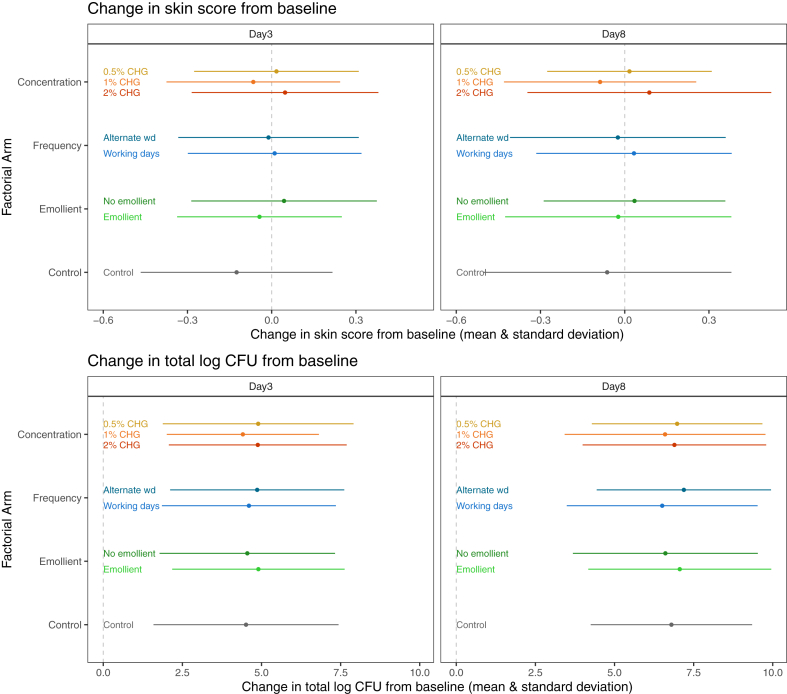
Co-primary outcomes by arm. Note that data in the non-control arms appear in graph multiple times. Skin score was on a scale from 0 (normal) to 12 (very bad). Vertical grey dotted line shows no change. Wd = working day.

Skin scores (safety co-primary outcome) were low throughout follow-up: of all scores recorded before any CHG application, 1269/1436 (81%) were zero, 166/1436 (11%) were one and there was only one score of two, out of a possible total score of 12 (Supplementary S9). There was no evidence of a difference in change in skin score between CHG concentration (p = 0.27), frequency (p = 0.33) or emollient (p = 0.23) although numerically skin scores were lower with 1% CHG (than with 0.5% or 2% CHG), more frequent application with emollient, and for the control group (Table 2; Fig. 2). There was no evidence of interactions between randomised groups (heterogeneity p > 0.4; Supplementary S10) or by pre-specified subgroups (p > 0.01; Supplementary S11).

**Table 2:** Primary and secondary outcomes with modelled effect sizes averaged across both days.

Arm	Change baseline to d3 Mean (SD) [N]	Change baseline to d8 Mean (SD) [N]	Modelled effect size vs reference (95% CI)	p-value	Overall p-value for drug
Skin score change					
0.5% CHG (N = 64)	0.02 (0.29) [59]	0.02 (0.29) [59]	–		0.27
1% CHG (N = 64)	−0.07 (0.31) [61]	−0.09 (0.34) [57]	−0.28 (−1.19, 0.63)	0.55	
2% CHG (N = 64)	0.05 (0.33) [63]	0.09 (0.43) [57]	0.48 (−0.43, 1.39)	0.30	
Alternate wd (N = 96)	−0.01 (0.32) [88]	−0.02 (0.38) [82]	–		
Working days (N = 96)	0.01 (0.31) [95]	0.03 (0.35) [91]	−0.38 (−1.14, 0.38)	0.33	
No emollient (N = 96)	0.04 (0.33) [91]	0.03 (0.32) [86]	–		
Emollient (N = 96)	−0.04 (0.29) [92]	−0.02 (0.40) [87]	−0.46 (−1.22, 0.29)	0.23	
Control (N = 16)	−0.12 (0.34) [16]	−0.06 (0.44) [16]	−0.93 (−2.29, 0.44)	0.18	
Temperature change from baseline to post-application					
0.5% CHG (N = 64)	−0.1 (0.5) [58]	−0.2 (0.6) [58]	–		0.79
1% CHG (N = 64)	−0.2 (0.5) [58]	−0.1 (0.5) [56]	−0.0 (−0.1, 0.1)	0.83	
2% CHG (N = 64)	−0.0 (0.5) [61]	−0.1 (0.5) [55]	−0.0 (−0.1, 0.0)	0.50	
Alternate wd (N = 96)	−0.1 (0.5) [84]	−0.1 (0.5) [78]	–		
Working days (N = 96)	−0.1 (0.5) [93]	−0.1 (0.6) [91]	0.0 (−0.0, 0.1)	0.62	
No emollient (N = 96)	−0.1 (0.5) [86]	−0.1 (0.5) [84]	–		
Emollient (N = 96)	−0.1 (0.5) [91]	−0.2 (0.5) [85]	−0.0 (−0.1, 0.1)	0.97	
Temperature change pre- to post-application					
0.5% CHG (N = 64)	−0.1 (0.4) [58]	−0.1 (0.3) [58]	–		0.76
1% CHG (N = 64)	−0.2 (0.2) [58]	−0.1 (0.3) [56]	−0.0 (−0.2, 0.1)	0.68	
2% CHG (N = 64)	−0.1 (0.3) [61]	−0.1 (0.3) [55]	−0.1 (−0.2, 0.1)	0.46	
Alternate wd (N = 96)	−0.2 (0.3) [84]	−0.1 (0.3) [78]	–		
Working days (N = 96)	−0.1 (0.3) [93]	−0.1 (0.3) [91]	0.1 (−0.0, 0.2)	0.25	
No emollient (N = 96)	−0.1 (0.3) [86]	−0.1 (0.2) [84]	–		
Emollient (N = 96)	−0.1 (0.3) [91]	−0.1 (0.3) [85]	0.1 (−0.0, 0.2)	0.15	
Total change in log10 CFU					
0.5% CHG (N = 64)	1.5 (3.1) [59]	3.6 (3.9) [58]	–		0.39
1% CHG (N = 64)	1.3 (3.0) [61]	3.6 (4.5) [52]	−0.4 (−1.1, 0.3)	0.23	
2% CHG (N = 64)	1.6 (3.1) [62]	3.7 (4.1) [57]	0.0 (−0.6, 0.6)	0.96	
Alternate wd (N = 96)	1.7 (3.2) [88]	4.2 (4.1) [79]	–		
Working days (N = 96)	1.3 (2.9) [94]	3.1 (4.2) [88]	−0.4 (−0.9, 0.2)	0.17	
No emollient (N = 96)	1.3 (3.1) [90]	3.4 (4.1) [85]	–		
Emollient (N = 96)	1.7 (3.1) [92]	3.8 (4.2) [82]	0.4 (−0.2, 0.9)	0.18	
Control (N = 16)	1.7 (3.1) [16]	4.0 (3.8) [16]	−0.2 (−1.3, 0.9)	0.73	
Change in gram negative log10 CFU					
0.5% CHG (N = 64)	0.9 (3.2) [59]	3.0 (3.5) [58]	–		0.018
1% CHG (N = 64)	0.9 (2.0) [61]	2.8 (3.3) [52]	−0.5 (−1.1, 0.1)	0.12	
2% CHG (N = 64)	1.8 (2.7) [62]	3.5 (2.9) [57]	0.4 (−0.2, 1.1)	0.21	
Alternate wd (N = 96)	0.9 (2.7) [88]	3.2 (3.3) [79]	–		
Working days (N = 96)	1.4 (2.7) [94]	3.1 (3.2) [88]	0.1 (−0.4, 0.6)	0.66	
No emollient (N = 96)	1.1 (2.9) [90]	3.0 (3.3) [85]	–		
Emollient (N = 96)	1.3 (2.5) [92]	3.3 (3.2) [82]	0.2 (−0.3, 0.8)	0.36	
Control (N = 16)	0.6 (1.7) [16]	2.6 (3.6) [16]	−0.2 (−1.4, 0.9)	0.70	
Change in gram positive log10 CFU					
0.5% CHG (N = 64)	1.4 (2.8) [59]	3.2 (3.8) [58]	–		0.14
1% CHG (N = 64)	0.8 (3.1) [61]	3.2 (4.4) [52]	−0.6 (−1.3, 0.1)	0.085	
2% CHG (N = 64)	0.9 (3.2) [62]	2.8 (3.9) [57]	−0.6 (−1.3, 0.2)	0.12	
Alternate wd (N = 96)	1.4 (3.4) [88]	3.5 (4.0) [79]	–		
Working days (N = 96)	0.7 (2.7) [94]	2.6 (4.0) [88]	−0.4 (−1.0, 0.2)	0.20	
No emollient (N = 96)	1.0 (3.0) [90]	3.0 (3.9) [85]	–		
Emollient (N = 96)	1.0 (3.1) [92]	3.1 (4.2) [82]	0.2 (−0.4, 0.8)	0.58	
Control (N = 16)	1.1 (3.2) [16]	3.5 (3.9) [16]	−0.8 (−2.0, 0.4)	0.18	
Change in yeast log10 CFU					
0.5% CHG (N = 64)	0.1 (0.3) [59]	0.0 (0.1) [58]	–		0.16
1% CHG (N = 64)	0.1 (0.9) [61]	0.1 (0.4) [52]	0.1 (−0.0, 0.2)	0.19	
2% CHG (N = 64)	0.1 (0.7) [62]	0.1 (0.5) [57]	0.1 (−0.0, 0.2)	0.11	
Alternate wd (N = 96)	0.0 (0.3) [88]	0.0 (0.4) [79]	–		
Working days (N = 96)	0.2 (0.9) [94]	0.1 (0.3) [88]	0.1 (−0.0, 0.2)	0.056	
No emollient (N = 96)	0.1 (0.4) [90]	0.0 (0.2) [85]	–		
Emollient (N = 96)	0.1 (0.9) [92]	0.1 (0.4) [82]	0.1 (−0.0, 0.2)	0.086	
Control (N = 16)	0.0 (0.0) [16]	0.1 (0.6) [16]	0.1 (−0.0, 0.3)	0.14	

Note: estimates are presented vs 0.5% CHG applied on alternate weekdays without emollient as the reference as this regimen would be the most straightforward to implement.

Wd = working days, Skin score analysis used all available data and scores recorded closest to day-3 and day-8 are presented for comparison with CFUs.

There was no evidence of differences between randomised concentration, frequency or emollient in change in temperature from baseline to pre-application (p > 0.5) or change from pre-to post-application (p > 0.15; Table 2). Overall, the mean temperature 30 min after CHG application was 0.10 °C lower [95% CI: (−0.12, −0.07); p < 0.0001] than before CHG application.

**Fig. 3:**
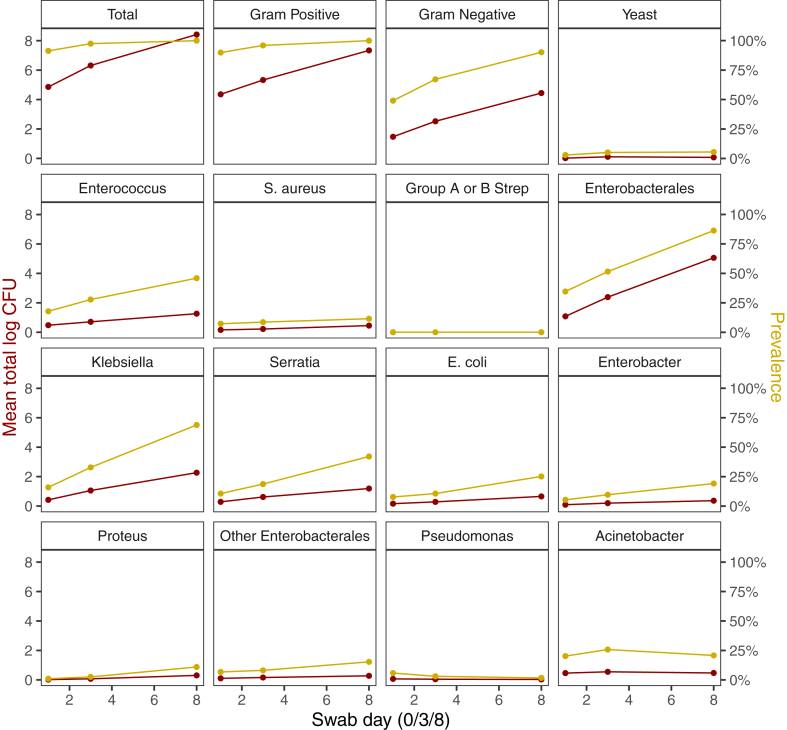
Overall mean CFU (red, left y axis) and prevalence (yellow, right y axis) of grouped and individual species over time. All Yeast detected was Candida spp.

Overall, the mean total log_10_ CFU (efficacy co-primary outcome) was 4.86 (SD = 3.01) at baseline, 6.31 (3.10) at day-3 and 8.42 (2.62) at day-8 (Fig. 3). There was no evidence of a difference in change in total log_10_ CFU by CHG concentration (p = 0.39), frequency (p = 0.17) or emollient (p = 0.18) or between the reference arm (0.5% CHG, alternate working days without emollient) and control (p = 0.73), although numerically bacterial load was lower with 1% CHG, more frequent application (working days) and no emollient (Table 2; Fig. 2). Numerically lower bacterial load with 1% CHG compared to 2% CHG was also apparent in gram negative (estimate = −0.5, 95%CI: −1.1, 0.1; p = 0.12) and gram-positive organisms (estimate = −0.6, 95%CI: −1.3, 0.1; p = 0.085) but not in yeast (estimate = 0.1, 95%CI: 0.0, 0.2; p = 0.19; Table 2). Bayesian ACCEPT analysis on total log10 CFU estimated the probability of 1% CHG being more effective than 0.5% CHG as 88%, 93% and 70% under non-informative, optimistic and sceptical priors, respectively. The probabilities dropped to 41%, 13% and 2% for 1% CHG being more effective than 0.5% CHG by at least 0.5 log_10_ CFU (Supplementary S12).

There was no evidence of interactions between randomised groups (heterogeneity p > 0.4; Supplementary S13) or by pre-specified subgroups (p > 0.02; Supplementary S14). Sensitivity analysis adjusting for receipt of IV antibiotics in last 24 h (111/198 (56%) at day 3 swab and 76/183 (42%) at day 8 swab) showed log10 CFU associated with a reduction of 2.4 (95% CI: −3.3, −1.6, p < 0.0001; Supplementary S15) with antibiotic in the last 24 h but effects of randomised groups were broadly similar and no other sensitivity analyses resulted in qualitatively different conclusions (p > 0.019).

There was increased yeast colonisation with emollient (p = 0.014; Supplementary S15) but this was not reflected in differences in mean CFU (p = 0.086; Table 2). There was no evidence of substantial differences between concentrations (p > 0.4), frequencies (p > 0.08), or emollient (p > 0.25) in presence/absence of the other focal species (Supplementary S16). Overall, prevalence of colonisation with Enterobacterales was notably high, reaching >80% overall by day-8 (Fig. 3).

At day-8, ampicillin-resistant *E. coli* were detected in 36/183 (20%) neonates with swabs taken and 36/49 (73%) of *E. coli* isolated (Table 3; all tested resistance rates available in Supplementary S17). As a percentage of neonates, resistance rates for *Klebsiella* spp were 72/183 (39%) for cefotaxime, 69/183 (38%) gentamicin and 17/183 (9%) meropenem and as a percentage of organisms were 72/129 (56%) cefotaxime, 69/129 (53%) gentamicin and 17/129 (13%) meropenem. Increased resistance at the level of the neonate over time was driven by increasing colonisation over time and not by a change in the composition of the bacterial population.

**Table 3:** Resistance rates overall (for all processed samples) and given growth (for only those samples with specified species detected).

Bug: drug	Overall (proportion of neonates)				Given growth (proportion of organisms)			
	Baseline	Day 8 swab	Odds ratio (95% CI)	p-value	Baseline	Day 8 swab	Odds ratio	p-value
*E. coli*: ampicillin	11/208 (5%)	36/183 (20%)	1.37 (1.18, 1.59)	<0.0001	11/17 (65%)	36/49 (73%)	0.94 (0.77, 1.13)	0.49
Klebsiella: cefotaxime	22/208 (11%)	72/183 (39%)	1.62 (1.39, 1.89)	<0.0001	22/32 (69%)	72/129 (56%)	0.94 (0.84, 1.04)	0.22
Klebsiella: gentamicin	20/208 (10%)	69/183 (38%)	1.62 (1.37, 1.91)	<0.0001	20/32 (62%)	69/129 (53%)	0.96 (0.87, 1.06)	0.46
Klebsiella: meropenem	8/208 (4%)	17/183 (9%)	1.38 (1.08, 1.76)	0.0098	8/32 (25%)	17/129 (13%)	1.09 (0.85, 1.4)	0.51

The intercept was 1% CHG applied once without emollient. There was no evidence of differences between the intercept and control in any model. Supplementary information for data on all organisms and all drugs tested.

Overall, 22 SAEs occurred in 21 neonates (Supplementary S18 and S19), including 12 deaths, 4 (6%), 5 (8%) and 3 (5%) in the 0.5%, 1% and 2% CHG arms (exact p = 1.0) and 1 (6%) in the control arm; frequency p = 0.77, emollient p = 0.77). Most SAEs were infections and infestations (9), then gastro-intestinal disorders (4). Of those neonates with sepsis, no blood cultures were positive. There were no CHG-related SAEs, emollient-related SAEs, grade 3 or 4 skin scores or grade 3 or 4 hypothermia.

Post-hoc exploratory ACCEPT analyses of SAEs estimated an 89% probability that SAE rates were higher in 1% CHG than 0.5% CHG, and a 74% probability that the SAE rate was at least 5 percentage points higher (Supplementary S20).

## Discussion

The NeoCHG pilot trial aimed to determine the optimal concentration and frequency of aqueous CHG skin application, with or without emollient, to be considered as part of a bundle of interventions in a future trial aiming to reduce mortality in high-risk hospitalised neonates in LMICs. This trial provided reassurance on the safety of whole-body aqueous CHG application for concentrations ranging from 0.5% to 2%. Overall skin scores were low, there was no evidence of differences in safety outcomes between the arms, and no severe skin reactions were observed in this vulnerable population of very low birthweight infants although relatively rare skin reactions would require a larger trial to robustly detect. Regarding the effect on total bacterial skin colonisation density, there was no compelling evidence for any particular concentration or frequency of CHG application, although the relationship between skin colonisation and sepsis is unclear. Emollient application similarly had no substantial impact on total bacterial colonisation, although did appear to improve skin condition in very low birth weight babies receiving CHG.

There was a trend towards lower overall bacterial colonisation density using 1% vs 0.5% CHG, which was also apparent across gram-positive and gram-negative organisms but not *Candida* spp. Interestingly, we observed a trend towards higher colonisation density in the 2% vs 1% CHG arm, suggesting that increasing CHG concentrations higher than 1% may be unlikely to further reduce bacterial colonisation. Previous studies have shown greater reductions in bacterial colonisation with higher concentrations of CHG, up to 2%,^22^^,^^23^ However, while many studies measured bacterial colonisation immediately after CHG application,^7^^,^^22^ this pilot trial specifically examined effects sustained over time which are more relevant for preventative CHG bathing. The findings of this study suggest that alternate day washing with 1% CHG and sunflower oil application may be a favourable regimen to assess in a future trial with a mortality endpoint and with assessment of skin microbiome effects and blood levels of CHG.

Rapid increase in gram-negative bacterial colonisation in the first 10 days of life was observed, regardless of treatment regimen. By day-3 and day-8, over 40% and 80% of infants, respectively, were colonised by *Enterobacterales*, most commonly *Klebsiella* spp., followed by *Serratia* spp. and *E. coli*. *Acinetobacter* spp was also common, while *S. aureus* was less frequent, and Group B Streptococcus was not identified. The differential impact of chlorhexidine on gram positive and gram-negative bacteria may have influenced this distribution.^24^ Importantly, resistant bacteria were highly prevalent among colonising isolates, including one in ten infants found to be colonised by carbapenem-resistant *Klebsiella* spp. by day 8. Increased colonisation by resistant bacteria over time was likely driven by higher colonisation densities overall rather than within-patient selection. Colonising species were comparable between the arms except for *Candida* spp., where colonisation was higher in the emollient arm. This may require further consideration since neonatal invasive candidiasis has been shown to be an important cause of neonatal sepsis cases in LMICs and associated with a >20% mortality.^25^

Colonisation density and dominance of certain bacteria was unsurprisingly influenced by recent or on-going antibiotic exposure. Antibiotics could modify the impact of CHG application, and the high antibiotic exposure in this trial differs from the community-based populations in whom the evidence base for mortality reduction with CHG cord application was demonstrated.^26^, ^27^, ^28^ Nevertheless, our findings are likely generalisable to similar high-risk hospital-based populations who would be targeted with CHG application in a future trial.

The factorial design of our pilot trial enabled simultaneous assessment of CHG whole body skin application frequency and concentration, as well as emollient application. Whole body application of CHG and sunflower oil have been individually identified as potentially reducing risk of sepsis and mortality in hospital-based populations, although recent systematic reviews have identified the need for more data.^7^^,^^9^^,^^14^ Bathing with 2% CHG as part of a bundle of infection prevention and control (IPC) interventions reduced the combined endpoint of bloodstream infection or mortality in infants ≥ 1.5 kg,^29^ with a suggestion of an independent association with fewer bloodstream infections on secondary analysis.^30^ Studies of sunflower oil have suggested a potential decrease in sepsis in hospitalized newborns,^13^ and improved growth in the community settings.^31^ Other emollients have also shown promise, such as coconut oil^32^, ^33^, ^34^ and Aquaphor,^35^ and a recent pilot study showed improved skin condition when combining CHG with Aquaphor, although there was also a suggestion of higher colonisation with *S. aureus* in the emollient group.^36^

We were able to demonstrate that the tested approaches to CHG body washing and emollient application, as well as safety assessments and sample collection to track bacterial skin colonisation, were feasible and safe in the context of two busy LMIC neonatal units. Trial limitations include that the co-primary efficacy outcome, overall bacterial colonisation density, is not a well-established surrogate marker for neonatal sepsis or mortality, and reduced bacterial colonisation density may not necessarily correlate with clinical outcomes. Skin bacterial load is therefore not a suitable outcome for larger future trials aiming to reduce mortality. Bacterial colonisation density as measured in this trial also included both pathogenic and non-pathogenic organisms, making it difficult to determine any specific effects for clinically relevant species. However, secondary analyses of species groups (gram-positive and gram-negative) and individual species did not reveal any substantial differences in colonisation patterns. Further analyses using molecular methods might provide additional relevant information but was not possible in this study.

Lack of statistical evidence of effect in our study may be partly driven by low sample sizes and high variation in logCFU, leading to low power to detect genuine differences. However, effect estimates were also small in magnitude so power may not be the only contributing factor and highlights the disparity in results from the literature and this study, perhaps driven by geographical factors. Late application at a mean of 3.8 days of life may also have reduced the impact of the intervention although no subgroup effects were detected. In terms of the studied CHG application regimens, weekdays or alternate weekdays were investigated to reflect considerable pressures on clinical staff, especially on weekends, in many LMIC neonatal units. Daily application regimens may have slightly different effects on bacterial colonisation density. Given that the goal of this pilot trial was to establish optimal approaches to CHG whole body skin application, it is not possible to assess how this compares to other infection prevention and control interventions of interest for LMIC neonatal care. Additionally, the formulation and application regimen of sunflower oil may have impacted results, and although once daily or alternate day application were felt to be most feasible and generalisable, this frequency may be less effective than multiple times per day. Furthermore, skin scores are an imperfect measure, due partly to inter-observer variability and an unblinded intervention, and although our score was modified to provide more specificity regarding severity of skin condition changes than previous scores, the possibly of the score lacking sensitivity to detect more minor changes remains. In addition, this study did not measure blood levels of chlorhexidine, the significance of which remains unclear.^37^

This pilot trial suggests that combining CHG skin application with sunflower oil emollient is likely safe and feasible to be taken forward in a larger trial with clinical outcomes. The findings demonstrate rapid and dense bacterial colonisation in the first few days of life regardless of CHG application, and do not provide overwhelming evidence in favour of a particular CHG concentration or application regimen or emollient, nor do they provide compelling evidence to support the choice of bacterial load reduction as a feasible target for reducing sepsis. Nevertheless, the findings suggest that 1% CHG appears safe, and we note a lack of clear benefit of increasing CHG concentration beyond 1% CHG, which may also be less acceptable to many clinicians. In addition, results suggest that combining CHG with sunflower oil may improve skin condition in very low birthweight infants, although a possible increase in yeast colonisation requires further exploration. The potential mortality impact of skin antisepsis and emollients in high-risk neonates in LMIC hospital settings, as part of a multimodal infection prevention & control strategy including other interventions with recent evidence such as skin-to-skin care and immediate kangaroo mother care^38^ requires further exploration in randomised controlled trials which can be informed by this pilot trial.

## Contributors

NeoCHG was conceptualised by MS, NR, JB, MC and ASW. The protocol and trial were designed by all co-authors. The clinical trial was overseen by KLB, FS and PS. NeoCHG was conducted in South Africa by AB, AD and AF and in Bangladesh by MH, MSI and SS. Microbiological samples were processed by AW. MNC conducted the statistical analysis. MC, NR FS, KLB and PS wrote the first draft of the paper. All authors contributed to subsequent drafts, read, and approved the final version of the manuscript. MNC and ASW had full access to, and verified, all the data in the trial. The corresponding author was responsible for the final decision to submit for publication and has full access to all study data.

## Data sharing statement

Sharing of data will be considered based on a detailed proposal which should include aims, methods and a statistical analysis plan. Requests will be checked for compatibility with regulatory and ethics committee requirements as well as with compatibility with the participant informed consent. Proposals should be addressed to the corresponding author and will be evaluated by the Sponsor.

## Declaration of interests

AD reports grants paid to their institution from The European Commission, Gates Foundation, Science for Africa and a NIH K43 Emerging Global Leader Award. AD is a member of the GARD-P Scientific Advisory Committee. ASW reports grants paid to their institution from The Medical Research Council and National Institutes of Health Research. JAB reports grants paid to their institution from EDCTP, Horizon 2020, SNSF, SPHN (SERI), Innosuisse, EDCTP, JPIAMR, Novartis Foundation, and BMGF; consulting fees paid to their institution from Shionogi and GARDP; honoraria for development of educational content paid to their institution from Pfizer, Sandoz, Bayer, and Biomerieux; participating on IDMCs for the Avenir trial, Lakana trial, and Basilea, and the TSC of the CURLY trial; being a Vice President of SwissPedNet; a Working Group leadership role at the Penta Foundation.
