# Supplementary material for: Safety and efficacy of whole-body chlorhexidine gluconate cleansing with or without emollient in hospitalised neonates (NeoCHG): a multicentre, randomised, open-label, factorial pilot trial
Source: eClinicalMedicine. 2024 Feb 25;69:102463. doi: 10.1016/j.eclinm.2024.102463 (PMC10904231; doi:10.1016/j.eclinm.2024.102463)
Supplement: Supplementary information [file mmc1.pdf]

NeoCHG supplementary information

Table of Contents

1. Trial schema 2

2. Skin score assessment criteria 3

3. Trial entry, randomisation and treatment 5

4. Microbiological Laboratory Procedures 6

5. Adapted DAIDS score for neonates 7

6. Priors used in Bayesian ACCEPT analyses 9

7. Additional baseline variables 11

8. Receipt of interventions 12

9. Absolute skin scores over time 16

10. Evidence for interactions between main terms included in models for change in skin score (co-primary outcome) 19

11. Evidence for effects of baseline factors on skin score and effect modification of intervention effects 20

12. ACCEPT analyses of efficacy co-primary outcome (change in log_{10} CFU) 21

13. Evidence for interactions between main terms included in model for total log_{10} CFU (co-primary outcome) 23

14. Evidence for effects of baseline factors on total log_{10} CFU and effect modification of intervention effects 24

15. Sensitivity analyses for total log10 CFU 25

16. Analysis of presence/absence of focal species by Arm 26

17. Resistance overall (for all processed samples) and given growth (for only those samples with specified species detected) 28

18. Analysis of SAEs 32

19. Line listing of SAEs 33

20. ACCEPT analyses of percentage of children with any SAEs 35

1. Trial schema

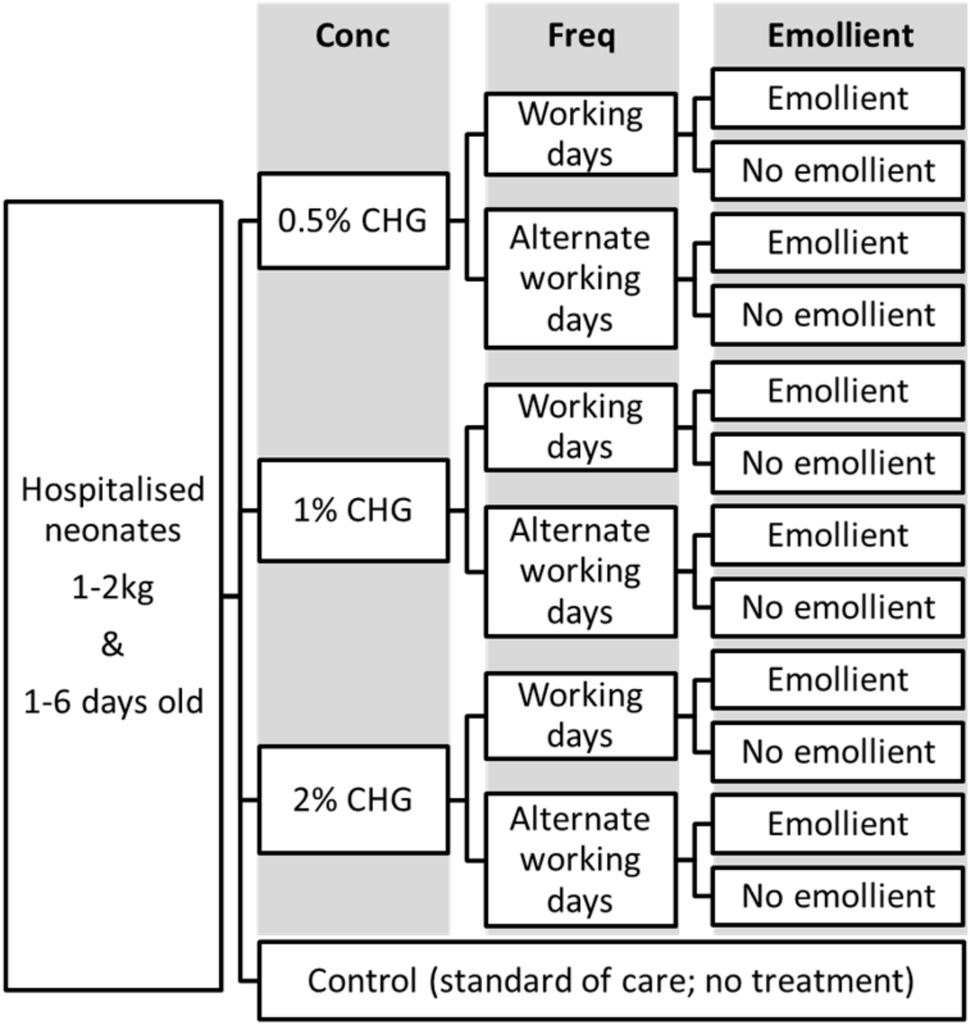

2. Skin score assessment criteria

Skin toxicity score

Adapted from Lund, C. H. & Osborne, J. W. Validity and reliability of the Neonatal Skin Condition score. *JOGNN - J. Obstet. Gynecol. Neonatal Nurs.* **33**, 320–327 (2004).

The skin should be scored on each of the three domains (dryness, erythema and skin breakdown), and then the total score is the sum of the three components (minimum score 0, maximum score 12).

Highest grade from:

Grade 1 = Score of ≥1 in ≥2 categories

Grade 2 = Score of ≥2 in ≥2 categories

Grade 3 = Score of ≥3 in ≥2 categories

Grade 4 = Score of ≥4 in ≥2 categories

Score	Description
**Domain 1**	**Dryness**
0	Normal
1	Dry skin +/- scaling <50%
2	Dry skin + scaling >50% +/- cracking/fissures <50%
3	Very dry + cracking / fissures >50%
4	Very dry + cracking / fissures >75%
**Domain 2**	**Erythema**
0	Normal
1	Erythema <25%
2	Erythema 25-50%
3	Severe Erythema 50-75%
4	Severe Erythema >75%
**Domain 3**	**Skin breakdown**
0	Normal
1	Skin breakdown/ulceration/vesicles <5%
2	Skin breakdown/ulceration/vesicles 5-25%
3	Skin breakdown/ulceration/vesicles 25-50%
4	Skin breakdown/ulceration/vesicles >50%

Figure 3: Skin Affected Area assessment

Modified Lund and Browder chart for estimation of BSA burn involvement in infants and children. (From Harwood-Nuss A, Wolfson A, Linden C. *The clinical practice of emergency medicine*. Philadelphia: Lippincott-Raven; 1996:1207)

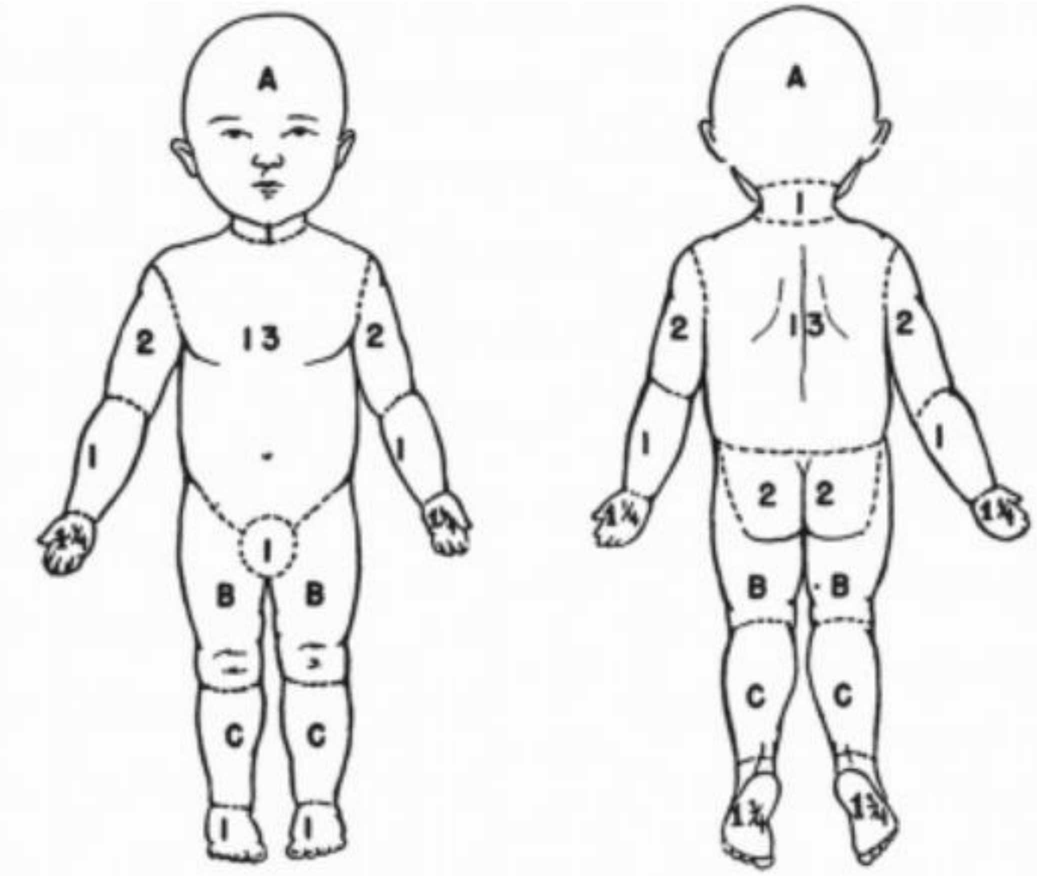

RELATIVE PERCENTAGES OF AREAS AFFECTED BY GROWTH

Area	Age 0	1	5
A = ½ of Head	9½	8½	6½
B = ½ of One Thigh	2¾	3¼	4
C = ½ of One Leg	2½	2½	2¾

% BURN BY AREAS

Probable 3rd° Burn { Head______ Neck______ Body______ Up. Arm______ Forearm______ Hands______
Genitals______ Buttocks______ Thighs______ Legs______ Feet______

Total Burn { Head______ Neck______ Body______ Up. Arm______ Forearm______ Hands______
Genitals______ Buttocks______ Thighs______ Legs______ Feet______

Sum of All Areas__________ Probably 3rd° __________ Total Burn__________

3. Trial entry, randomisation and treatment

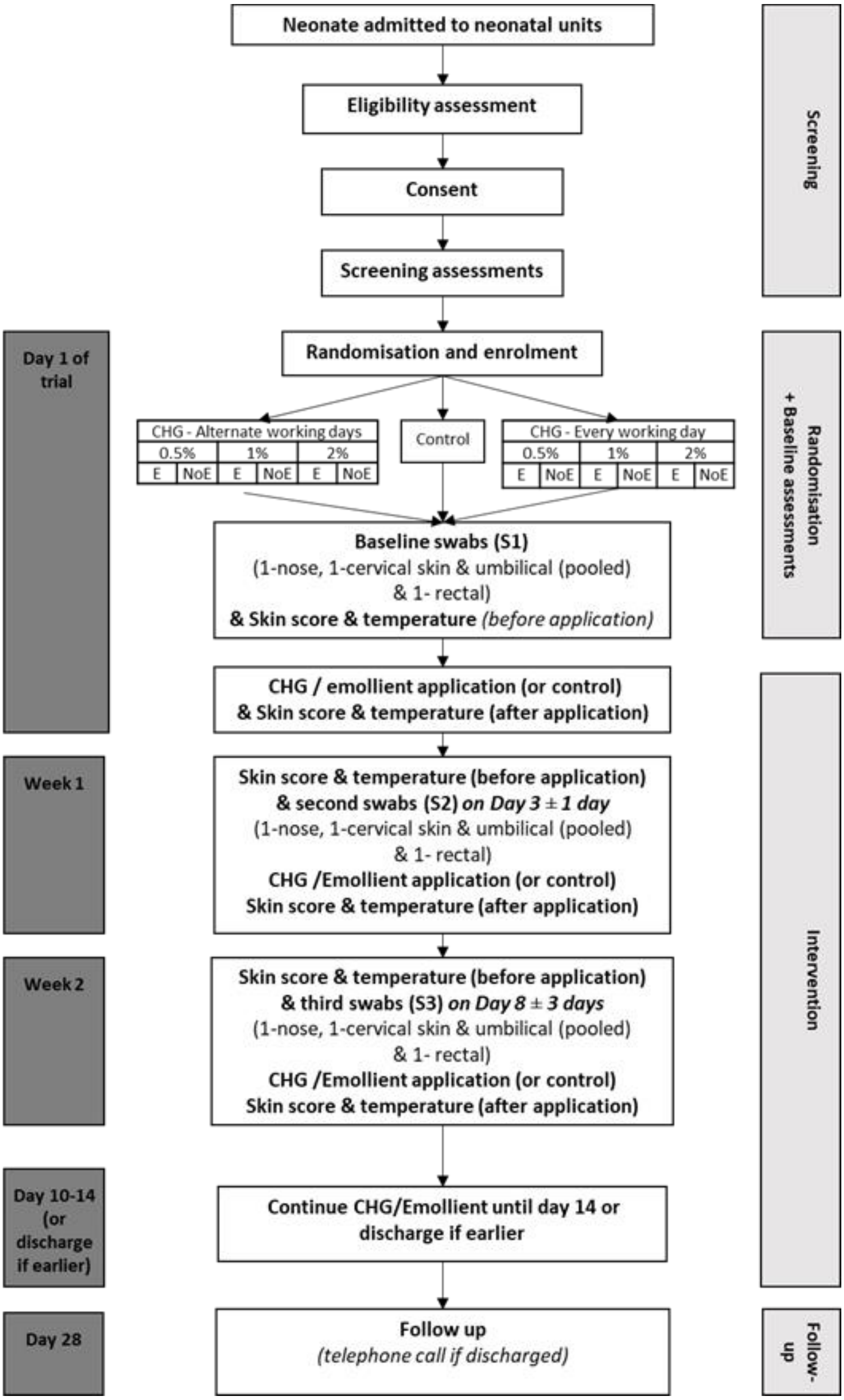

4. Microbiological Laboratory Procedures

Swabs and processing

Swabs in liquid transport medium (LTM) were used. On arrival in the laboratory, the swab in LTM was vortexed, and ten-fold dilutions of the LTM in saline were performed, down to a dilution of 10^{-4}. Twenty microlitre aliquots of neat LTM and of the 10^{-2} and 10^{-4} dilutions were inoculated onto sheep blood agar and McConkey agar. Agar plates were incubated overnight at 35 degrees Celsius aerobically.

Following overnight incubation, the blood agar plate most suitable for colony counts was used to perform a manual colony count. Total colonies, Gram positive colonies and Gram negative colonies were counted. If necessary, the MacConkey agar was used to perform the Gram negative colony count to better differentiate Gram negative bacilli from Gram positive organisms. Colony counts of identified species were also recorded.

The colony counts were converted to log CFU taking into account the dilution factor as well as the volume of LTM initially inoculated, and expressed as log CFU/mL of LTM.

Organism identification

Catalase was used to differentiate Gram positive cocci; catalase positive cocci were identified as *S. aureus* or coagulase negative staphylococci using mannitol salt agar, production of deoxyribonuclease, and presence of staphylococcal protein A, clumping factor and capsular polysaccharides using the Pastorex Staph-Plus Kit (BioRad, Hercules, California, USA). Catalase negative cocci were identified as *Streptococcus pyogenes*, *Streptococcus agalactiae* or *Enterococcus* species based on the type of haemolysis on blood agar, susceptibility to bacitracin, aesculin hydrolysis, CAMP test, and where necessary the Streptex Latex Agglutination Kit (Thermo-Fisher, Waltham, Mass, USA).

Gram negative bacilli were identified using the Vitek 2 Advanced Expert System (BioMerieux, Marcy-l'Etoile, France). Yeasts were identified as *Candida albicans* or non-*C. albicans* based on germ tube production.

Susceptibility testing

Susceptibility testing was performed on *S. aureus* isolates and all Gram negative bacilli using Kirby Bauer disc diffusion, following CLSI guidelines and interpreted using 2020 CLSI criteria. Cefoxitin discs were used to test for *S. aureus* susceptibility to beta-lactamase stable penicillins. Enterobacterales were tested against ampicillin, amoxicillin-clavulanate, piperacillin-tazobactam, cefotaxime or ceftriaxone, ceftazidime, cefepime, ertapenem, meropenem, gentamicin, amikacin and ciprofloxacin. Non-fermenting Gram negative bacilli (*A. baumannii* and *P. aeruginosa*) were tested against piperacillin-tazobactam, cefepime, ceftazidime, ceftriaxone (for *A. baumannii* only), imipenem, meropenem, gentamicin, amikacin, ciprofloxacin and cotrimoxazole (for *A. baumannii* only).

5. Adapted DAIDS score for neonates

Note: reproduced from the IMPAACT study

Parameter	Grade 0 Normal	Grade 1 Mild	Grade 2 Moderate	Grade 3 Severe	Grade 4 Potentially Life-Threatening
Apnea*<br><br>*Apnea spell defined as apnea event >20s or associated with bradycardia, hypoxia or cyanosis	No apneas	< 6 spells* per day	6<12 spells* per day or nasal cannula for apnea	12 or more spells* per day or nasal continuous positive airway pressure (NCPAP) for apnea	Requires intubation for apnea
Anemia	Hb > 10	Hgb 8-10 g/dL	Hgb ≤ 8 g/dL	Requires packed red cell transfusion, no clinical signs	Requires packed red cell transfusion, clinical signs of shock
Congenital Anomalies	None	Minor (no impairment of function)	Minor (no impairment of function), future treatment may be needed	Major (impairment of function), no immediate treatment needed	Major (impairment of function), immediate treatment needed
Congenital Heart Disease	No congenital heart disease	Minor (no impairment of function), no treatment needed	Minor (no impairment of function), future treatment may be needed	Major (impairment of function), no immediate treatment needed	Major (impairment of function), immediate treatment needed
Elecotrolyte/ Metabolic Disorders	None	-------------------	Electrolyte/Metabolic disorder, no systemic signs	------------------------	Electrolyte/Metabolic disorder with systemic signs
GI dysfunction [including necrotizing entercolitis(NEC)]	None	Not ill, abnormal abdominal exam, no treatment needed (e.g., abdominal distension but not NPO)	Mild illness, NPO <3 days (e.g., R/O necrotizing entercolitis (NEC) evaluation, Stage I NEC)	Moderate illness, NPO 3-7 days, or medical treatment (e.g., Stage II NEC)	Severe illness, NPO>7 days, or surgical treatment needed (e.g., Stage III NEC)
Hypertension	No BP performed	Systolic BP> 80-100, no treatment	Systemic BP > 100, no treatment	Treated with one agent	Treatment with multiple agents
Hypotension	No BP performed	Mild clinical signs, no treatment needed	Symptomatic, treated with IV fluids	Symptomatic, treated with single medication	Clinical signs of shock or requiring use of multiple medications
Intraventricular Hemmorhage	No abnormality noted or not assessed	Germinal matrix hemorrhage	Blood in ventricle, no enlargement	Blood in ventricle, with ventricular enlargement	Parenchymal hemorrhage and/or need for ventricular drainage
Jaundice	No jaundice	Mild jaundice, no treatment	Phototherapy and/or IVIG	Exchange transfusion	Acute bilirubin encephalopathy
Neonatal Abstinence Syndrome (NAS)	No history of abstinence syndrome	NAS signs, no medical treatment	NAS controlled with single drug	NAS controlled with two drugs	NAS with seizures
Neurologic compromise	Normal neurological examinations	Mildly abnormal neurologic exam, no clinical or EEG seizure activity	Stage I encephalopathy or possible clinical or EEG seizure activity but no treatment	Stage II encephalopathy or single drug seizure therapy	Stage III encephalopathy or multiple drug seizure therapy
Neutropenia	ANC> 1000/mm3	ANC<1000/mm^3	ANC<500/mm^3	Treated with GCSF	WBC transfusion
Patent Ductus Arteriosus	No murmur	Clinical signs, no treatment	Treatment with fluid restriction or diuretics	Treatment with indomethacin or ibuprofen	Surgical ligation
Persistent Pulmonary Hypertension	No difference in pre- and post-ductal difference	Supplemental O_2 but no mechanical ventilation	Conventional ventilator < 5 days	Conventional ventilation 5-10 days, alternative ventilation (e.g., high frequency oscillatory ventilation	Mechanical ventilation > 10 days

Parameter	Grade 0<br>Normal	Grade 1<br>Mild	Grade 2<br>Moderate	Grade 3<br>Severe	Grade 4<br>Potentially<br>Life-Threatening
				(HFOV), sildenafil and/or nitric oxide	
Renal Dysfunction	Wet diapers documente d	Urine output 1 < 1.5 mL/kg/hr	Urine output 0.5 <1.0 mL/kg/hr	Urine output 0 < 0.5 mL/kg/hr	Prolonged anuria
Respiratory Insufficiency	Baby is in room air – no supplement al oxygen	Nasal cannula oxygen with FiO2 <0.5	Continuous positive airway pressure (CPAP) or nasal cannula with FiO2>0.5	Conventional ventilation	Alternative ventilation (e.g., HFOV)
Retinopathy of Prematurity (ROP)	Normal vascularizati on or not assessed	Incomplete vascularization	Pre-threshold ROP	Threshold ROP or ROP treatment	Retinal detachment
Sepsis	No sepsis symptoms and signs	Septic evaluation, no treatment	R/O sepsis, antibiotics for ≤72 hours	A confirmed blood stream infection with a course of antibiotic treatment, no septic shock or meningitis	A confirmed blood stream infection with a course of antibiotic treatment with septic shock or meningitis
Thrombocytopenia	>100 000	75,000-100,000	50,000< 75,000	25,000<50,000	< 25,000 or platelet transfusion

6. Priors used in Bayesian ACCEPT analyses

Non-informative priors

Non informative priors for both change in skin score and change in CFU were selected to capture a wide range of possible values:

- Intercept mean =4, sd = 50
- Difference mean =0, sd = 20

Informative priors (optimistic and sceptical)

Informative priors were determined based on Dramowski et al NeoCOLONIZE [1] data and expert clinician opinion (Adrie Bekker, Angela Dramwoski and Neal Russell) during an online meeting on 12/10/2021. There was a lack of robust data to support the use of strong priors, especially with inclusion from data from a different setting in Bangladesh. In particular, Johnson et al [2,3] was not considered relevant as no gram negative bacteria were detected in that study, whereas they were expected to be very common in NeoCHG.

Intercept specification

The group receiving 1% CHG applied on weekdays with no emollient was selected as the reference (intercept) as prior data from NeoCOLONIZE could be used to inform this group. Using mean values from the NeoCOLONIZE study and approximations of sd with a multiplier on the sd to capture extra uncertainty, the intercepts were specified as following a normal distribution with parameters:

- Log CFU: mean 3.5; standard deviation 1; multiplier 3
- Skin score: mean 4.6; standard deviation 0.8; multiplier 2

A higher multiplier was chosen for log CFU as it expected to be more variable over time and sites than skin score.

Difference specification

Clinicians specified the mean difference that optimistic and sceptical persons knowledgeable on the topic might assume, versus a reference of 1% CHG applied on weekdays with no emollient. The approach taken was that both optimistic and sceptical persons were assumed to have some belief that the opposing view could be correct, given the assumption of equipoise for the trial to be able to go ahead. The mean values selected were:

Log CFU colony count

Parameter	Optimistic mean	Sceptical mean
0.5% CHG	Higher by 0.5	No effect
2% CHG	Lower by 0.5	No effect
Emollient	0	Higher by 0.5
Alternate day application	Higher by 0.5	No effect
Control	Higher by 1	No effect

Differences were specified to follow a Normal distribution with mean as above and 2.5% of the distribution more extreme than the opposing value (sceptical for optimistic priors and optimistic for sceptical priors). This equates to a Normal distribution with:

- Mean = optimistic or sceptical mean
- sd = abs(sceptical mean – optimistic mean)/1.96

Where 1.96 is the critical value from the t-distribution with infinite degrees of freedom.

Priors were then reformulated to a reference of 0.5%CHG/alternate weekday/no emollient before analysis to match reference specification in focal analysis.

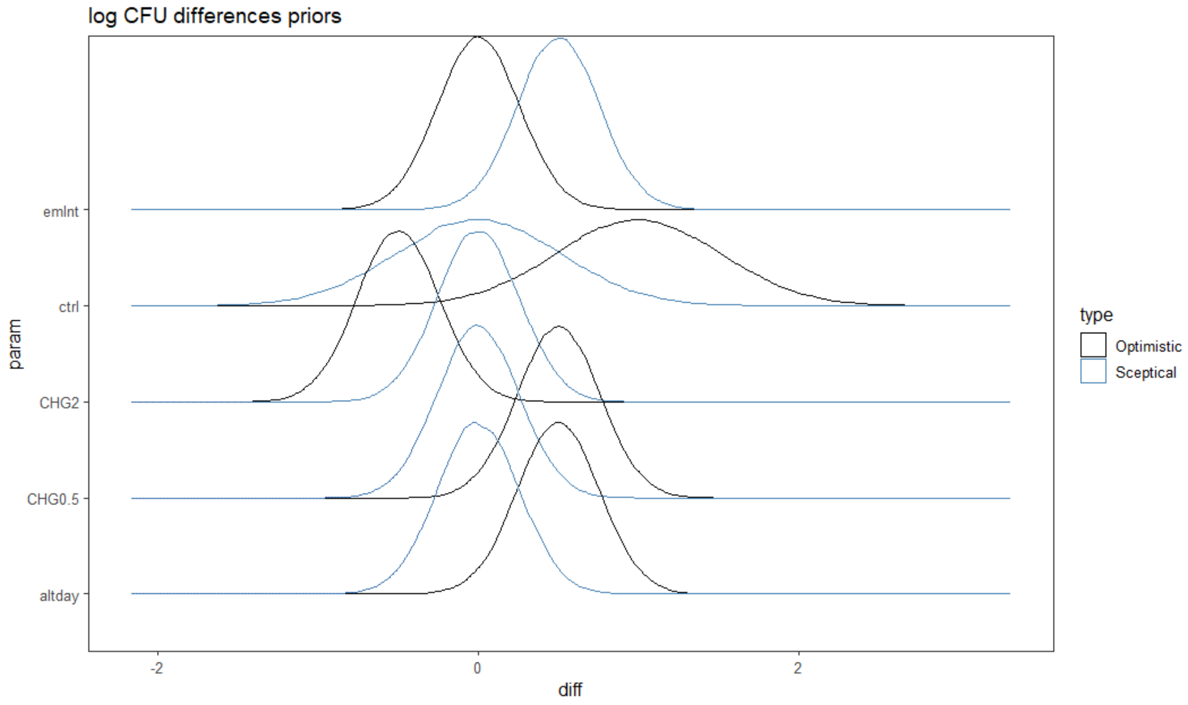

7. Additional baseline variables

Factor	Level	Overall	0.5% CHG (N=64)	1% CHG (N=64)	2% CHG (N=64)	Alternate wd (N=96)	Working days (N=96)	Emollient (N=96)	No emollient (N=96)	Control (N=16)
Method of gestational age determination	Ultrasound	198 (95%)	63 (98%)	62 (97%)	57 (89%)	91 (95%)	91 (95%)	88 (92%)	94 (98%)	16 (100%)
	LMP (last menstrual period)	3 (1%)	1 (2%)	1 (2%)	1 (2%)	3 (3%)		3 (3%)		
	Other	7 (3%)		1 (2%)	6 (9%)	2 (2%)	5 (5%)	5 (5%)	2 (2%)	
Skin score at randomisation	0	188 (90%)	60 (94%)	56 (88%)	60 (94%)	85 (89%)	91 (95%)	86 (90%)	90 (94%)	12 (75%)
	1	20 (10%)	4 (6%)	8 (12%)	4 (6%)	11 (11%)	5 (5%)	10 (10%)	6 (6%)	4 (25%)
Any comorbidity		204 (98%)	63 (98%)	63 (98%)	62 (97%)	95 (99%)	93 (97%)	94 (98%)	94 (98%)	16 (100%)
Hyaline membrane disease/ Respiratory Distress Syndrome		145 (70%)	45 (70%)	45 (70%)	43 (67%)	74 (77%)	59 (61%)	66 (69%)	67 (70%)	12 (75%)
Sepsis		85 (41%)	32 (50%)	21 (33%)	25 (39%)	34 (35%)	44 (46%)	37 (39%)	41 (43%)	7 (44%)
Necrotising enterocolitis		0 (0%)	0 (0%)	0 (0%)	0 (0%)	0 (0%)	0 (0%)	0 (0%)	0 (0%)	0 (0%)
Rupture of membranes before delivery	No/at C-section	91 (44%)	21 (33%)	31 (48%)	31 (48%)	48 (50%)	35 (36%)	46 (48%)	37 (39%)	8 (50%)
	No/not at C-section	6 (3%)		4 (6%)	2 (3%)	3 (3%)	3 (3%)	2 (2%)	4 (4%)	
	Yes/Assisted	16 (8%)	7 (11%)	2 (3%)	7 (11%)	9 (9%)	7 (7%)	9 (9%)	7 (7%)	
	Yes/Spontaneous	95 (46%)	36 (56%)	27 (42%)	24 (38%)	36 (38%)	51 (53%)	39 (41%)	48 (50%)	8 (50%)
Prolonged rupture of membranes (>18h)	Yes	30 (14%)	10 (16%)	9 (14%)	8 (12%)	15 (16%)	12 (12%)	13 (14%)	14 (15%)	3 (19%)
	No	176 (85%)	53 (83%)	54 (84%)	56 (88%)	79 (82%)	84 (88%)	82 (85%)	81 (84%)	13 (81%)
	Unknown	2 (1%)	1 (2%)	1 (2%)		2 (2%)		1 (1%)	1 (1%)	
Liquor clarity	Clear	153 (74%)	52 (81%)	41 (64%)	48 (75%)	60 (62%)	81 (84%)	69 (72%)	72 (75%)	12 (75%)
	Meconium stained	7 (3%)	2 (3%)	1 (2%)	4 (6%)	5 (5%)	2 (2%)	4 (4%)	3 (3%)	
	Unknown	48 (23%)	10 (16%)	22 (34%)	12 (19%)	31 (32%)	13 (14%)	23 (24%)	21 (22%)	4 (25%)
Treatment for suspected sepsis or chorioamnionitis in mother before delivery	Yes	60 (29%)	22 (34%)	17 (27%)	18 (28%)	33 (34%)	24 (25%)	25 (26%)	32 (33%)	3 (19%)
	No	141 (68%)	41 (64%)	45 (70%)	43 (67%)	61 (64%)	68 (71%)	68 (71%)	61 (64%)	12 (75%)
	Unknown	7 (3%)	1 (2%)	2 (3%)	3 (5%)	2 (2%)	4 (4%)	3 (3%)	3 (3%)	1 (6%)

8. Receipt of interventions

Note: CHG=chlorhexidine

(a) randomisation to 0.5% CHG, 1% CHG, 2% CHG or Control

var	level/metric	0.5% CHG (N=64)	1% CHG (N=64)	2% CHG (N=64)	Control (N=16)	pvalue of difference between CHG arms
Total number of CHG applications	median	5	5	6	0	0.50
	(IQR)	(4, 8)	(4, 6)	(4, 8)	(0, 0)	
	N	64	64	64	16	
Last day of CHG application	median	10	10	10		0.58
	(IQR)	(6, 13)	(5, 12)	(6, 12)		
	N	64	64	64		
Number of CHG applications before day 3 swab	median	2	1	1	0	0.74
	(IQR)	(1, 2)	(1, 2)	(1, 2)	(0, 0)	
	N	59	61	62	16	
Number of CHG applications before day 8 swab	median	4	3	3	0	0.91
	(IQR)	(3, 5)	(3, 5)	(3, 5)	(0, 0)	
	N	58	52	57	16	
CHG applications before day 3 swab	0				16 (100%)	0.73
	1	28 (47%)	33 (54%)	33 (53%)		
	2	31 (53%)	28 (46%)	29 (47%)		
CHG applications before day 8 swab	0				16 (100%)	0.87
	2	7 (12%)	9 (17%)	10 (18%)		
	3	22 (38%)	18 (35%)	21 (37%)		
	4	14 (24%)	8 (15%)	10 (18%)		
	5	15 (26%)	17 (33%)	16 (28%)		
Emollient applied after all CHG applications		32 (50%)	32 (50%)	32 (50%)		1.0
Temperature taken before all CHG applications		64 (100%)	64 (100%)	64 (100%)		
Skin score assessed before all CHG applications		64 (100%)	64 (100%)	64 (100%)		
Temperature taken after all CHG applications		64 (100%)	64 (100%)	64 (100%)		
Skin score assessed after all CHG applications		64 (100%)	64 (100%)	64 (100%)		
Temporary treatment interruption		16 (25%)	17 (27%)	16 (25%)		0.97
Temporary interruption reason	Baby too ill	1 (6%)	1 (6%)	2 (12%)		0.99
	Guardian refused		1 (6%)	1 (6%)		
	Hypothermia	1 (6%)	1 (6%)	1 (6%)		
	Hypothermia & Public holiday			1 (6%)		
	Physician decision	1 (6%)				
	Public holiday	13 (81%)	14 (82%)	11 (69%)		
Temporary interrupt N treatments missed	median	1	1	1		0.62
	(IQR)	(1, 1)	(1, 1)	(1, 1)		
	N	16	17	16		
Permanent treatment interruption			3 (5%)	1 (2%)		0.32

Permanent interruption reason	Abandoned Child		1 (33%)			0.32
	Baby too ill		2 (67%)	1 (100%)		
CHG overdose		0 (0%)	0 (0%)	0 (0%)	0 (0%)	n/a
Emollient overdose		0 (0%)	0 (0%)	0 (0%)	0 (0%)	n/a
Use of non-trial emollient		0 (0%)	0 (0%)	0 (0%)	0 (0%)	n/a
Number of skin score assessments	median	6	6	6	6	0.52
	(IQR)	(4, 8)	(4, 7)	(4, 8)	(5, 6)	
	N	64	64	64	16	
Last day of skin score assessment	median	10	10	10	12	0.57
	(IQR)	(6, 13)	(5, 12)	(6, 12)	(11, 12)	
	N	64	64	64	16	
Day 28 follow-up		51 (80%)	53 (83%)	54 (84%)	16 (100%)	0.78

(b) by randomisation to alternate working days or working days (excluding control)

var	level/metric	Alternate wd (N=96)	Working days (N=96)	pvalue
Total number of CHG applications	median	5	8	<0.0001
	(IQR)	(3, 6)	(5, 9)	
	N	96	96	
Last day of CHG application	median	10	10	0.61
	(IQR)	(5, 12)	(5, 12)	
	N	96	96	
Number of CHG applications before day 3 swab	median	1	2	<0.0001
	(IQR)	(1, 1)	(2, 2)	
	N	88	94	
Number of CHG applications before day 8 swab	median	3	5	<0.0001
	(IQR)	(2, 3)	(4, 5)	
	N	79	88	
CHG applications before day 3 swab	1	88 (100%)	6 (6%)	<0.0001
	2		88 (94%)	
CHG applications before day 8 swab	2	25 (32%)	1 (1%)	<0.0001
	3	54 (68%)	7 (8%)	
	4		32 (36%)	
	5		48 (55%)	
Emollient applied after all CHG applications		48 (50%)	48 (50%)	1.0
Temperature taken before all CHG applications		96 (100%)	96 (100%)	n/a
Skin score assessed before all CHG applications		96 (100%)	96 (100%)	n/a
Temperature taken after all CHG applications		96 (100%)	96 (100%)	n/a
Skin score assessed after all CHG applications		96 (100%)	96 (100%)	n/a
Temporary treatment interruption		20 (21%)	29 (30%)	0.19
Temporary interruption reason	Baby too ill	3 (15%)	1 (3%)	0.14
	Guardian refused	1 (5%)	1 (3%)	
	Hypothermia		3 (10%)	

	Hypothermia & Public holiday	1 (5%)		
	Physician decision		1 (3%)	
	Public holiday	15 (75%)	23 (79%)	
Temporary interrupt N treatments missed	median	1	1	0.79
	(IQR)	(1, 1)	(1, 1)	
	N	20	29	
Permanent treatment interruption			4 (4%)	0.13
Permanent interruption reason	Abandoned Child		1 (25%)	0.13
	Baby too ill		3 (75%)	
CHG overdose		0 (0%)	0 (0%)	n/a
Emollient overdose		0 (0%)	0 (0%)	n/a
Use of non-trial emollient		0 (0%)	0 (0%)	n/a
Number of skin score assessments	median	5	8	<0.0001
	(IQR)	(3, 6)	(5, 10)	
	N	96	96	
Last day of skin score assessment	median	10	10	0.62
	(IQR)	(5, 12)	(5, 12)	
	N	96	96	
Day 28 follow-up		75 (78%)	83 (86%)	0.19

(c) by randomisation to emollient or no emollient (excluding control)

**var**	**level/metric**	**Emollient (N=96)**	**No emollient (N=96)**	**pvalue**
Total number of CHG applications	median	5	6	0.53
	(IQR)	(4, 7)	(4, 8)	
	N	96	96	
Last day of CHG application	median	10	10	0.69
	(IQR)	(5, 12)	(6, 12)	
	N	96	96	
Number of CHG applications before day 3 swab	median	1	1	0.88
	(IQR)	(1, 2)	(1, 2)	
	N	92	90	
Number of CHG applications before day 8 swab	median	3	3	0.65
	(IQR)	(3, 5)	(3, 5)	
	N	82	85	
CHG applications before day 3 swab	1	47 (51%)	47 (52%)	1.0
	2	45 (49%)	43 (48%)	
CHG applications before day 8 swab	2	12 (15%)	14 (16%)	0.77
	3	32 (39%)	29 (34%)	
	4	17 (21%)	15 (18%)	
	5	21 (26%)	27 (32%)	
Emollient applied after all CHG applications		96 (100%)		<0.0001

Temperature taken before all CHG applications		96 (100%)	96 (100%)	
Skin score assessed before all CHG applications		96 (100%)	96 (100%)	
Temperature taken after all CHG applications		96 (100%)	96 (100%)	
Skin score assessed after all CHG applications		96 (100%)	96 (100%)	
Temporary treatment interruption		24 (25%)	25 (26%)	1.0
Temporary interruption reason	Baby too ill	4 (17%)		0.34
	Guardian refused	1 (4%)	1 (4%)	
	Hypothermia	1 (4%)	2 (8%)	
	Hypothermia & Public holiday		1 (4%)	
	Physician decision		1 (4%)	
	Public holiday	18 (75%)	20 (80%)	
Temporary interrupt N treatments missed	median	1	1	0.98
	(IQR)	(1, 1)	(1, 1)	
	N	24	25	
Permanent treatment interruption		2 (2%)	2 (2%)	1.0
Permanent interruption reason	Abandoned Child		1 (50%)	1.0
	Baby too ill	2 (100%)	1 (50%)	
CHG overdose		0 (0%)	0 (0%)	
Emollient overdose		0 (0%)	0 (0%)	
Use of non-trial emollient		0 (0%)	0 (0%)	
Number of skin score assessments	median	5	6	0.39
	(IQR)	(4, 7)	(4, 9)	
	N	96	96	
Last day of skin score assessment	median	10	10	0.73
	(IQR)	(5, 12)	(6, 12)	
	N	96	96	
Day 28 follow-up		78 (81%)	80 (83%)	0.85

9. Absolute skin scores over time

Skin score before each CHG application by arm over time

a) By antiseptic arm including control

Day	SkinScore	0.5% CHG	1% CHG	2% CHG	Control
1	0	52 (93%)	51 (88%)	53 (93%)	12 (86%)
1	1	4 (7%)	7 (12%)	4 (7%)	2 (14%)
2	0	36 (90%)	33 (89%)	36 (97%)	3 (75%)
2	1	4 (10%)	4 (11%)	1 (3%)	1 (25%)
3	0	45 (92%)	50 (96%)	47 (87%)	12 (86%)
3	1	4 (8%)	2 (4%)	7 (13%)	2 (14%)
4	0	32 (94%)	29 (88%)	29 (94%)	4 (100%)
4	1	2 (6%)	4 (12%)	2 (6%)	
5	0	28 (93%)	31 (91%)	36 (92%)	8 (89%)
5	1	2 (7%)	3 (9%)	3 (8%)	1 (11%)
6	0	14 (93%)	9 (100%)	10 (91%)	3 (100%)
6	1	1 (7%)		1 (9%)	
7	0	22 (96%)	19 (95%)	19 (90%)	8 (100%)
7	1	1 (4%)	1 (5%)	2 (10%)	
8	0	26 (87%)	28 (97%)	31 (82%)	6 (75%)
8	1	4 (13%)	1 (3%)	7 (18%)	2 (25%)
9	0	26 (90%)	17 (77%)	28 (97%)	5 (83%)
9	1	3 (10%)	5 (23%)	1 (3%)	1 (17%)
10	0	21 (78%)	20 (80%)	23 (79%)	6 (86%)
10	1	6 (22%)	5 (20%)	5 (17%)	1 (14%)
10	2			1 (3%)	
11	0	14 (67%)	11 (73%)	11 (69%)	4 (100%)
11	1	7 (33%)	4 (27%)	5 (31%)	
12	0	7 (64%)	9 (75%)	10 (62%)	6 (86%)
12	1	4 (36%)	3 (25%)	6 (38%)	1 (14%)
13	0	6 (100%)	2 (67%)	2 (67%)	1 (100%)
13	1		1 (33%)	1 (33%)	
14	0	11 (73%)	8 (80%)	8 (80%)	3 (100%)
14	1	4 (27%)	2 (20%)	2 (20%)	

b) By frequency, excluding control

Day	SkinScore	Alternate wd	Working days
1	0	65 (87%)	91 (95%)
1	1	10 (13%)	5 (5%)
2	0	19 (79%)	86 (96%)
2	1	5 (21%)	4 (4%)
3	0	56 (89%)	86 (93%)

3	1	7 (11%)	6 (7%)
4	0	19 (83%)	71 (95%)
4	1	4 (17%)	4 (5%)
5	0	40 (89%)	55 (95%)
5	1	5 (11%)	3 (5%)
6	0	10 (83%)	23 (100%)
6	1	2 (17%)	
7	0	32 (94%)	28 (93%)
7	1	2 (6%)	2 (7%)
8	0	29 (85%)	56 (89%)
8	1	5 (15%)	7 (11%)
9	0	22 (88%)	49 (89%)
9	1	3 (12%)	6 (11%)
10	0	24 (75%)	40 (82%)
10	1	7 (22%)	9 (18%)
10	2	1 (3%)	
11	0	12 (71%)	24 (69%)
11	1	5 (29%)	11 (31%)
12	0	14 (74%)	12 (60%)
12	1	5 (26%)	8 (40%)
13	0	5 (71%)	5 (100%)
13	1	2 (29%)	
14	0	11 (73%)	16 (80%)
14	1	4 (27%)	4 (20%)

c) By emollient, excluding control

Day	SkinScore	Emollient	No emollient
1	0	76 (90%)	80 (92%)
1	1	8 (10%)	7 (8%)
2	0	52 (90%)	53 (95%)
2	1	6 (10%)	3 (5%)
3	0	73 (94%)	69 (90%)
3	1	5 (6%)	8 (10%)
4	0	44 (94%)	46 (90%)
4	1	3 (6%)	5 (10%)
5	0	47 (96%)	48 (89%)
5	1	2 (4%)	6 (11%)
6	0	13 (93%)	20 (95%)
6	1	1 (7%)	1 (5%)
7	0	32 (94%)	28 (93%)
7	1	2 (6%)	2 (7%)
8	0	38 (86%)	47 (89%)
8	1	6 (14%)	6 (11%)

9	0	36 (90%)	35 (88%)
9	1	4 (10%)	5 (12%)
10	0	32 (84%)	32 (74%)
10	1	6 (16%)	10 (23%)
10	2		1 (2%)
11	0	16 (64%)	20 (74%)
11	1	9 (36%)	7 (26%)
12	0	10 (59%)	16 (73%)
12	1	7 (41%)	6 (27%)
13	0	6 (100%)	4 (67%)
13	1		2 (33%)
14	0	14 (78%)	13 (76%)
14	1	4 (22%)	4 (24%)

10. Evidence for interactions between main terms included in models for change in skin score (co-primary outcome)

**Effect 1**	**Effect 2**	**p-value of interaction**
concentration	frequency	0.87
concentration	emollient	0.73
frequency	emollient	0.35
concentration	day	0.76
emollient	day	0.45
**frequency**	**day**	**0.042**

11. Evidence for effects of baseline factors on skin score and effect modification of intervention effects

Variable	Effect/ Interaction	p-value of effect/ interaction
Intrapartum antibiotics	Main effect	0.0065
	Concentration interaction	0.89
	Frequency interaction	0.23
	**Emollient interaction**	**0.026**
Postpartum antibiotics at baseline	Main effect	0.16
	Concentration interaction	0.71
	Frequency interaction	0.77
	Emollient interaction	0.34
Rupture of membranes	Main effect	0.46
	Concentration interaction	0.79
	Frequency interaction	0.25
	Emollient interaction	0.29
Very low birth weight (<1500g)	Main effect	0.29
	Concentration interaction	0.43
	Frequency interaction	0.14
	**Emollient interaction**	**0.019**
Age at baseline (two groups split at median)	Main effect	0.39
	Concentration interaction	0.14
	Frequency interaction	0.28
	Emollient interaction	0.25

12. ACCEPT analyses of efficacy co-primary outcome (change in log_{10} CFU)

Factor	Acceptability Threshold	Acceptability values		
		Non-informative	Optimistic	Sceptical
1% CHG	-2	0%	0%	0%
	-1	5%	0%	0%
	-0.5	41%	13%	2%
	0	88%	93%	70%
	0.5	100%	100%	100%
	1	100%	100%	100%
	2	100%	100%	100%
2% CHG	-2	0%	0%	0%
	-1	0%	1%	0%
	-0.5	7%	30%	2%
	0	49%	94%	49%
	0.5	93%	100%	98%
	1	100%	100%	100%
	2	100%	100%	100%
Working days	-2	0%	0%	0%
	-1	2%	0%	0%
	-0.5	36%	31%	4%
	0	92%	98%	83%
	0.5	100%	100%	100%
	1	100%	100%	100%
	2	100%	100%	100%
Emollient	-2	0%	0%	0%
	-1	0%	0%	0%
	-0.5	0%	0%	0%
	0	10%	12%	1%
	0.5	68%	93%	62%
	1	99%	100%	100%
	2	100%	100%	100%
Control	-2	0%	0%	0%
	-1	9%	0%	0%
	-0.5	32%	2%	8%
	0	64%	22%	47%
	0.5	88%	72%	90%
	1	98%	97%	99%
	2	100%	100%	100%

Note: Acceptability values are the probability that the treatment effect compared to 0.5% CHG/alternate working days/no emollient is more negative (ie better) than the acceptability threshold under different priors. E.g Probability 1% CHG causes less logCFU than 0.5% CHG (i.e. acceptability threshold of 0) is 88%, 93% and 70% under non-informative, optimistic and sceptical priors respectively; probability of reduction being at least 5 log10 CFU (i.e. acceptability threshold of -0.5) is 41%, 13% and 2%, under the different prior specifications.

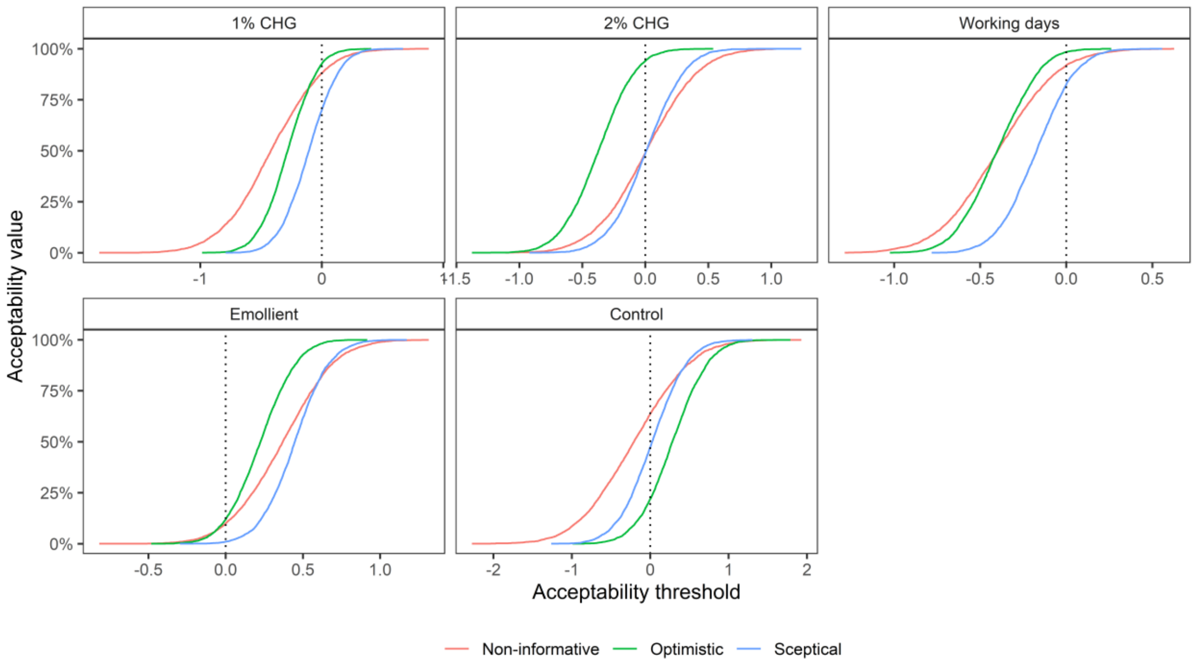

Note: 0 acceptability threshold corresponds to the 0.5% CHG/alternate working days/no emollient reference group.

13. Evidence for interactions between main terms included in model for total log_{10} CFU (co-primary outcome)

Effect 1	Effect 2	p-value of interaction
concentration	Baseline log10 CFU	0.65
frequency	Baseline log10 CFU	0.57
emollient	Baseline log10 CFU	0.53
concentration	frequency	0.66
concentration	emollient	0.42
frequency	emollient	0.72
concentration	day	0.97
emollient	day	0.97
frequency	day	0.49

14. Evidence for effects of baseline factors on total log_{10} CFU and effect modification of intervention effects

Variable	Effect/ Interaction (outcome: total log_{10} CFU)	p-value of effect/ interaction
Intrapartum antibiotics	Main effect	0.98
	Concentration interaction	0.27
	Frequency interaction	0.80
	Emollient interaction	0.82
Postpartum antibiotics at baseline	Main effect	0.19
	Concentration interaction	0.093
	**Frequency interaction**	**0.050**
	Emollient interaction	0.19
Rupture of membranes	Main effect	0.32
	Concentration interaction	0.44
	**Frequency interaction**	**0.02**
	Emollient interaction	0.81
Very low birth weight (<1500g)	Main effect	0.18
	Concentration interaction	0.20
	Frequency interaction	0.32
	Emollient interaction	0.17
Age at baseline (two groups split at median)	Main effect	0.30
	Concentration interaction	0.43
	Frequency interaction	0.16
	Emollient interaction	0.32
Mode of delivery (grouped to C-section vs vaginal delivery)*	Main effect	0.58
	Concentration interaction	0.59
	Frequency interaction	0.82
	Emollient interaction	0.75

Note: p-values test the effect of baseline factors that were pre-specified unless otherwise noted below; interaction terms added to the model adjusting for the baseline factor one at a time.

* post-hoc analyses requested by the Data Monitoring Committee.

15.Sensitivity analyses for total log10 CFU

All analyses are as per the primary analysis either restricted to one sample only (and day of sampling removed from the model) or with additional factors added. Estimates are versus the **0.5% CHG, alternate days with no emollient** reference group from a normal linear regression, adjusted for arm, site (Bangladesh and South Africa), and day of sample where required.

REPEATED SAMPLES SENSITIVITY ANLYSIS

First sample over 48h from baseline

Effect	Estimate	lower 95% CI	upper 95% CI	p-value	p-value of factor
1% CHG	-1.2	-2.2	-0.2	0.019	0.050
2% CHG	-0.7	-1.6	0.1	0.096	
Working days	-0.1	-0.8	0.7	0.88	
Emollient	0.2	-0.6	0.9	0.67	
Control	-0.6	-2.4	1.1	0.48	

Last sample

Effect	Estimate	lower 95% CI	upper 95% CI	p-value	p-value of factor
1% CHG	-0.8	-1.6	0.05	0.064	0.17
2% CHG	-0.2	-0.9	0.5	0.58	
Working days	-0.5	-1.1	0.2	0.16	
Emollient	0.2	-0.4	0.8	0.55	
Control	-0.4	-1.8	1.0	0.57	

DAY OF SAMPLING ANALYSIS (fit days since last CHG application, excluding controls)

Effect	Estimate	lower 95% CI	upper 95% CI	p-value	p-value of factor
1% CHG	-0.4	-1.1	0.3	0.22	0.41
2% CHG	-0.01	-0.6	0.6	0.97	
Working days	-0.3	-0.8	0.3	0.31	
Emollient	0.4	-0.2	0.9	0.17	
Days Since Last CHG	0.2	-0.1	0.6	0.21	

ADJUST FOR IV ANTIBIOTICS in the last 24h before swab

IV antibiotics in last 24h at swab/not includes post-randomisation data and should be interpreted with caution

Effect	Estimate	lower 95% CI	upper 95% CI	p-value	p-value of factor
1% CHG	-0.4	-1.1	0.2	0.18	0.27
2% CHG	0.06	-0.5	0.6	0.85	
Working days	-0.4	-0.9	0.1	0.15	
Emollient	0.2	-0.3	0.7	0.37	
Antibiotics in last 24h	-2.4	-3.2	-1.6	<0.0001	

16. Analysis of presence/absence of focal species by Arm

Note: effect estimates are log odds ratios versus the 0.5% CHG/alternate working days/no emollient reference group, from models adjusted for Trial Arm, Site (Bangladesh & South Africa and day of swab (3 & 8).

Enterobacteriaceae

Effect	Estimate	lower 95% CI	upper 95% CI	p-value	p-value of factor
1% CHG	0.17	-0.48	0.81	0.62	0.78
2% CHG	0.20	-0.39	0.79	0.51	
Working days	-0.39	-0.89	0.11	0.13	
Emollient	0.09	-0.42	0.60	0.74	
Control	-0.48	-1.48	0.52	0.35	

Acinetobacter

Effect	Estimate	lower 95% CI	upper 95% CI	p-value	p-value of factor
1% CHG	-0.07	-1.09	0.95	0.89	0.47
2% CHG	-0.63	-1.76	0.50	0.27	
Working days	0.00	-0.861	0.86	1.00	
Emollient	0.32	-0.553	1.19	0.48	
Control	-0.91	-2.69	0.87	0.32	

Staphycoccus Aureus

Effect	Estimate	lower 95% CI	upper 95% CI	p-value	p-value of factor
1% CHG	-0.61	-2.80	1.59	0.59	0.86
2% CHG	-0.24	-2.20	1.72	0.81	
Working days	-0.91	-2.68	0.86	0.31	
Emollient	0.39	-1.28	2.06	0.64	
Control	-0.40	-4.10	3.30	0.83	

Enterococcus

Effect	Estimate	lower 95% CI	upper 95% CI	p-value	p-value of factor
1% CHG	-0.05	-0.86	0.76	0.90	0.76
2% CHG	0.21	-0.57	1.00	0.60	
Working days	-0.06	-0.70	0.56	0.85	
Emollient	-0.23	-0.86	0.40	0.47	
Control	-0.50	-1.60	0.60	0.37	

Candida (all Yeasts identified were Candida spp)

Effect	Estimate	lower 95% CI	upper 95% CI	p-value	p-value of factor
1% CHG	0.39	-0.94	1.71	0.57	0.72

2% CHG	0.55	-0.78	1.88	0.42	
Working days	0.83	-0.22	1.88	0.12	
Emollient	1.44	0.29	2.58	0.014	

Pseudomonas

Effect	Estimate	lower 95% CI	upper 95% CI	p-value	p-value of factor
1% CHG	0.22	-2.11	2.56	0.85	0.71
2% CHG	-1.32	-5.17	2.53	0.50	
Working days	2.27	-0.30	4.83	0.083	
Emollient	-1.48	-4.16	1.2	0.28	

17. Resistance overall (for all processed samples) and given growth (for only those samples with specified species detected)

Species	Drug	Overall: Day 1	Overall: Day 3	Overall: Day 8	Given Growth: Day 1	Given Growth: Day 3	Given Growth: Day 8
Acinetobacter spp.	Amikacin	18/208 (9%)	24/198 (12%)	25/183 (14%)	18/31 (58%)	24/49 (49%)	25/37 (68%)
Acinetobacter spp.	Cefepime	18/208 (9%)	24/198 (12%)	25/183 (14%)	18/31 (58%)	24/49 (49%)	25/37 (68%)
Acinetobacter spp.	Ceftazidime	19/208 (9%)	26/198 (13%)	27/183 (15%)	19/31 (61%)	26/49 (53%)	27/37 (73%)
Acinetobacter spp.	Ceftriaxone	20/208 (10%)	29/198 (15%)	28/183 (15%)	20/31 (65%)	29/49 (59%)	28/37 (76%)
Acinetobacter spp.	Ciprofloxacin	17/208 (8%)	22/198 (11%)	25/183 (14%)	17/31 (55%)	22/49 (45%)	25/37 (68%)
Acinetobacter spp.	Cotrimoxazole	12/208 (6%)	18/198 (9%)	17/183 (9%)	12/31 (39%)	18/49 (37%)	17/37 (46%)
Acinetobacter spp.	Gentamicin	18/208 (9%)	27/198 (14%)	27/183 (15%)	18/31 (58%)	27/49 (55%)	27/37 (73%)
Acinetobacter spp.	Imipenem	16/208 (8%)	22/198 (11%)	23/183 (13%)	16/31 (52%)	22/49 (45%)	23/37 (62%)
Acinetobacter spp.	Meropenem	17/208 (8%)	23/198 (12%)	23/183 (13%)	17/31 (55%)	23/49 (47%)	23/37 (62%)
Acinetobacter spp.	Piperacillin-tazobactam	19/208 (9%)	22/198 (11%)	25/183 (14%)	19/31 (61%)	22/49 (45%)	25/37 (68%)
E. coli	Amikacin	1/208 (0%)	1/198 (1%)	7/183 (4%)	1/17 (6%)	1/23 (4%)	7/49 (14%)
E. coli	Amoxicillin-clavulanate	3/208 (1%)	6/198 (3%)	14/183 (8%)	3/17 (18%)	6/23 (26%)	14/49 (29%)
E. coli	Ampicillin /amoxicillin	11/208 (5%)	19/198 (10%)	36/183 (20%)	11/17 (65%)	19/23 (83%)	36/49 (73%)
E. coli	Cefepime	3/208 (1%)	6/198 (3%)	19/183 (10%)	3/17 (18%)	6/23 (26%)	19/49 (39%)
E. coli	Cefotaxime/Ceftriaxone	3/208 (1%)	7/198 (4%)	21/183 (11%)	3/17 (18%)	7/23 (30%)	21/49 (43%)
E. coli	Ceftazidime	3/208 (1%)	7/198 (4%)	20/183 (11%)	3/17 (18%)	7/23 (30%)	20/49 (41%)
E. coli	Ciprofloxacin	3/208 (1%)	6/198 (3%)	17/183 (9%)	3/17 (18%)	6/23 (26%)	17/49 (35%)
E. coli	Ertapenem	1/208 (0%)	2/198 (1%)	8/183 (4%)	1/17 (6%)	2/23 (9%)	8/49 (16%)
E. coli	Gentamicin	0/208 (0%)	2/198 (1%)	10/183 (5%)	0/17 (0%)	2/23 (9%)	10/49 (20%)
E. coli	Meropenem	0/208 (0%)	0/198 (0%)	6/183 (3%)	0/17 (0%)	0/23 (0%)	6/49 (12%)
E. coli	Piperacillin-tazobactam	2/208 (1%)	5/198 (3%)	12/183 (7%)	2/17 (12%)	5/23 (22%)	12/49 (24%)
Enterobacter spp.	Amikacin	1/208 (0%)	2/198 (1%)	3/183 (2%)	1/10 (10%)	2/21 (10%)	3/45 (7%)
Enterobacter spp.	Amoxicillin-clavulanate	8/208 (4%)	19/198 (10%)	36/183 (20%)	8/10 (80%)	19/21 (90%)	36/45 (80%)
Enterobacter spp.	Ampicillin /amoxicillin	9/208 (4%)	19/198 (10%)	34/183 (19%)	9/10 (90%)	19/21 (90%)	34/45 (76%)

Species	Drug	Overall: Day 1	Overall: Day 3	Overall: Day 8	Given Growth: Day 1	Given Growth: Day 3	Given Growth: Day 8
Enterobacter spp.	Cefepime	1/208 (0%)	3/198 (2%)	4/183 (2%)	1/10 (10%)	3/21 (14%)	4/45 (9%)
Enterobacter spp.	Cefotaxime/Ceftriaxone	3/208 (1%)	8/198 (4%)	6/183 (3%)	3/10 (30%)	8/21 (38%)	6/45 (13%)
Enterobacter spp.	Ceftazidime	2/208 (1%)	8/198 (4%)	6/183 (3%)	2/10 (20%)	8/21 (38%)	6/45 (13%)
Enterobacter spp.	Ciprofloxacin	3/208 (1%)	3/198 (2%)	4/183 (2%)	3/10 (30%)	3/21 (14%)	4/45 (9%)
Enterobacter spp.	Ertapenem	0/208 (0%)	2/198 (1%)	3/183 (2%)	0/10 (0%)	2/21 (10%)	3/45 (7%)
Enterobacter spp.	Gentamicin	3/208 (1%)	5/198 (3%)	5/183 (3%)	3/10 (30%)	5/21 (24%)	5/45 (11%)
Enterobacter spp.	Meropenem	0/208 (0%)	1/198 (1%)	3/183 (2%)	0/10 (0%)	1/21 (5%)	3/45 (7%)
Enterobacter spp.	Piperacillin-tazobactam	1/208 (0%)	2/198 (1%)	3/183 (2%)	1/10 (10%)	2/21 (10%)	3/45 (7%)
Klebsiella spp.	Amikacin	9/208 (4%)	15/198 (8%)	19/183 (10%)	9/32 (28%)	15/68 (22%)	19/129 (15%)
Klebsiella spp.	Amoxicillin-clavulanate	14/208 (7%)	25/198 (13%)	31/183 (17%)	14/32 (44%)	25/68 (37%)	31/129 (24%)
Klebsiella spp.	Ampicillin /amoxicillin	32/208 (15%)	66/198 (33%)	128/183 (70%)	32/32 (100%)	66/68 (97%)	128/129 (99%)
Klebsiella spp.	Cefepime	22/208 (11%)	47/198 (24%)	67/183 (37%)	22/32 (69%)	47/68 (69%)	67/129 (52%)
Klebsiella spp.	Cefotaxime/Ceftriaxone	22/208 (11%)	47/198 (24%)	72/183 (39%)	22/32 (69%)	47/68 (69%)	72/129 (56%)
Klebsiella spp.	Ceftazidime	22/208 (11%)	47/198 (24%)	72/183 (39%)	22/32 (69%)	47/68 (69%)	72/129 (56%)
Klebsiella spp.	Ciprofloxacin	11/208 (5%)	23/198 (12%)	33/183 (18%)	11/32 (34%)	23/68 (34%)	33/129 (26%)
Klebsiella spp.	Ertapenem	9/208 (4%)	15/198 (8%)	19/183 (10%)	9/32 (28%)	15/68 (22%)	19/129 (15%)
Klebsiella spp.	Gentamicin	20/208 (10%)	43/198 (22%)	69/183 (38%)	20/32 (62%)	43/68 (63%)	69/129 (53%)
Klebsiella spp.	Meropenem	8/208 (4%)	13/198 (7%)	17/183 (9%)	8/32 (25%)	13/68 (19%)	17/129 (13%)
Klebsiella spp.	Piperacillin-tazobactam	9/208 (4%)	19/198 (10%)	20/183 (11%)	9/32 (28%)	19/68 (28%)	20/129 (16%)
Other Enterobacter	Amikacin	4/208 (2%)	3/198 (2%)	6/183 (3%)	4/14 (29%)	3/15 (20%)	6/37 (16%)
Other Enterobacter	Amoxicillin-clavulanate	11/208 (5%)	14/198 (7%)	24/183 (13%)	11/14 (79%)	14/15 (93%)	24/37 (65%)
Other Enterobacter	Ampicillin /amoxicillin	13/208 (6%)	12/198 (6%)	32/183 (17%)	13/14 (93%)	12/15 (80%)	32/37 (86%)
Other Enterobacter	Cefepime	7/208 (3%)	4/198 (2%)	9/183 (5%)	7/14 (50%)	4/15 (27%)	9/37 (24%)
Other Enterobacter	Cefotaxime/Ceftriaxone	9/208 (4%)	5/198 (3%)	11/183 (6%)	9/14 (64%)	5/15 (33%)	11/37 (30%)
Other Enterobacter	Ceftazidime	7/208 (3%)	5/198 (3%)	10/183 (5%)	7/14 (50%)	5/15 (33%)	10/37 (27%)
Other Enterobacter	Ciprofloxacin	6/208 (3%)	3/198 (2%)	10/183 (5%)	6/14 (43%)	3/15 (20%)	10/37 (27%)

Species	Drug	Overall: Day 1	Overall: Day 3	Overall: Day 8	Given Growth: Day 1	Given Growth: Day 3	Given Growth: Day 8
Other Enterobacter	Ertapenem	4/208 (2%)	3/198 (2%)	6/183 (3%)	4/14 (29%)	3/15 (20%)	6/37 (16%)
Other Enterobacter	Gentamicin	8/208 (4%)	3/198 (2%)	7/183 (4%)	8/14 (57%)	3/15 (20%)	7/37 (19%)
Other Enterobacter	Meropenem	2/208 (1%)	3/198 (2%)	6/183 (3%)	2/14 (14%)	3/15 (20%)	6/37 (16%)
Other Enterobacter	Piperacillin-tazobactam	5/208 (2%)	3/198 (2%)	6/183 (3%)	5/14 (36%)	3/15 (20%)	6/37 (16%)
Proteus spp.	Amikacin	0/208 (0%)	0/198 (0%)	0/183 (0%)	0/3 (0%)	0/7 (0%)	0/28 (0%)
Proteus spp.	Amoxicillin-clavulanate	0/208 (0%)	0/198 (0%)	0/183 (0%)	0/3 (0%)	0/7 (0%)	0/28 (0%)
Proteus spp.	Ampicillin /amoxicillin	2/208 (1%)	6/198 (3%)	14/183 (8%)	2/3 (67%)	6/7 (86%)	14/28 (50%)
Proteus spp.	Cefepime	0/208 (0%)	0/198 (0%)	0/183 (0%)	0/3 (0%)	0/7 (0%)	0/28 (0%)
Proteus spp.	Cefotaxime/Ceftriaxone	0/208 (0%)	0/198 (0%)	0/183 (0%)	0/3 (0%)	0/7 (0%)	0/28 (0%)
Proteus spp.	Ceftazidime	0/208 (0%)	0/198 (0%)	0/183 (0%)	0/3 (0%)	0/7 (0%)	0/28 (0%)
Proteus spp.	Ciprofloxacin	0/208 (0%)	0/198 (0%)	0/183 (0%)	0/3 (0%)	0/7 (0%)	0/28 (0%)
Proteus spp.	Ertapenem	0/208 (0%)	0/198 (0%)	0/183 (0%)	0/3 (0%)	0/7 (0%)	0/28 (0%)
Proteus spp.	Gentamicin	0/208 (0%)	0/198 (0%)	1/183 (1%)	0/3 (0%)	0/7 (0%)	1/28 (4%)
Proteus spp.	Meropenem	0/208 (0%)	0/198 (0%)	0/183 (0%)	0/3 (0%)	0/7 (0%)	0/28 (0%)
Proteus spp.	Piperacillin-tazobactam	0/208 (0%)	0/198 (0%)	0/183 (0%)	0/3 (0%)	0/7 (0%)	0/28 (0%)
Pseudomonas spp.	Amikacin	1/208 (0%)	1/198 (1%)	3/183 (2%)	1/7 (14%)	1/2 (50%)	3/3 (100%)
Pseudomonas spp.	Cefepime	0/208 (0%)	1/198 (1%)	2/183 (1%)	0/7 (0%)	1/2 (50%)	2/3 (67%)
Pseudomonas spp.	Ceftazidime	0/208 (0%)	1/198 (1%)	2/183 (1%)	0/7 (0%)	1/2 (50%)	2/3 (67%)
Pseudomonas spp.	Ceftriaxone	1/208 (0%)	1/198 (1%)	2/183 (1%)	1/7 (14%)	1/2 (50%)	2/3 (67%)
Pseudomonas spp.	Ciprofloxacin	1/208 (0%)	1/198 (1%)	3/183 (2%)	1/7 (14%)	1/2 (50%)	3/3 (100%)
Pseudomonas spp.	Cotrimoxazole	7/208 (3%)	1/198 (1%)	3/183 (2%)	7/7 (100%)	1/2 (50%)	3/3 (100%)
Pseudomonas spp.	Gentamicin	1/208 (0%)	1/198 (1%)	3/183 (2%)	1/7 (14%)	1/2 (50%)	3/3 (100%)
Pseudomonas spp.	Imipenem	0/208 (0%)	1/198 (1%)	3/183 (2%)	0/7 (0%)	1/2 (50%)	3/3 (100%)
Pseudomonas spp.	Meropenem	0/208 (0%)	1/198 (1%)	3/183 (2%)	0/7 (0%)	1/2 (50%)	3/3 (100%)
Pseudomonas spp.	Piperacillin-tazobactam	0/208 (0%)	1/198 (1%)	2/183 (1%)	0/7 (0%)	1/2 (50%)	2/3 (67%)
Serratia spp.	Amikacin	2/208 (1%)	3/198 (2%)	1/183 (1%)	2/21 (10%)	3/39 (8%)	1/79 (1%)

Species	Drug	Overall: Day 1	Overall: Day 3	Overall: Day 8	Given Growth: Day 1	Given Growth: Day 3	Given Growth: Day 8
Serratia spp.	Amoxicillin-clavulanate	19/208 (9%)	35/198 (18%)	77/183 (42%)	19/21 (90%)	35/39 (90%)	77/79 (97%)
Serratia spp.	Ampicillin /amoxicillin	21/208 (10%)	36/198 (18%)	69/183 (38%)	21/21 (100%)	36/39 (92%)	69/79 (87%)
Serratia spp.	Cefepime	2/208 (1%)	2/198 (1%)	2/183 (1%)	2/21 (10%)	2/39 (5%)	2/79 (3%)
Serratia spp.	Cefotaxime/Ceftriaxone	2/208 (1%)	2/198 (1%)	4/183 (2%)	2/21 (10%)	2/39 (5%)	4/79 (5%)
Serratia spp.	Ceftazidime	2/208 (1%)	2/198 (1%)	2/183 (1%)	2/21 (10%)	2/39 (5%)	2/79 (3%)
Serratia spp.	Ciprofloxacin	2/208 (1%)	3/198 (2%)	3/183 (2%)	2/21 (10%)	3/39 (8%)	3/79 (4%)
Serratia spp.	Ertapenem	0/208 (0%)	0/198 (0%)	0/183 (0%)	0/21 (0%)	0/39 (0%)	0/79 (0%)
Serratia spp.	Gentamicin	2/208 (1%)	3/198 (2%)	3/183 (2%)	2/21 (10%)	3/39 (8%)	3/79 (4%)
Serratia spp.	Meropenem	0/208 (0%)	0/198 (0%)	0/183 (0%)	0/21 (0%)	0/39 (0%)	0/79 (0%)
Serratia spp.	Piperacillin-tazobactam	2/208 (1%)	0/198 (0%)	1/183 (1%)	2/21 (10%)	0/39 (0%)	1/79 (1%)
Staph aureus	Cefoxitin	3/208 (1%)	4/198 (2%)	2/183 (1%)	3/15 (20%)	4/17 (24%)	2/21 (10%)
Staph aureus	Oxacillin	3/208 (1%)	4/198 (2%)	3/183 (2%)	3/15 (20%)	4/17 (24%)	3/21 (14%)

18.Analysis of SAEs

Table shows number of children with event (percentage of children with event) total number of events. P-values are from exact logistic models. Wd= working days

var	Overall SAEs	p-value	Death	p-value	Life-threatening	p-value	Hospitalisation/ prolonged hosp	Other
Overall	21 (10%) 22		12 (6%) 12		7 (3%) 7		2 (1%) 2	1 (0%) 1
0.5% CHG	4 (6%) 4		4 (6%) 4		0 (0%) 0		0 (0%) 0	0 (0%) 0
1% CHG	8 (12%) 8	0.37	5 (8%) 5	1.0	3 (5%) 3	0.24	0 (0%) 0	0 (0%) 0
2% CHG	8 (12%) 9	0.37	3 (5%) 3	1.0	4 (6%) 4	0.12	1 (2%) 1	1 (2%) 1
Alternate wd	11 (11%) 11		7 (7%) 7		3 (3%) 3		1 (1%) 1	0 (0%) 0
Working days	9 (9%) 10	0.81	5 (5%) 5	0.77	4 (4%) 4	1.0	0 (0%) 0	1 (1%) 1
No emollient	12 (12%) 12		7 (7%) 7		4 (4%) 4		1 (1%) 1	0 (0%) 0
Emollient	8 (8%) 9	0.48	5 (5%) 5	0.77	3 (3%) 3	1.0	0 (0%) 0	1 (1%) 1
Control	1 (6%) 1	1	0 (0%) 0	0.63	0 (0%) 0	n/a	1 (6%) 1	0 (0%) 0

19.Line listing of SAEs

Site	Age At Enrol (days)	Trial Arm	SAE type	SOC_PT	Skin Score Day0	Skin Score Day3	Skin Score Day8
BD	5	1% CHG alternate working days with emollient	Death	Infections and infestations / Sepsis neonatal	0	0	
BD	7	2% CHG each working day with emollient	Death	Infections and infestations / Sepsis neonatal	0	0	0
BD	2	0.5% CHG alternate working days without emollient	Death	Congenital, familial and genetic disorders / Heart disease congenital	0		
BD	3	0.5% CHG alternate working days without emollient	Death	Respiratory, thoracic and mediastinal disorders / Infantile apnoea	0		
BD	3	1% CHG each working day with emollient	Death	Infections and infestations / Sepsis neonatal	0	0	
BD	4	1% CHG alternate working days with emollient	Death	Infections and infestations / Sepsis neonatal	0	0	
BD	2	2% CHG alternate working days with emollient	Death	Infections and infestations / Sepsis neonatal	0	0	0
BD	2	2% CHG alternate working days without emollient	Death	Infections and infestations / Sepsis neonatal	0	0	0
BD	3	0.5% CHG each working day without emollient	Death	Respiratory, thoracic and mediastinal disorders / Infantile apnoea	0	0	0
BD	5	1% CHG each working day without emollient	Death	Infections and infestations / Sepsis neonatal	0	0	0
BD	3	1% CHG alternate working days without emollient	Death	General disorders and administration site conditions / Sudden death	0	0	0
ZA	4	0.5% CHG each working day without emollient	Death	Gastrointestinal disorders / Necrotising colitis	0	0	0
BD	7	2% CHG alternate working days without emollient	Life-threatening	Congenital, familial and genetic disorders / Heart disease congenital	0	0	0
BD	5	1% CHG each working day without emollient	Life-threatening	Infections and infestations / Sepsis neonatal	0	0	0
ZA	3	2% CHG alternate working days with emollient	Life-threatening	Congenital, familial and genetic disorders / Congenital small intestinal atresia	0	0	0
ZA	3	1% CHG alternate working days without emollient	Life-threatening	Gastrointestinal disorders / Necrotising colitis	0	1	0
ZA	6	2% CHG each working day with emollient	Life-threatening	Infections and infestations / Sepsis neonatal	0	0	0
ZA	3	2% CHG each working day without emollient	Life-threatening	Gastrointestinal disorders / Necrotising colitis	0	0	0
ZA	3	1% CHG each working day with emollient	Life-threatening	Gastrointestinal disorders / Necrotising colitis	0	0	0

Site	Age At Enrol (days)	Trial Arm	SAE type	SOC_PT	Skin Score Day0	Skin Score Day3	Skin Score Day8
ZA	1	Control	Hospitalisation/ prolonged hosp	Respiratory, thoracic and mediastinal disorders / Neonatal respiratory distress syndrome	1	1	0
ZA	3	2% CHG alternate working days without emollient	Hospitalisation/ prolonged hosp	Vascular disorders / Thrombophlebitis	0	0	0
ZA	6	2% CHG each working day with emollient	Other	Pregnancy, puerperium and perinatal conditions / Anaemia neonatal	0	0	0

Note: ZA=South Africa, BD=Bangladesh.

20.ACCEPT analyses of percentage of children with any SAEs

Acceptability threshold (percentage points)	1% CHG	2% CHG	Working days	Emollient	Control
-20	100%	100%	99%	98%	93%
-10	99%	99%	90%	84%	72%
-5	96%	96%	70%	58%	53%
0	89%	90%	29%	18%	34%
5	74%	75%	7%	2%	21%
10	55%	55%	2%	0%	12%
20	20%	19%	0%	0%	3%

Probability SAE rate compared to the 0.5% CHG/alternate working days/no emollient reference group is more than the acceptability threshold under non-informative priors (default priors from Stan). E.g Probability 1% CHG causes a higher rate of SAEs than 0.5% CHG (i.e. acceptability threshold of 0) is 89%. The probability of the rate of SAEs in 1% CHG being at least 5 percentage points higher than 0.5% CHG is 74%. Default priors were used as elicitation of expert opinion of priors was not deemed feasible as all members of the trial team had viewed the results.

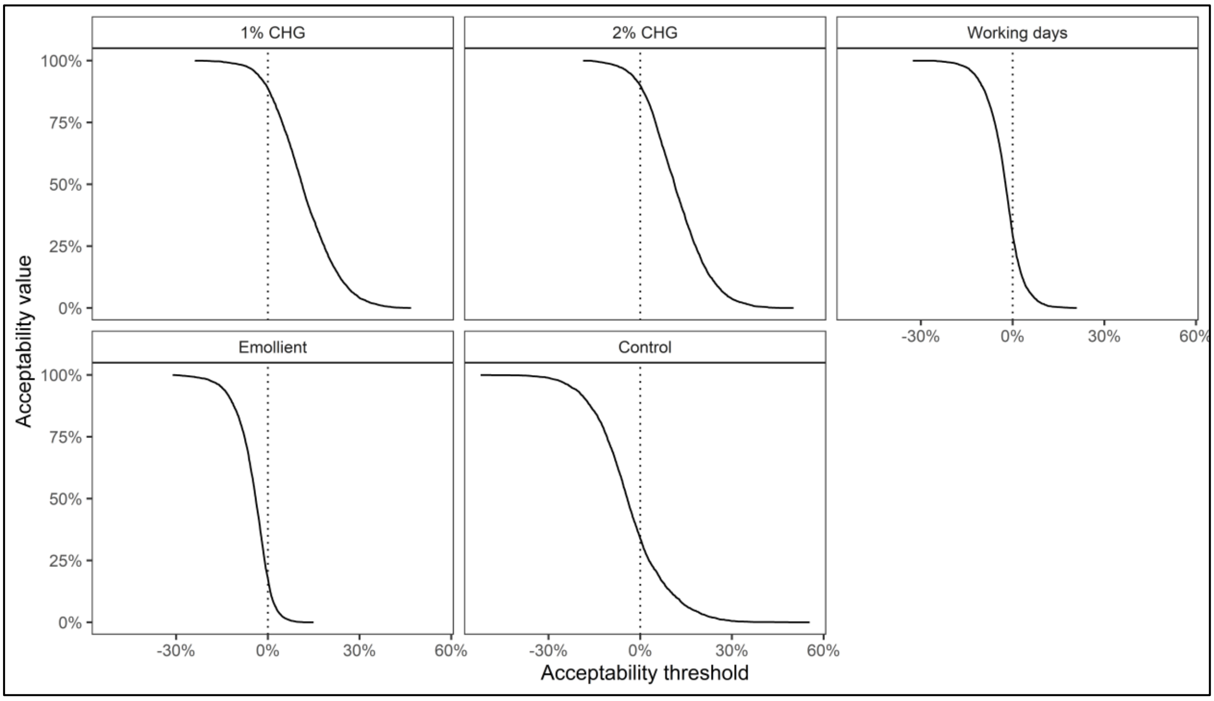

1. Dramowski A, Pillay S, Bekker A, et al. Impact of 1% chlorhexidine gluconate bathing and emollient application on bacterial pathogen colonization dynamics in hospitalized preterm neonates–A pilot clinical trial. *EClinicalMedicine* 2021; **37**: 100946.

2. Johnson J, Suwantarat N, Colantuoni E, et al. The impact of chlorhexidine gluconate bathing on skin bacterial burden of neonates admitted to the Neonatal Intensive Care Unit. *Journal of Perinatology* 2019; **39**(1): 63-71.
3. Sankar M, Paul V, Kapil A, et al. Does skin cleansing with chlorhexidine affect skin condition, temperature and colonization in hospitalized preterm low birth weight infants?: a randomized clinical trial. *Journal of Perinatology* 2009; **29**(12): 795-801.

NeoCHG

Efficacy and safety of whole-body chlorhexidine gluconate (CHG) cleansing in reducing bacterial skin colonisation of hospitalised neonates: A pilot trial

Version: 1.0
Date: 06-Mar-2020

MRC CTU at UCL ID:
ISRCTN #: ISRCTN69836999

Authorised by:
Name: Professor Mike Sharland
Role: Chief Investigator
Signature:
Date: 06-Mar-2020

Name: Michelle Clements
Role: Trial Statistician
Signature:

Date: 06-Mar-2020

This protocol has been produced using MRC CTU at UCL Protocol Template version 7.0. The template, but not any study-specific content, is licensed under a Creative Commons Attribution 4.0 International License (https://creativecommons.org/licenses/by/4.0/). Use of the template in production of other protocols is allowed**,** but MRC CTU at UCL must be credited.

CONTENTS

CONTENTS **3**

GENERAL INFORMATION **6**

LAY SUMMARY **10**

TRIAL SCHEMA **11**

SUMMARY OF TRIAL **13**

TRIAL ASSESSMENT SCHEDULE **15**

ABBREVIATIONS **16**

1 BACKGROUND **19**
1.1 THE BURDEN OF HEALTHCARE ASSOCIATED INFECTION AND ANTIMICROBIAL RESISTANCE IN NEONATES **19**
1.2 EXISTING EVIDENCE FOR CHLORHEXIDINE IN SEPSIS PREVENTION **19**
1.2.1 Cord application 19
1.2.2 Skin application 19
1.3 EXISTING EVIDENCE FOR EMOLLIENT APPLICATION IN SEPSIS PREVENTION **20**
1.4 CLINICAL EXPERIENCE OF CHLORHEXIDINE IN LOW BIRTH WEIGHT NEONATES **20**
1.4.1 Skin 20
1.4.2 Eyes 20
1.4.3 Body temperature 21
1.4.4 Cerebrospinal fluid administration 21
1.4.5 Regulatory agency recommendations 21
1.4.6 Rationale for the pilot trial 21
1.4.7 Rationale for design of NeoCHG 22

2 SELECTION OF SITES AND CLINICIANS **23**
2.1 SITE AND INVESTIGATOR INCLUSION CRITERIA **23**
2.1.1 PI's Qualifications & Agreements 23
2.1.2 Adequate Resources 24
2.2 SITE ASSESSMENT, APPROVAL AND ACTIVATION **24**

3 SELECTION OF PATIENTS **26**
3.1 PATIENT INCLUSION CRITERIA **26**
3.2 PATIENT EXCLUSION CRITERIA **26**
3.3 NUMBER OF PATIENTS **26**
3.4 CO-ENROLMENT GUIDELINES **26**
3.5 SCREENING PROCEDURES & PRE-RANDOMISATION INVESTIGATIONS **26**

4 RANDOMISATION **28**
4.1 RANDOMISATION PRACTICALITIES **28**
4.2 RANDOMISATION CODES & UNBLINDING **28**
4.3 CO-ENROLMENT GUIDELINES AND REPORTING **28**

5 TREATMENT OF PATIENTS 29
5.1 INTRODUCTION 29
5.2 TRIAL TREATMENTS 29
5.2.1 Chlorhexidine 29
5.2.2 emollient 31
5.3 CONTROL GROUP 32
5.3.1 Products 32
5.3.2 Compliance & Adherence 32
5.4 UNBLINDING / UNMASKING 32
5.5 PROTOCOL TREATMENT DISCONTINUATION 32
5.6 COMPLIANCE & ADHERENCE 33
5.7 DATA COLLECTION 33
5.8 NON-TRIAL TREATMENT 33
5.8.1 Medications Permitted 33
5.8.2 Medications Not Permitted 33
5.8.3 Medications to be Used With Caution 33
5.8.4 Treatment After Trial Event 33
5.9 CO-ENROLMENT GUIDELINES 33

6 ASSESSMENTS & FOLLOW-UP 34
6.1 TRIAL ASSESSMENT SCHEDULE 34
6.2 PROCEDURES FOR ASSESSING EFFICACY 34
6.3 PROCEDURES FOR ASSESSING SAFETY 34
6.4 OTHER ASSESSMENTS 35
6.5 EARLY STOPPING OF FOLLOW-UP 35
6.6 PATIENT TRANSFERS 35
6.7 FOLLOW-UP 35
6.8 COMPLETION OF PROTOCOL FOLLOW UP 35

7 SAFETY REPORTING 36
7.1 DEFINITIONS 36
7.1.1 Product 36
7.1.2 Adverse Events 37
7.1.3 Safety management 37
7.1.4 Exempted Adverse Events 37
7.2 INVESTIGATOR RESPONSIBILITIES 37
7.3 INVESTIGATOR ASSESSMENT 38
7.3.1 Seriousness 38
7.3.2 Severity or Grading of Adverse Events 38
7.3.3 Causality 38
7.3.4 Notification 39
7.4 MRC CTU RESPONSIBILITIES 39

8 QUALITY ASSURANCE & CONTROL 40
8.1 RISK ASSESSMENT 40
8.2 CENTRAL MONITORING AT THE MRC CTU 40
8.3 ON-SITE MONITORING 40
8.3.1 Direct Access to Patient Records 40
8.3.2 Confidentiality 41

9 STATISTICAL CONSIDERATIONS 42
9.1 METHOD OF RANDOMISATION 42

9.2 OUTCOME MEASURES **42**
9.2.1 Co-Primary Outcome Measures 42
9.2.2 Secondary Outcome Measures 42
9.3 SAMPLE SIZE **42**
9.4 INTERIM MONITORING & ANALYSES **42**
9.5 ANALYSIS PLAN (BRIEF) **43**

10 REGULATORY & ETHICAL ISSUES **45**
10.1 COMPLIANCE **45**
10.1.1 Regulatory Compliance 45
10.1.2 Site Compliance 45
10.1.3 Data Collection & Retention 45
10.2 ETHICAL CONDUCT **45**
10.2.1 Ethical Considerations 45
10.2.2 Ethical approvals 46
10.3 TRIAL CLOSURE **46**

11 INDEMNITY **47**

12 FINANCE **48**

13 OVERSIGHT & TRIAL COMMITTEES **49**
13.1 TRIAL MANAGEMENT GROUP (TMG) **49**
13.2 INDEPENDENT DATA MONITORING COMMITTEE (DMC) **49**
13.3 ROLE OF STUDY SPONSOR **49**

14 PATIENT AND PUBLIC INVOLVEMENT **50**

15 PUBLICATION AND DISSEMINATION OF RESULTS **51**

16 DATA AND/OR SAMPLE SHARING **53**

17 PROTOCOL AMENDMENTS **54**

APPENDIX I SKIN SCORE ASSESSMENT **55**

APPENDIX II ADVERSE EVENT GRADING **57**

REFERENCES **59**

GENERAL INFORMATION

This document was constructed using the Medical Research Council (MRC) Clinical Trials Unit (CTU) at University College London (UCL) (MRC CTU at UCL, henceforth the 'MRC CTU') Protocol Template Version 7.0. The MRC CTU endorses the Standard Protocol Items: Recommendations For Interventional Trials (SPIRIT) initiative. It describes the NeoCHG trial, coordinated by the MRC CTU, and provides information about procedures for entering participants into it. The protocol should not be used as an aide-memoire or guide for the treatment of other patients. Every care has been taken in drafting this protocol, but corrections or amendments may be necessary. These will be circulated to the registered investigators in the trial, but sites entering patients for the first time are advised to contact the trial team to confirm they have the most up-to-date version.

Chlorhexidine gluconate will be denoted with the abbreviation 'CHG' or as 'chlorhexidine' throughout the protocol.

COMPLIANCE

The trial will be conducted in compliance with the approved protocol, the Declaration of Helsinki 1996 (seventh revision), the principles of Good Clinical Practice (GCP) as laid down by the ICH topic E6, and applicable national regulations. For the purposes of this trial, CHG is regarded as a Clinical Trial of an Investigational Medicinal Product (CTIMP) in South Africa but not in Bangladesh.

SPONSOR

St George's, University of London is the trial Sponsor and has delegated responsibility for the overall management of the NeoCHG trial to the MRC CTU. Queries relating to sponsorship of this trial should be addressed St George's, University of London. All questions about any aspects of trial management should be directed to the trial management team at the MRC CTU.

FUNDING

MRC/NIHR/DfID/Wellcome Joint Global Health Trials Call 9 – Trial Development Grant.
Medical Research Council for core support to the MRC CTU.

AUTHORISATIONS AND APPROVALS

This trial will be submitted for approval by Research Ethics Committees (REC)/Institutional Review Boards (IRB) in each of the participating countries. For oversight, the REC associated with the trial's Sponsor will review the trial protocol and relevant trial documentation.

TRIAL REGISTRATION

This trial will be registered via the ISRCTN Clinical Trial Register, where it is identified as <<TO BE ADDED FOLLOWING COMPLETION OF REGISTRATION (in progress)>>

SAE REPORTING
Within 24 hours of becoming aware of an SAE, please scan and send via encrypted email a completed SAE form to the MRC CTU on **mrcctu.neochg@ucl.ac.uk**

TRIAL SPONSOR

SPONSOR

St George's, University of London (SGUL)
Paediatric Infectious Diseases Research Group
Institute for Infection and Immunity
2nd Floor, Jenner Wing,
Cramner Terrace
London
United Kingdom
SW17 0RE

Switchboard: +44 (0)208 725 0666/5382
Fax: +44 (0)208 8725 0716

ST GEORGE'S, UNIVERSITY OF LONDON STAFF

Chief Investigator: Prof Mike Sharland
MBBS BSc MRCP MD DTM&H FRCPCH
Tel: +44 (0)208 725 5382
Email: msharland@sgul.ac.uk

Co-Investigator: Dr Neal Russell
MBBS BSc MRCPCH DTM&H PGCert
Tel: +44 (0)208 725 5382
Email: nrussell@sgul.ac.uk

Co-investigator: Dr Theoklis Zaoutis
MSCE MD PhD
Tel: : +44 (0)208 725 5382
Email: tzaoutis@sgul.ac.uk

Operations Manager: Dr Tatiana Munera Huertas
PhD
Tel: +44 (0)208 725 5382
Email: tmunerah@sgul.ac.uk

Project Manager: Yasmine Yau
Tel: +44 (0)208 725 5382
Email: yyau@sgul.ac.uk

TRIAL ADMINISTRATION

Please direct all queries to the Trial Management Team at the MRC CTU in the first instance; clinical queries will be passed to the Chief Investigator at SGUL via the team as appropriate.

COORDINATING CLINICAL TRIALS UNIT

MRC Clinical Trials Unit at UCL
90 High Holborn
London
United Kingdom
WC1V 6LJ

Switchboard: +44 (0)20 7670 4700
Fax: +44 (0)20 7670 4814
Email: mrcctu.neochg@ucl.ac.uk

MRC CTU STAFF

Trial Statistician & Co-Investigator: Dr Michelle Clements PhD MSCi
Tel: +44 (0)207 670 4912
Email: michelle.clements@ucl.ac.uk

Project Leader & Co-Investigator: Prof Sarah Walker FMedSci PhD MSC
Tel: +44 (0)207 670 4726
Email: rmjlasw@ucl.ac.uk

Clinical Project Manager: Dr Francesca Schiavone PhD MSc
Tel: +44 (02)07 670 4900
Email: f.schiavone@ucl.ac.uk

Trial Manager: TBA
Tel:
Email:

Data Manager: Peter Skoutari MSc
Tel: +44 (0)207 670 4773
Email: p.skoutari@ucl.ac.uk

RECRUITING SITE STAFF

Bangladesh
Lead Site
Child Health Research Foundation (CHRF)
Department of Microbiology
Dhaka Shishu Hospital
Sher-E-Banglanagar
Dhaka 1207
Bangladesh

Principal Investigator: Prof Samir Saha BSc MSc PhD
Tel: +880 29138594
Email: samirk.sks@gmail.com

Co-investigator: Mr Mohammad Shahidul Islam BSc Msc MSPH
Tel: +8801787698256
Email: shahidul@chrfbd.org

South Africa
Lead site
Stellenbosch University
Private Bag X1, Matieland
7602, Stellenbosch
South Africa

Co- principal Investigator: Prof Adrie Bekker MBChB DCH FCPaed MMed PhD
Tel: +27 21 938 9506
Email: adrie@sun.ac.za

Co- principal Investigator: Prof Andrew Whitelaw MBBCh MSc FCPath
Tel: 27 21 938 4032 / 938 4066
Email: awhitelaw@sun.ac.za

Co- principal Investigator: Prof Angela Dramowski MBChB DCH FCPaed MMed PhD
Tel: +27 21 938 9506
Email: dramowski@sun.ac.za

Other Responsible Individuals

The Global Antibiotic Research and Development Partnership (GARDP)
15 Chemin Louis-Dunant
1202 Geneva
Switzerland

Project Leader: Sally Ellis
Email: sellis@gardp.org

LAY SUMMARY

This is a pilot study to assess the best way of applying antiseptic to low birth weight newborn babies who are in hospital. The antiseptic aims to reduce the amount of potentially harmful bacteria on the skin of newborn babies. This might be useful for preventing infections which are picked up in hospital.

This study aims to find out the best concentration of antiseptic to use, as well as how often it should be put on the babies, and whether or not it should be combined with a skin softener. In this pilot study, we will see the amount of bacteria babies have on their skin, and look carefully at whether, and how often, skin reactions occur.

The antiseptic we will test in this study is called chlorhexidine. Chlorhexidine has been widely used across the world for many decades to reduce the risk of babies dying from infection. For example, it is put on the umbilical cord of newborn babies at home in areas with high rates of deaths from infections. However, we don't know whether applying chlorhexidine to the body could reduce the risk of infection and death in newborn babies who are in hospital and have a high risk of infection because of other problems, like being premature or low birth weight.

In addition, skin softeners (also called emollients), which are often in the form of oils such as sunflower oil, have been shown in some, but not all, studies to reduce infections. We do not know whether combining emollients and an antiseptic may work even better, so we will also look at this in this pilot study.

The study will be conducted in two hospitals, one in Bangladesh, and the other in South Africa. Chlorhexidine will be put on the skin of the body of some of the babies, starting at 1-6 days of age and either on working days or on alternate working days of the week.

Different babies will get different concentrations of chlorhexidine. Some babies will also get skin softeners applied. And some babies will just be followed as normal. A computer will choose which babies get which treatment. Skin swabs will be taken from various areas of the skin of the babies, and the amount and type of bacteria on these swabs will be counted.

In addition, the skin of the babies will be closely monitored for any signs of side effects to chlorhexidine. Applying an antiseptic can make babies colder and can sometimes cause skin reactions which may make it easier for bacteria to get into the babies instead of protecting them. But we don't know how these different advantages and disadvantages balance out. This study will look at the balance between reducing numbers of bacteria and safety. In the future, we plan to test the best method to reduce the number of bacteria on newborn babies' skin we find in this study in a much larger study, to see whether it could reduce serious infections or not.

TRIAL SCHEMA

Figure 1: Trial Schema

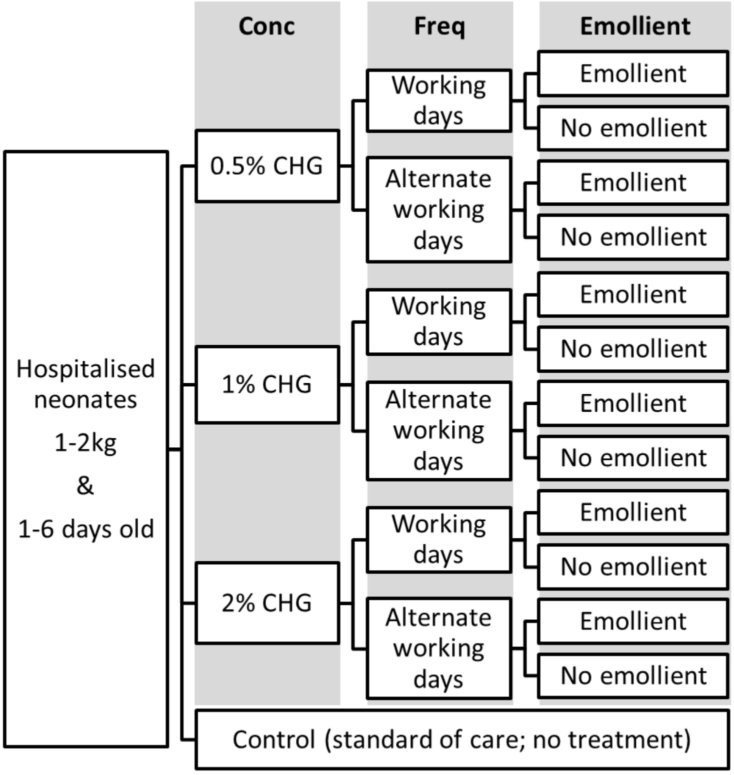

Follow up to 28 days.
Last application of CHG at day 14 after enrolment or discharge (whichever earlier)

Co-primary outcome(s)

- Change in bacterial load from baseline to final swab (efficacy)
- Adapted Neonatal Skin Condition Score (safety) (absolute score and grade)

Secondary outcomes

- Temperature (change in absolute temperature and grade (hypothermia))
- Acquisition & loss of specific bacterial species
  - Enterobacteriaceae
  - Acinetobacter
  - *Staphylococcus aureus*
  - *Beta haemolytic streptococci (group A and B)*
  - *Enterococcus*
  - *Candida*
- Serious adverse events

Note: CHG=chlorhexidine gluconate. Alternate working days are Monday, Wednesday, Friday; or Tuesday, Thursday, Saturday, depending on the standard working pattern in each country.

Figure 2: Trial Entry, Randomisation and Treatment

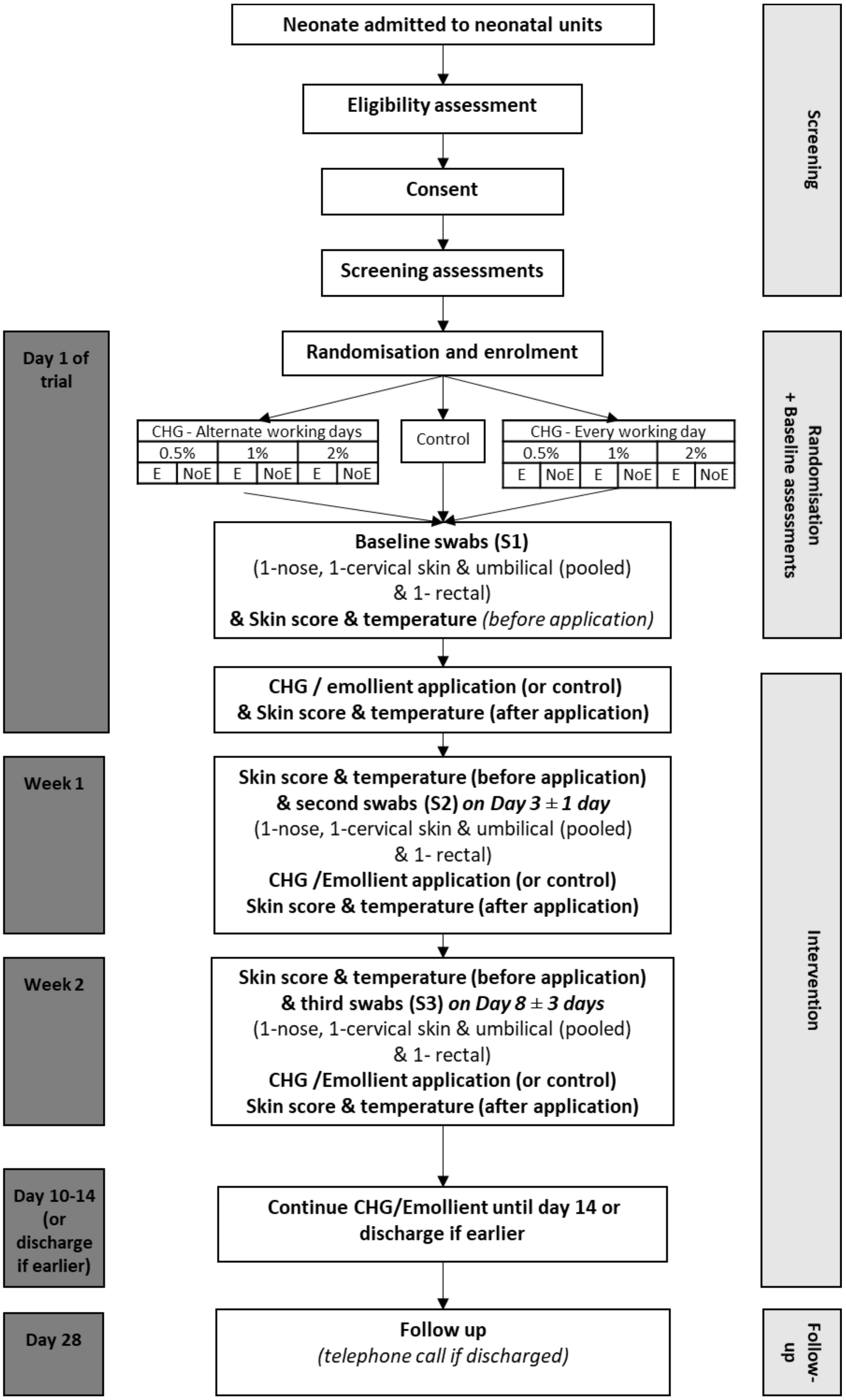

*E= Emollient (same timing as CHG) *NoE = No Emollient.

Note: CHG=chlorhexidine gluconate. Alternate working days are Monday, Wednesday, Friday; or Tuesday, Thursday, Saturday, depending on the standard working pattern in each country.

SUMMARY OF TRIAL

SUMMARY INFORMATION TYPE	SUMMARY DETAILS
**Short title**	NeoCHG
**Long title**	Efficacy and safety of whole-body chlorhexidine (CHG) cleansing in reducing bacterial skin colonisation of hospitalised low birth weight neonates: A pilot trial
**Version**	1.0
**Date**	06-Mar-2020
**ISRCTN #**	ISRCTN69836999
**Study Design**	Factorial randomised controlled pilot trial
**Setting**	Hospital neonatal units in South Africa and Bangladesh
**Type of Participants to be Studied**	Hospitalised low birth weight (1-2kg) babies between the ages of 1-6 days at enrolment
**Ancillary Studies/Sub-studies**	None
**Sponsor**	St George's, University of London
**Interventions to be Compared**	Chlorhexidine gluconate (CHG) whole body skin application in varying concentrations (2% vs 1% vs 0.5%) and varying frequency, each with or without emollient application, compared to a control group with no CHG or emollient.
**Study Hypothesis**	Increasing concentration and frequency of CHG skin application leads to greater reductions in bacterial colonisation.<br>The addition of emollient improves the skin condition and safety of CHG application, permitting the more effective concentration to be used.
**Primary Outcome Measure(s)**	Co-primary outcomes are:<br>• Change in bacterial load from baseline to final swab (efficacy)<br>• Adapted neonatal skin condition score (safety) (absolute score and grade)
**Secondary Outcome Measure(s)**	• Temperature (change in absolute temperature and grade (hypothermia))<br>• Acquisition & loss of specific bacterial species:<br>◦ Enterobacteriaceae<br>◦ Acinetobacter<br>◦ Staphylococcus aureus<br>◦ Beta haemolytic streptococci (group A and B)<br>◦ Enterococcus<br>◦ Candida<br>• SAEs
**Randomisation**	Factorial randomised controlled trial,<br>Allocated 1:1:1:1:1:1:1:1:1:1:1:1:1 into 12 intervention arms and 1 control arm<br>0.5% CHG each working day with emollient<br>0.5% CHG each working day without emollient

SUMMARY INFORMATION TYPE	SUMMARY DETAILS
	0.5% CHG alternate working days with emollient<br>0.5% CHG alternate working days without emollient<br><br>1% CHG each working day with emollient<br>1% CHG each working day without emollient<br>1% CHG alternate working days with emollient<br>1% CHG alternate working days without emollient<br><br>2% CHG each working day with emollient<br>2% CHG each working day without emollient<br>2% CHG alternate working days with emollient<br>2% CHG alternate working days without emollient<br><br>Control Group without emollient and without CHG<br>(Alternate working days are Monday, Wednesday, Friday; or Tuesday, Thursday, Saturday, depending on the standard working pattern in each country.)
**Number of Participants to be Studied**	182 (91 in each site) (babies without baseline and two post-baseline swabs on which to assess the primary efficacy endpoint will be replaced)
**Duration**	12 months to complete recruitment and follow-up. Individual follow up during hospital admission up to day 14 after enrolment or discharge if earlier, and final follow up 28 days after enrolment (by phone If already discharged)
**Funder**	MRC/NIHR/DfID/Wellcome Joint Global Health Trials Call 9 – Trial Development Grant & MRC Core Funding (MRC CTU at UCL)
**Chief Investigator**	Professor Mike Sharland

TRIAL ASSESSMENT SCHEDULE

TABLE 1: Trial Assessment Schedule

ASSESSMENTS		DAYS IN TRIAL								
		Week 1					Week 2			Final follow up
**Face to face visit** ☐<br>**Face to face or telephone** ☐	Screening D-1 to D1	Random-isation D1	Day 1	Day 2	Day 3	Day 4	Day 5	Day 8	Day 9	Day 10-14[i]	Day 28
**Trial participation**											
**Eligibility**	X										
**Parent/carer information sheet**	X										
**Informed consent**	X										
**Enrolment form and randomisation**[a]		X									
**Clinical assessments**											
**Baseline data collection** [b]	X										
**Weight**	X									X	
**Temperature** [c]	X		X	X	X	X	X	X	X	X[i]	
**Skin Score**[d]	X		X	X	X	X	X	X	X	X[i]	
**Adverse Events**	X		X	X	X	X	X	X	X	X[i]	
**Microbiology**											
**Nose swab** [e]			X		X[e]			X[e]			
**Cervical skin fold & umbilical swabs (pooled)**[f]			X		X[f]			X[f]			
**Rectal swab** [g]			X		X[g]			X[g]			
**CHG ± emollient application** [h]			X	(X)	X	(X)	X	(X)	X	X[i]	
**Final follow up**											X[j]

Additional explanatory notes for investigations

Intervention & assessment days are on working days – if a day falls on a non-working day then shift next activity to the closest working day, whatever the working pattern. Alternate working days are Monday, Wednesday, Friday; or Tuesday, Thursday, Saturday, depending on the standard working pattern in each country.

[a] Enrolment form completed and data entered into the database. Randomisation completed electronically.

[b] Includes collecting medical history, mode of delivery, birth weight, gestational age, co-morbidities and intrapartum and antibiotic history.

[c] Temperature to be collected once at screening. Temperature to be collected BEFORE every CHG ± emollient application and 15 minutes POST application. Skin score, temperature and AEs will be monitored daily in all groups.

[d] Skin to be assessed according to the skin score in Appendix I, once at screening. Skin score to be assessed BEFORE every CHG ± emollient application and 30 minutes POST application.

[e] Nose swab to be taken BEFORE any CHG ± emollient application on day 1, day 3± 1 day and day 8 ± 3 days (closest working day) and at any time on this day in the control group. See Manual of Operations (MOP) for further details on how to take swabs.

[f] A pooled swab of the cervical skin and umbilicus to be taken BEFORE any CHG ± emollient application on day 1, day 3± 1 day and day 8 ± 3 days (closest working day) and at any time on this day in the control group. See MOP for further details on how to take swabs.

[g] Peri-rectal swab to be taken BEFORE CHG ± emollient application on day 1, day 3± 1 day and day 8 ± 3 days (closest working day) and at any time on this day in the control group. See MOP for further details on how to take swabs.

[h] CHG ± emollient to be applied either every working day or alternative working days according to randomised allocation.

[i] CHG± emollient application to be continued on every working day or alternative working days (according to randomisation) until day 14 or discharge, whichever is sooner.

[j] phone call if discharged. Vital status will be ascertained, together with occurrence of serious adverse events (including clinically defined sepsis, necrotising enterocolitis (NEC)) and any unscheduled visits to healthcare providers

ABBREVIATIONS

Abbreviation	Expansion
AE	Adverse event
AMR	Antimicrobial resistance
AR	Adverse reaction
CF	Consent Form
CFU	Colony forming units
CI	Chief Investigator
CI	Confidence interval
CHG	Chlorhexidine gluconate
COM	Clinical Operations Manager
CPM	Clinical Project Manager
CRF	Case Report Form
CTU	*See MRC CTU at UCL*
D	Day
DCF	Data Clarification Form
DM	Data Manager
DMC	Independent Data Monitoring Committee
DMP	Data Management Plan
DNDi	Drugs for Neglected Diseases Initiative
EU	European Union
GARDP	Global antibiotic research and development partnership
GCP	Good Clinical Practice
HAI	Healthcare Associated Infection
IMPAACT	International Maternal Pediatric Adolescent AIDS Clinical Trials Network
IMP	Investigational medicinal product
IRB	Institutional Review Board
ISRCTN	International Standard Randomised Controlled Trial Number

Abbreviation	Expansion
ITT	Intention-to-treat
LMIC	Low or middle income country
MedDRA	Medical Dictionary for Regulatory Activities
MHRA	Medicines and Healthcare products Regulatory Agency
MOP	Manual of Operations
MRC	Medical Research Council
MRC CTU at UCL	Medical Research Council Clinical Trials Unit at University College London (also generally abbreviated to "CTU")
NEC	Necrotising enterocolitis
NeoAMR	Neonatal antimicrobial resistance (programme)
NIMP	Non-investigational-medicinal product
PENTA	Paediatric European Network for Treatment of AIDS
PI	Principal Investigator
PIS	Patient Information Sheet
QMAG	Quality Management Advisory Group
RCT	Randomised controlled trial
REC	Research Ethics Committee
RGC	Research Governance Committee
SAE	Serious adverse event
SAP	Statistical Analysis Plan
SAR	Serious adverse reaction
SD	Standard deviation
SGUL	St George's University of London
SOP	Standard operating procedure
SmPC	Summary of Product Characteristics
SSA	Site-specific approval
SUSAR	Suspected unexpected serious adverse reaction
TMF	Trial Master File

ABBREVIATION	EXPANSION
TMG	Trial Management Group
TSC	Trial Steering Committee
UAR	Unexpected adverse reaction
WHO	World Health Organization

1 BACKGROUND

1.1 THE BURDEN OF HEALTHCARE ASSOCIATED INFECTION AND ANTIMICROBIAL RESISTANCE IN NEONATES

Neonatal sepsis is a substantial cause of neonatal mortality,[1,2] with an estimated 5.1 million cases and over 800,000 related deaths each year.[3] The incidence of neonatal sepsis and burden of related deaths is highest in low and middle income settings (LMICs). Healthcare associated infections (HAI) due to horizontal transfer of pathogenic bacteria in hospital environments accounts for a substantial proportion of neonatal sepsis,[4] and are likely to increase as facility delivery rates increase globally.[5] HAI is also an important driver of antimicrobial resistance (AMR), which is increasing in many settings.[6,7] An estimated 214,000 neonatal deaths per year are attributable to AMR,[8] and although estimates are based on limited data, largely from high income countries, it is clear that the neonatal period is particularly important in terms of attributable deaths and disability adjusted life years lost due to AMR,[9–11] with multidrug resistant Gram-negative bacilli being of particular concern. Given increasing HAI and decreasing treatment options, new prevention strategies are urgently needed to achieve global reductions in neonatal mortality, as aspired to in the Sustainable Development Goals.[12] Indeed without new strategies the problem of HAI and associated AMR is likely to continue to increase, potentially leading in many settings to infections which are untreatable with available antibiotics. This potential lack of available treatment makes investigating better prevention methods all the more urgent.

1.2 EXISTING EVIDENCE FOR CHLORHEXIDINE IN SEPSIS PREVENTION

Colonisation with pathogenic bacteria precedes invasive infections, and preterm low birthweight neonates with immature skin are particularly vulnerable to trans-epithelial bacterial invasion. Antiseptics such as chlorhexidine (CHG), which reduce bacterial load on the skin,[11] may reduce the risk of neonatal sepsis, thereby reducing neonatal mortality. Chlorhexidine is active against Gram positive and Gram negative organisms and is commonly used for skin disinfection in neonates for invasive procedures.[13] However, wider skin application could potentially prevent colonisation with multi-resistant hospital acquired organisms, and hence avoid their subsequent invasion and the development of neonatal sepsis.

1.2.1 CORD APPLICATION

Randomised trials, and a systematic review, have shown that 4% chlorhexidine cord application reduces mortality in term neonates in community settings with high background neonatal mortality rates.[14,15] Mortality was not reduced for cord application only in settings with lower baseline mortality risk[16] or in hospital settings, although infection of the umbilical stump (omphalitis) was reduced.[14] WHO now recommends chlorhexidine application to the cord daily in the first week of life for all babies born at home in community settings with baseline neonatal mortality rates of above 30/1000 live births, and it is now on the WHO Essential Medicines list.

1.2.2 SKIN APPLICATION

Chlorhexidine skin application reduces bacterial colonisation similarly to 4% chlorhexidine to the umbilical cord,[17,18] raising the question of whether skin application could be effective in high risk, low birthweight hospitalised neonates. However, recent systematic reviews of chlorhexidine skin wipes for reducing neonatal sepsis and mortality have been inconclusive,[14] and recommended further

research. Importantly, trials have so far predominantly included neonates of normal birth weight (>2500g), who are at low overall risk of infection.[19,20] In a subgroup analysis of a community based trial of chlorhexidine skin wipes, mortality was significantly reduced in low birth weight neonates,[21] suggesting that such high risk subgroups require further study.

The effect on bacterial load of chlorhexidine is transient and unlikely to last longer than 48-72hrs.[17,18] Most studies of chlorhexidine in neonates assessed a single application soon after birth,[14,22–24] which is potentially insufficient for targeting HAI in neonatal units where the median onset of neonatal sepsis is nine days after birth (IQR 6-13; unpublished data from trial sites). It is also unknown whether repeated application of chlorhexidine could prevent HAI in these high-risk neonates.

1.3 EXISTING EVIDENCE FOR EMOLLIENT APPLICATION IN SEPSIS PREVENTION

Emollients have not consistently reduced bacterial colonisation,[25] but it is thought that they may act as a barrier to bacterial invasion, potentially providing lipids which integrate into the dermis. Emollient application has been shown to reduce episodes of neonatal sepsis in some studies in preterm neonates,[26] particularly in LMIC settings; however, a recent Cochrane review was inconclusive.[25] The potential benefit (and safety) of combining chlorhexidine with an emollient, potentially reducing both bacterial colonisation and invasion, has not been explored.

1.4 CLINICAL EXPERIENCE OF CHLORHEXIDINE IN LOW BIRTH WEIGHT NEONATES

1.4.1 SKIN

Chlorhexidine has been used for decades as a key part of routine care for newborn babies, including premature and low birth weight babies, and for invasive procedures.[13] Indeed, as above, chlorhexidine is also on the WHO Essential Medicines list for cord care for prevention of neonatal mortality. The main potential safety issues related to chlorhexidine have been raised predominantly in extremely premature and low birth weight newborn babies (especially <1kg and <28 weeks)[27] where there have been some skin reactions, particularly when chlorhexidine is combined with alcohol and more rarely with water based chlorhexidine. The risk of skin damage is very low and has been associated with using larger quantities of chlorhexidine, particularly if it is allowed to pool in contact with the skin.[27–30]

In this trial the risks will be minimised by the following aspects of the design:

- Excluding the babies at the highest risk (<1kg and <28 weeks)
- Using chlorhexidine without alcohol, delaying the start until after 24 hrs of life
- Strict operating procedures to prevent the risk of skin damage from pooling of liquid
- Close observation using a skin score assessment based on previous studies[26,31] but including assessment of the full range of conditions associated with chlorhexidine and emollient
- Early discontinuation of chlorhexidine with signs of skin damage.

When chlorhexidine has been used in numerous previous studies including similar populations of babies, skin damage has been very rare.[14,19,21,32,33]

1.4.2 EYES

Chlorhexidine is not intended for use in the eyes, and will be completely avoided on the face in this trial, therefore there should be no risk of eye damage. Eye damage has only occurred when higher concentrations (4% chlorhexidine) have been used directly on the eye by mistake.[34] In this trial there

will be clear and strict guidance not to use chlorhexidine anywhere near the face, the ears or eyes, and there will indeed be no reason to mistake the product for an eye product, as the packaging will be entirely different.

1.4.3 Body temperature

A drop in body temperature has occasionally been documented after chlorhexidine skin application with soaked cotton wool buds applied immediately after birth.35 Application will be performed in a warm environment and temperature will be monitored both before and 15 minutes after application.

1.4.4 Cerebrospinal fluid administration

Although chlorhexidine has been detected in blood, no neurotoxicity has been reported with skin application despite large studies and widespread routine use for invasive procedures. Indeed neurotoxicity has only been documented when chlorhexidine is inadvertently introduced into the cerebrospinal fluid during intra-thecal procedures, which are not part of this trial.[36] In addition, cord application, which is recommended by WHO at concentrations of 4%, has been associated with higher blood levels than skin application, with no adverse effects reported despite repeated applications.[15,32]

1.4.5 Regulatory agency recommendations

The UK's Medicines and Healthcare Products Research Agency (MHRA) and European Medicines Agency in 2014 issued an alert about potential skin damage with chlorhexidine, focussing on preterm neonates[37] although chlorhexidine continues to be widely used in this group for invasive procedures.[13] In this trial the recommendations provided by these regulatory agencies (including use with care and monitoring of skin condition, and avoidance of pooling of liquid) will be closely followed. WHO has warned of the risk of inadvertent administration in the eyes of 4% chlorhexidine intended for use on the cord[34] and the face will be avoided accordingly.

1.4.6 Rationale for the pilot trial

As discussed above, the very high rates of nosocomial infections now reported from neonatal units globally are of very high public health concern. There are currently no proven interventions to reduce acquisition of multidrug resistant pathogens for babies admitted to neonatal units. There are also very few trials underway attempting to answer this important question. Any potential intervention would need to be widely available and low cost. A novel chlorhexidine based intervention would have significant advantages for future implementation. However, questions remain about what concentration of chlorhexidine, used at what frequency, with or without emollient would best balance risks and benefits such that it could be tested in a large randomised trial in a hospital setting with neonatal sepsis as the primary endpoint. In particular, while evidence has shown benefits from cord application of chlorhexidine in community settings, there is a need for further research to determine whether chlorhexidine skin application could be used in hospital settings to reduce HAI.

The specific aim addressed by NeoCHG is to determine which chlorhexidine body cleansing regime in low birthweight hospitalized neonates should be taken forward to a larger controlled trial.

Results from the pilot will determine the specific chlorhexidine cleansing regime to be tested in the future trial aiming to reduce deaths from neonatal sepsis due to HAIs in LMIC settings. Clorhexidine cleansing would be a key component in part of a bundle of interventions.

1.4.7 RATIONALE FOR DESIGN OF NEOCHG

Given that several questions remain about the safest regimen of chlorhexidine in low birth weight newborn babies, as well as the effect of emollient, a design which permits several questions to be answered simultaneously is preferred. The factorial design enables multiple interventions to be tested more efficiently than comparing each intervention separately. Including a control group enables the effect of chlorhexidine to be compared to the current standard of care.

2 SELECTION OF SITES AND CLINICIANS

The trial Sponsor has overall responsibility for site and investigator selection.

NeoCHG will be conducted at 2 sites:
- Tygerberg Hospital, Cape Town, South Africa
- Dhaka Shishu Hospital, Dhaka, Bangladesh

The two hospitals in this pilot trial are part of an established network of 20 sites conducting a current observational study of neonatal sepsis (NeoOBS), as part of the NeoAMR programme of the Global Antibiotic Research and Development Partnership (GARDP), which was developed between the WHO and the Drugs for Neglected Diseases Initiative (DNDi). Both sites have a significant incidence of neonatal sepsis, AMR and HAIs, based on unpublished and published work. The NeoOBS network of sites is an ideal platform for the future trial and this application is fully supported by GARDP.

Other sites may be opened depending on trial progress. The list of sites in the protocol will not necessarily be updated with alterations to the participating sites or the details of any new sites.

2.1 SITE AND INVESTIGATOR INCLUSION CRITERIA

To participate in the NeoCHG trial, investigators and clinical trial sites must fulfil a set of essential criteria that have been agreed by the NeoCHG Trial Management Group (TMG) and are defined below. Once a site has been assessed as being compliant with the inclusion criteria, the trial team will provide the site with a copy of this protocol, a trial summary together with other essential documents for the conduct of the trial, including but not limited to the Summary of Product Characteristics (SmPC), patient-facing documents and Manual of Operations (MOPs).

Those centres that meet the criteria will be issued with the NeoCHG Investigator Site File documentation for their Site-specific Approval (SSA) and accreditation documents (to be provided by the MRC CTU). Centres will be required to complete and return the NeoCHG Accreditation Form to comply with greenlight procedures.

2.1.1 PI'S QUALIFICATIONS & AGREEMENTS

1. The investigator should be qualified by education, training, and experience to assume responsibility for the proper conduct of the trial at their site and should provide evidence of such qualifications through an up-to-date curriculum vitae and/or other relevant documentation requested by the Sponsor, the REC/IRB, and/or the regulatory authority(ies).

2. The investigator should be thoroughly familiar with the appropriate use of the investigational product(s) if appropriate, as described in the protocol, in the current SmPC in the product information and in other information sources provided by the Sponsor.

3. The investigator should be aware of, and should comply with, the principles of GCP and the applicable regulatory requirements. A record of GCP training should be accessible for all investigators.

4. The investigator/site should permit monitoring and auditing by the Sponsor, and inspection by the appropriate regulatory authority(ies).

5. The investigator is responsible for supervising any individual or party to whom the investigator delegates trial-related duties and functions conducted at the trial site.

6. If the investigator/institution retains the services of any individual or party to perform trial-related duties and functions, the investigator/institution should ensure this individual or party is qualified to perform those trial-related duties and functions and should implement procedures to ensure the integrity of the trial-related duties and functions performed and any data generated.

7. The investigator should maintain a delegation log of appropriately-qualified persons to whom the investigator has delegated significant trial-related duties.

8. The investigator should sign an investigator statement, which verifies that the site is willing and able to comply with the requirements of the trial.

2.1.2 Adequate Resources

1. The investigator should be able to demonstrate a potential for recruiting the required number of suitable subjects within the agreed recruitment period (that is, the investigator regularly treats the target population).

2. The investigator should have sufficient time to properly conduct and complete the trial within the agreed trial period.

3. The investigator should have available an adequate number of qualified staff and adequate facilities for the foreseen duration of the trial to conduct the trial properly and safely.

4. The investigator should ensure that all persons assisting with the trial are adequately informed about the protocol, the investigational product(s), and their trial-related duties and functions.

5. The site should have sufficient data management resources to allow prompt data return to CTU (described in the Data Management Plan).

2.2 SITE ASSESSMENT, APPROVAL AND ACTIVATION

Each selected clinical trial site must complete the NeoCHG Accreditation Form, which includes the protocol's cover page signed by the Principal Investigator, Signature and Delegation of Responsibilities Log, and staff contact details. Signing the protocol's cover page verifies that the site is willing, and able to comply with the requirements of the trial. This will be signed by the Principal Investigator at the site. In addition, and in compliance with the principles of GCP, all site staff participating in the trial must complete the Signature and Delegation of Responsibilities Log and forward this to the MRC CTU. The MRC CTU must be notified of any changes to trial personnel and/or their responsibilities. An up-to-date copy of this log must be stored in the Trial Master File (TMF) at the site and also at the MRC CTU.

The trial will be approved by the Sponsor's Research Ethics Committee, each participating country's Institutional review Board and any other relevant local committees prior to the initiation of recruitment.

On receipt of the relevant accreditation documents at the MRC CTU, written confirmation will be sent to the PI to confirm activation. The site's pharmacist will also be informed of the site's activation.

1. The site should conduct the trial in compliance with the protocol as agreed by the Sponsor and, if required, by the regulatory authority(ies), and which was given favourable opinion by the REC and/or IRB.
2. The PI or delegate should document and explain any deviation from the approved protocol, and communicate this with the trial team at the MRC CTU.

A list of activated sites will be collated by the Trial Manager.

3 SELECTION OF PATIENTS

There will be **no exceptions** to eligibility requirements at the time of randomisation. Questions about eligibility criteria must be addressed to the trial management team prior to attempting to randomise the baby.

The eligibility criteria are the standards used to ensure that only medically appropriate babies are considered for this trial. Babies not meeting the criteria should not join the trial. For the safety of the babies, as well as to ensure that the results of this trial can be useful for making treatment decisions regarding other babies in similar situations, it is important that no exceptions be made to these criteria for admission to the trial.

Those enrolled in the trial will be neonates who have been admitted to hospital. Babies will be considered eligible for enrolment in this trial if they fulfil all the inclusion criteria and none of the exclusion criteria as defined below.

3.1 PATIENT INCLUSION CRITERIA

1. Aged 1-6 days (post-natally) at enrolment
2. Gestational age ≥28 weeks at birth
3. Birth weight ≥1000g and <2000g (or current weight if unknown)
4. Parental consent
5. Parent’s willingness to avoid routine use of emollients other than those indicated by the randomised allocation

3.2 PATIENT EXCLUSION CRITERIA

1. Poor skin condition (skin score of 2 or more in any of three domains (see **Appendix I**)) at the time of enrolment
2. Known congenital or acquired skin disorder or defect at time of enrolment
3. Anticipated length of hospital stay <7 days
4. Chlorhexidine or emollient application determined inappropriate in the opinion of the enrolling clinician

3.3 NUMBER OF PATIENTS

182 babies will be randomised in total (targeting 91 babies in each site, 7 babies in each stratum per site). Babies without a baseline and two post-baseline swabs will be replaced to maintain power. All baseline and post-baseline results will be included in the analysis.

3.4 CO-ENROLMENT GUIDELINES

Co-enrolment in previous or future trials is considered in **Section 4.3.**

3.5 SCREENING PROCEDURES & PRE-RANDOMISATION INVESTIGATIONS

Written informed consent to enter into the trial and be randomised will be obtained from parents after explanation of the aims, methods, benefits and potential hazards of the trial and BEFORE any

trial-specific procedures are performed. Mothers and families will be approached after the baby is admitted to the neonatal unit, and when initial routine clinical procedures and communication with parents have already been completed. They will be given information on the trial including a patient information leaflet in a language they understand, and those who consent will have a baseline assessment and be randomised. They may take as much time as they need to consider joining the study, but will be made aware that only babies up to 6 days old can join.

It must be made completely and unambiguously clear that the parent or carer is free to refuse to participate in all or any aspect of the trial, at any time and for any reason, without incurring any penalty or affecting the treatment of the baby.

Signed consent will be kept by the investigator and documented in the baby's notes and a copy given to the family. Consent may be given using a thumbprint with signed agreement from a witness.

4 RANDOMISATION

Before randomisation, the baby's eligibility for enrolment will be confirmed (see **Section 3**). Parents/carers must confirm that they have read the relevant patient information sheet and have provided written informed consent to enter into the trial. Enrolment assessments will be performed as summarised in the Trial Assessment Schedule, and the clinician should complete the relevant Enrolment Form which should be data entered directly onto the secure web-based trial database.

A trial register will be kept at the clinical site and will record all babies who are eligible and invited to join the trial. Those accepting will have initials, date of admission, age, randomisation date and trial number recorded. Those who refuse will have initials, date of admission, age and reason for refusal (if provided) recorded. The register will be kept in a secure place in each clinical site; must be available for monitoring, audit and inspection; and will be the responsibility of the Principal Investigator at that site.

4.1 RANDOMISATION PRACTICALITIES

To randomise a baby, the information contained on a completed randomisation case record form (CRF)/worksheet will be entered into the online system which is accessible from the local clinical sites, and will automatically check for eligibility. Delegated member(s) of staff at each site will be responsible for carrying out randomisation, which will be restricted using role-based access. The details of the baby's treatment allocation and NeoCHG trial number will be notified to clinical staff, and the trial number and allocation cross checked between those randomising and those managing the baby clinically.

If the site's internet connection is unavailable at the time of randomisation, the baby's details can be provided to staff at MRC CTU by email or phone (using the contact details at the front of this protocol) during normal UK working hours. At the MRC CTU, staff will verify eligibility and perform the randomisation using the online system. The details of the baby's treatment allocation and NeoCHG trial number will be notified to the trial team at the site by email.

If the main electronic randomisation system is not working, randomisations will not take place, and this should be recorded as the reason that the baby was not randomised.

The baby's trial number and the date of randomisation will be entered into the trial register at the site.

4.2 RANDOMISATION CODES & UNBLINDING

The allocation will not be blinded as this is not practical given the number of factors evaluated. However, the efficacy co-primary endpoint (bacterial colonisation) will be assessed by laboratory staff blinded to randomisation.

4.3 CO-ENROLMENT GUIDELINES AND REPORTING

Co-enrolment in another interventional trial is not allowed for the duration of follow up. Co-enrolment in other non-interventional (i.e. observational) studies may be allowed but should be discussed with the Chief Investigator before enrolment.

5 TREATMENT OF PATIENTS

5.1 INTRODUCTION

All babies will receive routine care as per normal procedures in each site, except for the interventions of the trial including chlorhexidine skin wipes and emollient, and the trial's clinical and microbiological assessments.

The 13 arms of the trial, to which babies will be randomised 1:1:1:1:1:1:1:1:1:1:1:1:1 are as follows:

- Control group without CHG and without emollient

- 0.5% CHG each working day with emollient
- 0.5% CHG each working day without emollient
- 0.5% CHG alternate working days with emollient
- 0.5% CHG alternate working days without emollient

- 1% CHG each working day with emollient
- 1% CHG each working day without emollient
- 1% CHG alternate working days with emollient
- 1% CHG alternate working days without emollient

- 2% CHG each working day with emollient
- 2% CHG each working day without emollient
- 2% CHG alternate working days with emollient
- 2% CHG alternate working days without emollient

Alternate working days are Monday, Wednesday, Friday; or Tuesday, Thursday, Saturday, depending on the standard working pattern in each country. The treating clinician(s) and parents/carers will be unblinded to the allocated treatment. Chlorhexidine will be diluted locally, in the central pharmacy, following the site-specific pharmacy SOPs/guidelines.

5.2 TRIAL TREATMENTS

All babies participating in the trial, except those randomised to the control group, will receive chlorhexidine with or without emollient. Treatment should start immediately following randomisation as described below, following the randomised frequency schedule.

5.2.1 CHLORHEXIDINE

Babies will be randomised to three different concentrations of chlorhexidine by dilution, 0.5%, 1% and 2%, to be applied daily (working days s only) or on alternate working days (three times a week) until day 14 after enrolment or discharge from hospital, whichever is earlier. Each baby will be allocated their own labelled bottle of the diluted product.

Before chlorhexidine is applied, the baby's temperature should be measured and the skin condition score assessment should be performed. Keeping the baby warm, treatment should be applied to the whole body (excluding face and eyes) using cotton wool, avoiding pooling of the chlorhexidine around the baby. After the application, the baby should be re-dressed and their body temperature should be measured after 15 minutes. The baby should be observed for skin reactions at 30 minutes after each application.

In the case of rare and highly unlikely accidents such as splash injury to the eye from chlorhexidine, flush the affected area with copious water or saline as quickly as possible, and consider whether referral for an opthalmological assessment is necessary.

5.2.1.1 Dispensing

Chlorhexidine will be diluted in the hospital pharmacy and transported to the ward in pre-defined concentrations. Containers for individual use will be prepared on the ward and labelled for clinical trial use, adding the trial number and date of birth to each container immediately after randomisation. Individual containers should be stored in a central location until application. These containers should not be shared with any other trial participants or babies outside the trial.

5.2.1.2 Dose Interruptions & Discontinuations

Doses and concentrations will not be modified after the baby has been randomised. Consideration of temporary or permanent discontinuation of chlorhexidine should be made at the discretion of the treating clinician in the following circumstances:

- Hypothermia < 36.0C immediately before chlorhexidine application
- Skin score >2 in any category immediately before chlorhexidine application (see **Appendix I**)
- Neurological disturbance

When chlorhexidine is temporarily stopped for any reason, an assessment will be performed at the time of the next scheduled application and the clinician will determine whether it should be re-started.

Treatment interruptions and discontinuation should be recorded as appropriate on the trial CRFs/worksheets.

5.2.1.3 Stopping Chlorhexidine Early (Permanent Discontinuation)

Stopping chlorhexidine permanently before discharge/day 14 will be decided by the clinician. Possible reasons for permanent discontinuation would include the baby developing neurological symptoms such as focal neurological abnormalities, seizures or encephalopathy.

5.2.1.4 Handling Cases of Overdose

Overdose of chlorhexidine skin application has not been previously defined. For the purposes of this study, overdose will be defined as more than once daily application; any additional doses given will be recorded on the treatment CRF/worksheet. Adverse reactions to this would be expected to be similar to those already described in **Section 7.1.12** and will be recorded and reported in the same way as other adverse reactions (see **Section 7**).

5.2.1.5 Accountability & Unused Products

The diluted chlorhexidine to be used for the trial will be stored separately from routine supplies in a designated section of the pharmacy, the ward or other appropriate location. The product will be designated for strict use for trial participants. The designated trial pharmacist or nurse will confirm receipt of supplies prior to the commencement of the trial. Inventories will be conducted regularly, and logs returned to the main pharmacy as detailed in the MOP. Inventories will be in compliance with applicable local regulation, GCP and the protocol.

On no account should any product assigned to a baby be used for anyone else. Unused trial product must be returned to the main pharmacy if a baby is withdrawn from treatment before completing their randomised duration of therapy or if there is any remaining product.

At each site, a named person (pharmacist or research nurse) will be required to maintain complete records of all trial medication dispensed and returned. The designated pharmacist/nurse will, on receipt of supplies prior to the start of the trial, conduct an inventory and complete a receipt.

Procedures for product distribution, labelling, accountability and destruction will be detailed in the Pharmacy MOP. Product accountability will be regularly monitored and the remaining stocks checked against the amounts dispensed. At the end of the trial, all remaining products will be destroyed.

5.2.1.6 Compliance and Adherence

Each application of chlorhexidine will be given in hospital and recorded in the appropriate CRFs/worksheets.

5.2.2 EMOLLIENT

Sunflower oil will be used as emollient. Babies will be randomised to receive or not receive emollient together with chlorhexidine, to be applied daily or on alternate days (working days only) until day 14 of the trial or discharge from hospital, whichever is earlier.

Emollient should be applied after chlorhexidine is applied as described above. Approximately four grams per kilogram of body weight of emollient will be applied to the body (excluding face, ears and eyes) when the skin has become dry after application of chlorhexidine. After both products have been applied, the baby should be re-dressed and body temperature should be measured after 15 minutes. The baby should be observed for skin reactions at 30 minutes after application.

If chlorhexidine is being omitted for any reason, the emollient should normally still be applied as per protocol, at the discretion of the treating clinician.

5.2.2.1 Dispensing

Emollient will be prepared centrally in pharmacy and aliquoted, in aseptic conditions, into individual containers. Each baby will be allocated their own labelled bottle of emollient and they will not be shared between participants.

5.2.2.2 Dose Interruptions & Discontinuations

Doses will not be modified after the baby has been randomised. Consideration of temporary or permanent discontinuation of emollient should be made at the discretion of the treating clinician.

5.2.2.3 Stopping Emollient Early

If the clinician judges that an adverse reaction is likely to be related to emollient use, rather than to chlorhexidine, then the emollient may be stopped. Otherwise, if it is unclear which product is likely to be related to the adverse reaction, both products will be stopped.

5.2.2.4 Handling Cases of Overdose

Overdose will be defined as more than once daily application of emollient, and will be recorded on the treatment CRF/worksheet. This is unlikely to cause adverse reactions, as more frequent application of sunflower oil has been shown to be safe (e.g. three times daily application in one trial).[26] Any adverse reactions will be recorded as other adverse reactions.

5.2.2.5 Accountability & Unused Emollient

Unused emollient which has already been assigned to a specific individual baby will be discarded without being used for other babies or trial participants. See **Section 5.2.1.5** above for general accountability.

5.2.2.6 Compliance & Adherence

Each application of emollient will be given in hospital and recorded in the CRFs/worksheets.

5.3 CONTROL GROUP

5.3.1 PRODUCTS

The control group will receive routine clinical care. Any skin product which is used will be recorded on CRFs/worksheets, along with the indication for its use.

The control group will receive a daily assessment schedule, including skin score, AE and temperature assessments, as well as swabs being taken on the protocol schedule.

5.3.2 COMPLIANCE & ADHERENCE

If a baby in the control group receives chlorhexidine skin application, this may be due either to inadvertent application of the procedure (e.g. due to error thinking the baby is in an intervention group), or due to routine use of chlorhexidine, for example for an invasive procedure. Both will be recorded on trial CRFs/worksheets. In the case of routine use for a specific purpose related to routine clinical care (for example local application prior to insertion of a vascular device), this will be recorded but not classified as a protocol deviation, whereas if a baby inadvertently receives an application designed for a baby randomised into an intervention group, then this will be recorded as a protocol deviation.

5.4 UNBLINDING / UNMASKING

NeoCHG is an open label trial and therefore there is no unblinding/unmasking.

5.5 PROTOCOL TREATMENT DISCONTINUATION

In consenting to the trial, parents/carers are consenting to trial treatment, trial follow-up and data collection. However, an individual baby may stop treatment early for any of the following reasons:

- Unacceptable toxicity or adverse event
- Intercurrent illness that prevents further treatment
- Any change in the baby's condition that justifies the discontinuation of treatment in the clinician's opinion, including neurological disturbance developing at any point during the trial
- Inadequate compliance with the protocol treatment in the judgement of the treating clinician
- Withdrawal of consent for treatment by the parent/carer

As the baby's participation in the trial is entirely voluntary, the parent/carer may choose to discontinue the baby's trial treatment at any time without penalty or loss of benefits to which they are otherwise entitled. Although the parent/carer is not required to give a reason for discontinuing their trial treatment, a reasonable effort should be made to establish this reason while fully respecting their rights.

Babies should remain in the trial for the purpose of follow-up and data analysis (unless the parent/carer withdraws their consent from all stages of the trial). If a baby is withdrawn from follow-up, refer to **Section 6.5.**

Data already recorded will be kept for babies who stop follow-up early.

5.6 COMPLIANCE & ADHERENCE

Adherence to allocated interventions will be recorded on CRFs/worksheets. Compliance is expected to be high because the allocated intervention is administered in hospital.

5.7 DATA COLLECTION

Baseline, treatment and follow up data including information about all antibiotics and concomitant topical medication received will be recorded on CRFs/worksheets. Other concomitant medications will not be recorded.

5.8 NON-TRIAL TREATMENT

5.8.1 MEDICATIONS PERMITTED

After entry into the trial, any concomitant medication which is required is permitted, including any antimicrobials. Topical treatments and antibiotics will be recorded on CRFs/worksheets.

5.8.2 MEDICATIONS NOT PERMITTED

As an inclusion criteria parents will be asked to agree not to apply routine emollients outside of the trial procedures. However, if they decide to do so, any such emollient use will be recorded on trial CRFs/worksheets but will not be counted as a protocol deviation as it will be done by the parent or carer, outside the control of the trial team. Babies should continue to be followed in the trial, regardless of whether the parent/carer follows the allocated regimen.

5.8.3 MEDICATIONS TO BE USED WITH CAUTION

Chlorhexidine may be required for routine care in any group, such as an invasive procedure. When used outside of the trial procedures this will be recorded in the CRF/worksheet and will not be considered as a protocol deviation if it is for routine care.

5.8.4 TREATMENT AFTER TRIAL EVENT

Treatment will be at the discretion of the responsible clinician.

5.9 CO-ENROLMENT GUIDELINES

Co-enrolment in previous or future trials is considered in **Section 4.3**.

6 ASSESSMENTS & FOLLOW-UP

6.1 TRIAL ASSESSMENT SCHEDULE

The trial assessment schedule is described in **Table 1.**

After screening, baseline data including mode of delivery, birth weight, gestational age, comorbidities, and intrapartum and postnatal antibiotic history will be collected. The skin will be assessed, and the baseline skin score determined.

Swabs will be taken, and temperature will be checked before each application of chlorhexidine +/- emollient. After the product(s) has been applied, the body temperature should be measured after 15 minutes. A further assessment of the skin will be carried out at 30 minutes post application. Babies randomised to the control group will have swabs taken and temperature checked once on the same days as babies randomised to the alternate working day schedule.

Microbiological assessments will be performed with a nose, cervical skin fold and umbilicus (pooled) swab and a separate peri-anal swab. The timing of these swabs is described in **Table 1,** and must be taken before any application of chlorhexidine and emollient.

6.2 PROCEDURES FOR ASSESSING EFFICACY

Efficacy will be measured as change in colony forming units (CFUs) with one swab from the nose, one combined swab (cervical skin folds and umbilicus pooled) and one peri-rectal swab from randomisation (before chlorhexidine application) to day 3+/- 1 day and day 8 +/- 3 days of the trial. Swabs will be vortexed in liquid transport medium, inoculated in serial dilutions on MacConkey and blood agar, and read after 24 hours incubation. Both total bacterial load (total CFU) as well as pathogen specific CFUs will be recorded.

6.3 PROCEDURES FOR ASSESSING SAFETY

Safety will be assessed using a modified skin score which assesses the skin condition, and presence of erythema, dryness, and any other sign of skin damage (see **Appendix I**). This will be performed before each application of chlorhexidine or emollient and 30 minutes after the product(s) application. The skin score will be considered as both an absolute value and graded. Skin score assessments will be performed once every working day on all babies to provide unbiased comparisons across randomised groups. At other times, skin score assessments will be performed as required if there is clinical concern.

In addition, temperature will be measured before, and 15 minutes after application, to monitor for hypothermia. Hypothermia will be graded as follows:

- Grade 1: 35.5 - 36.0C
- Grade 2: 35.4 - 35.0C
- Grade 3: 34.0 - 34.9C
- Grade 4: <34.0C

Other adverse events will be reported as described in **Section 7**.

6.4 OTHER ASSESSMENTS

The baby's weight will be measured on the day of enrolment and the earliest of discharge or day 14.

6.5 EARLY STOPPING OF FOLLOW-UP

If a parent/carer (who gave consent) chooses to discontinue their baby's trial treatment, the baby should always be followed up providing the parents/carers are willing; that is, they should be encouraged to not leave the whole trial. If the parents/carers do not wish their baby to remain on trial follow-up, however, their decision must be respected and the baby will be withdrawn from the trial completely. The MRC CTU should be informed of this in writing via email and early stopping detailed in the clinical notes.

The medical data collected during the baby's participation in the trial will be kept for research and analysis purposes, in a pseudonymised format (identified only by their trial number). Consent for future use of stored samples already collected can be refused when leaving the trial early (but this should be discouraged and should follow a discussion).

Parents may change their minds about stopping trial follow-up at any time and re-consent to participation in the trial.

6.6 PATIENT TRANSFERS

If a baby moves from the trial hospital to another hospital during the trial, further application and assessments related to the trial will not be continued at the other hospital. The 28 day follow-up phone call will still be done to ascertain vital status and will be documented in a telephone follow-up sheet provided by the MRC CTU and filed in the baby's trial file.

6.7 FOLLOW-UP

Follow up will be until 28 days after randomisation, which will be via phone call if the baby has already been discharged. At day 28 the baby's vital status will be ascertained, together with occurrence of serious adverse events (including clinically defined sepsis, necrotising enterocolitis (NEC)) and any unscheduled visits to healthcare providers. Parents/carers will be provided with the trial team's details at discharge and will be told to bring their baby back to the hospital if they have any concerns.

6.8 COMPLETION OF PROTOCOL FOLLOW UP

Each baby will complete follow-up in the trial at their day 28 visit.

7 SAFETY REPORTING

The principles of GCP require that both investigators and Sponsors follow specific procedures when notifying and reporting adverse events or reactions in clinical trials. These procedures are described in this section of the protocol. **Section 7.1** lists definitions, **Section 7.2** gives details of the investigator responsibilities and **Section 7.4** provides information on the MRC CTU responsibilities.

7.1 DEFINITIONS

The definitions of the EU Directive 2001/20/EC Article 2 based on the principles of GCP, which is in line ICH E2A apply to this trial protocol. These definitions are given in **Table 1**.

Table 1 Definitions

TERM	DEFINITION
Adverse Event (AE)	Any untoward medical occurrence in a patient or clinical trial subject to whom a medicinal product has been administered including occurrences that are not necessarily caused by or related to that product.
Adverse Reaction (AR)	Any untoward and unintended response to an investigational medicinal product related to any dose administered.
Unexpected Adverse Reaction (UAR)	An adverse reaction, the nature or severity of which is not consistent with the information about the medicinal product in question set out in the Summary of Product Characteristics (SmPC) or Investigator Brochure for that product.
Serious Adverse Event (SAE) or Serious Adverse Reaction (SAR) or Suspected Unexpected Serious Adverse Reaction (SUSAR)	Respectively any adverse event, adverse reaction or unexpected adverse reaction that:<br>▪ Results in death<br>▪ Is life-threatening*<br>▪ Requires hospitalisation or prolongation of existing hospitalisation**<br>▪ Results in persistent or significant disability or incapacity<br>▪ Consists of a congenital anomaly or birth defect<br>▪ Is another important medical condition***

*The term life-threatening in the definition of a serious event refers to an event in which the patient is at risk of death at the time of the event; it does not refer to an event that hypothetically might cause death if it were more severe, for example, a silent myocardial infarction.

**Hospitalisation is defined as an inpatient admission, regardless of length of stay, even if the hospitalisation is a precautionary measure for continued observation. Hospitalisations for a pre-existing condition, that has not worsened or for an elective procedure do not constitute an SAE.

*** Medical judgement should be exercised in deciding whether an AE or AR is serious in other situations. The following should also be considered serious: important AEs or ARs that are not immediately life-threatening or do not result in death or hospitalisation but may jeopardise the subject or may require intervention to prevent one of the other outcomes listed in the definition above; for example, a secondary malignancy, an allergic bronchospasm requiring intensive emergency treatment, seizures or blood dyscrasias that do not result in hospitalisation or development of drug dependency.

7.1.1 PRODUCT

The trial is testing chlorhexidine and emollient for topical use.

7.1.2 ADVERSE EVENTS

Adverse Events include:

- An exacerbation of a pre-existing illness
- An increase in frequency or intensity of a pre-existing episodic event or condition
- A condition (even though it may have been present prior to the start of the trial) detected after trial product administration
- Continuous persistent disease or a symptom present at baseline that worsens following administration of the trial treatment

Assessment of adverse events (AEs) is challenging in neonates since standard grading scales do not apply; for example, normal ranges of laboratory parameters vary enormously with age and normal function is difficult to specify.

Adverse events will therefore be assessed using an adapted version of the DAIDS scale for neonates, that has been used in previous neonatal trials by groups such as IMPAACT,[38] including studies conducted for regulatory FDA approval (**Appendix II**).

Each adverse event will be assessed at each scheduled visit and graded by the investigator according to the Adapted DAIDS score for neonates, reproduced from the IMPAACT network[38] (**Appendix II**).

Skin damage and hypothermia will both be separately assessed as part of primary and secondary outcomes and graded according to the classification shown in **Appendix I** and **Section 6.3.**

7.1.3 SAFETY MANAGEMENT

In the case of adverse events, standard operating procedures will be available for anticipated events including hypothermia, skin damage, as well as rare and highly unlikely accidents such as splash injury to the eye from chlorhexidine.

7.1.4 EXEMPTED ADVERSE EVENTS

Adverse Events do not include:

- Medical or surgical procedures; the condition that leads to the procedure is the adverse event
- Pre-existing disease or a condition present before treatment that does not worsen
- Hospitalisations where no untoward or unintended response has occurred, eg, social admissions
- Overdose of medication without signs or symptoms

7.2 INVESTIGATOR RESPONSIBILITIES

Presence of non-serious AEs in **Appendix II** will be documented in CRFs/worksheets at each scheduled visit.

All AEs meeting the seriousness criteria defined in **Table 2,** including but not limited to those related to skin damage, hypothermia or neurological disturbance should be notified to CTU using the SAE Form within 24 hours of the investigator becoming aware of the event.

7.3 INVESTIGATOR ASSESSMENT

7.3.1 SERIOUSNESS

When an AE occurs, the investigator responsible for the care of the baby must first assess whether or not the event is serious using the definition given in **Table 2**. If the event is serious, then an SAE Form must be completed and the MRC CTU notified within 24 hours of the investigator becoming aware of the event.

7.3.2 SEVERITY OR GRADING OF ADVERSE EVENTS

AEs, serious or not serious, should be graded using the Adapted DAIDS score for neonates, reproduced from the IMPAACT network[38] (**Appendix II**).

7.3.3 CAUSALITY

The investigator must assess the causality of all serious events in relation to the trial treatment using the definitions in **Table 3**. There are five categories: unrelated, unlikely, possible, probable, and definitely related. If the causality assessment is unrelated or unlikely to be related, the event is classified as an SAE. If the causality is assessed as possible, probable or definitely related, then the event is classified as an SAR.

Table 2 Assigning Type of SAE Through Causality

RELATIONSHIP	DESCRIPTION	SAE TYPE
Definitely	There is clear evidence to suggest a causal relationship and other possible contributing factors can be ruled out.	SAR
Probably	There is evidence to suggest a causal relationship and the influence of other factors is unlikely.	SAR
Possibly	There is some evidence to suggest a causal relationship (for example, because the event occurs within a reasonable time after administration of the trial medication). However, the influence of other factors may have contributed to the event (for example, the patient's clinical condition, other concomitant treatments).	SAR
Unlikely	There is little evidence to suggest that there is a causal relationship (for example, the event did not occur within a reasonable time after administration of the trial medication). There is another reasonable explanation for the event (for example, the patient's clinical condition, other concomitant treatment).	Unrelated SAE
Unrelated	There is no evidence of any causal relationship	Unrelated SAE

If an SAE is considered to be related to trial treatment and chlorhexidine or emollient is stopped, refer to **Sections 5.2.1.2** and **5.2.2.2** for more information on dose interruptions and discontinuations.

7.3.4 NOTIFICATION

The MRC CTU should be notified of all SAEs within 24 hours of the investigator becoming aware of the event. Investigators should notify the MRC CTU of all SAEs occurring from the time of signature of the informed consent form until 28 days after randomisation.

7.3.5 Notification Procedure

The SAE Form must be completed by an investigator (the clinician, named on the Signature List and Delegation of Responsibilities Log, who is responsible for the baby's care; this will be either the Principal Investigator or another medically qualified person with delegated authority for SAE reporting). Due care should be paid to the grading, and assessment of causality of the event, as outlined above. In the absence of the responsible investigator, the form should be completed and signed by a member of the site trial team and emailed. The responsible investigator should subsequently check the SAE Form, make changes as appropriate, sign and then re-email to the MRC CTU as soon as possible. The initial report must be followed by detailed, written reports as appropriate.

The minimum criteria required for reporting an SAE are the trial number and date of birth, name of investigator reporting, the event, and why it is considered serious.

The SAE Form must be sent by encrypted email to the MRC CTU (see below).

Follow-up: babies must be followed up until clinical recovery is complete and laboratory results have returned to normal or baseline, or until the event has stabilised. Follow-up should continue after completion of protocol treatment if necessary. A further SAE Form, indicated as 'Follow-up SAE Form' should be completed and emailed to the MRC CTU as information becomes available (or where a small amount of fields are updated, the original SAE form may be modified following GCP). Extra, annotated information and/or copies of test results may be provided separately. The baby must be identified by trial number and date of birth. The baby's name must not be used on any correspondence and should be deleted from any test results.

Staff should follow their institution's procedure for local notification requirements.

SAE REPORTING
Within 24 hours of becoming aware of an SAE, please email a
completed SAE form to the MRC CTU on **mrcctu.neochg@ucl.ac.uk**

7.4 MRC CTU RESPONSIBILITIES

Medically-qualified staff at the MRC CTU and/or the Chief Investigator (or a medically-qualified delegate) will review all SAE reports received and assess expectedness as necessary. The causality assessment given by the local investigator at the hospital cannot be overruled; in the case of disagreement, both opinions will be provided in any subsequent reports.

The MRC CTU is delegated by the Sponsor for the reporting of SUSARs and other SARs to the regulatory authorities and the research ethics committees, as appropriate.

8 QUALITY ASSURANCE & CONTROL

8.1 RISK ASSESSMENT

The Quality Assurance (QA) and Quality Control (QC) considerations have been based on a formal Risk Assessment, which acknowledges the risks associated with the conduct of the trial and how to address them with QA and QC processes. QA includes all the planned and systematic actions established to ensure the trial is performed and data generated, documented and/or recorded and reported in compliance with the principles of GCP and applicable regulatory requirements. QC includes the operational techniques and activities done within the QA system to verify that the requirements for quality of the trial-related activities are fulfilled. This Risk Assessment has been reviewed by the MRC CTU's Research Governance Committee (RGC) and has led to the development of a Data Management Plan (DMP), Safety Reporting Plan and Monitoring Plan which will be separately reviewed by the Quality Management Advisory Group (QMAG).

8.2 CENTRAL MONITORING AT THE MRC CTU

Each site will be responsible for its own data entry and local trial management. Data will be entered into the online trial database directly at the site. The site will retain the original CRFs/worksheets. Data stored on the central database will be checked at the MRC CTU for missing or unusual values (range checks) and checked for consistency within neonates over time. If any problems relating to data quality are identified, the site will be contacted and asked to verify or correct the entry. Changes will be made on the original CRF/worksheet and entered into the database at the site. The MRC CTU will also send reminders for any overdue and/or missing data with the regular inconsistency reports of errors.

Other essential trial issues, events and outputs will be detailed in the Monitoring Plan that is based on the trial-specific Risk Assessment.

8.3 ON-SITE MONITORING

The frequency, type and intensity for routine monitoring and the requirements for triggered monitoring will be detailed in the Monitoring Plan. This plan will also detail the procedures for review and sign-off.

A detailed site initiation meeting with training will be performed at each trial site by staff from the MRC CTU either face to face or online. The site initiation meeting will include training in the administration and side effects of study products, as well as the trial procedures.

8.3.1 Direct Access to Patient Records

Participating investigators should agree to allow trial-related monitoring, including audits, ethics committee review and regulatory inspections by providing direct access to source data and documents as required. Parental/caregiver consent for this must be obtained. Such information will be treated as strictly confidential and will in no circumstances be made publicly available.

The trial data and consent/assent should all be verifiable from source documents which may include paper notes and electronic health records.

Not all such information will be monitored; rather the monitoring plan will describe a risk-based approach to monitoring based on ongoing random samples of clinical and laboratory data, which may be increased if issues are identified.

The investigator/institution should maintain adequate and accurate source documents and trial records that include all pertinent observations on each of the site's trial subjects. Source data should be attributable, legible, contemporaneous, original, accurate, and complete. Changes to source data should be traceable, should not obscure the original entry, and should be explained if necessary (e.g., via an audit trail).

8.3.2 CONFIDENTIALITY

We plan to follow the principles of the EU General Data Protection Regulation (GDPR) as well as being compliant with national data laws in the countries where the trial is being conducted. In particular, the investigator must ensure that baby's anonymity will be maintained and that their identity is protected from unauthorised parties. Babies will be assigned a trial identification number and this will be used on CRFs/worksheets; they will not be identified by name. The investigator will keep securely a trial register showing trial identification numbers, initials and date of admission and age at admission, held only at the local site. The unique trial number (or laboratory tracking number) will identify all laboratory specimens, CRFs/worksheets, and other records and no names or initials will be used on forms or samples, in order to maintain confidentiality. Date of birth will be collected as a data item since all babies will be under 28 days old and hence is needed to calculate age precisely. All records will be kept in locked locations. Clinical information will not be released without written permission, except as necessary for monitoring by the trial monitors.

9 STATISTICAL CONSIDERATIONS

9.1 METHOD OF RANDOMISATION

Randomisation will be online with allocation concealment. The design will not be blinded as this is not practical given the factors evaluated. However, the efficacy co-primary endpoint (bacterial colonisation) will be assessed by laboratory staff blinded to randomisation.

9.2 OUTCOME MEASURES

9.2.1 CO-PRIMARY OUTCOME MEASURES

- Skin bacterial load – change in colony forming units (CFUs) in the nose (1 swab), cervical skin folds and umbilicus (1 pooled swab), and peri-rectal area (1 swab) from randomisation (before chlorhexidine application) to D3+/- 1 day and D8 +/- 3 days microbiology data collection (efficacy).

- Modified neonatal skin condition score (see **Appendix I**) used before each application of chlorhexidine, or alternate working days in controls (safety). The primary analysis of this outcome will consider the absolute score. Secondary analysis of this outcome will consider graded toxicity (see **Appendix I**).

9.2.2 SECONDARY OUTCOME MEASURES

- Temperature after chlorhexidine/emollient: considered both as change from before application to after application (after swabs taken in controls), change from baseline after randomisation, and as graded toxicity
- Acquisition and loss of specific bacterial species: *Enterobacteriaceae, Acinetobacter*, *Staphylococcus aureus*, Group b haemolytic streptococci (A and B), Enterococcus, Candida
- SAEs

9.3 SAMPLE SIZE

The trial is designed as a permuted block factorial, with an additional control per permuted block. 182 neonates in 14 permuted blocks provides 90% power to detect a difference of 0.66 standard deviations (SDs) between concentrations and 0.47 SDs between the different levels of frequency and emollient (two-sided α=0.05) (80% power for 0.58 and 0.41 SDs, respectively). This also provides 90% power to detect a difference of 1.04 SD between each concentration and control, and 0.96 SD between each level of frequency/emollient and control (0.92 and 0.96 SDs at 80% power). Previous studies have found varying effects of chlorhexidine on log colony counts: a decrease from baseline to 24h of 0.2SD with 1% chlorhexidine in Nepal and 2SDs with 2% chlorhexidine in the USA.[18,21]

9.4 INTERIM MONITORING & ANALYSES

A DMC Charter will be drawn up that describes the membership of the DMC, terms of reference, decision-making processes, and the timing and frequency of meetings. The DMC will meet around the time the trial starts (without reviewing any data) to ensure they are familiar with its design and conduct. They will formally review data halfway through planned recruitment. They may call

additional meetings at any time at their discretion. In addition, SARs will be sent in real time during the trial to the DMC.

The DMC can recommend premature closure or reporting of the trial, or that recruitment to any randomised group be discontinued or modified. Such recommendations would be made if, in the view of the DMC, there is an unacceptable rate of adverse reactions. There will be no early stopping for efficacy because this pilot trial is powered only to detect differences in skin bacterial load, not clinical efficacy outcomes. See **Section 13.2** for details on DMC membership.

9.5 ANALYSIS PLAN (BRIEF)

The analyses will be described in detail in a full Statistical Analysis Plan. This section summarises the main issues.

The primary analysis population is intention-to-treat, including all randomised babies, regardless of treatment received. This corresponds to estimating the impact of the effectiveness of the treatments. However, in secondary analyses we will also use inverse-probability weighting methods to adjust for deviation from randomised strategy, which is a more efficient approach than defining a per-protocol population.[39] Loss-to-follow up is expected to be low given that expected length of hospital stay under 7 days is an exclusion criteria. Therefore, most babies should have baseline and two subsequent swabs on their allocated regimen. Missing swabs will be monitored and babies without baseline and two post-baseline swabs will be replaced to maintain power. However, all baseline and post-baseline results will be included in analyses.

Analysis of the co-primary endpoints will compare within-individual differences over time across randomised arm to maximise the information gained from the trial. Analysis will use linear mixed-models, with individual as a random effect, will adjust for baseline values of each outcome, and use Normally distributed errors. Analysis will be both frequentist, enabling straightforward comparison between arms (primary analysis since sample size is based on this), and Bayesian, enabling estimation of the probability that one arm is truly superior to another. This is more powerful when sample sizes are small as use of priors provides additional information. Sensitivity analysis to prior assumptions will be performed using optimistic, non-informative and sceptical priors. Change from baseline to the single closest sample 48h post randomisation will also be done as a sensitivity analysis.

The effect of each treatment arm will be assessed within a single model with each factor being fitted independently as a factor, and not as a continuous variable. Comparison of main effects within arms will be assessed using the estimate and 95% confidence interval of the comparison of differences between factors and in comparison, to control. As this is a pilot trial, the sample size was not powered to robustly detect interactions between chlorhexidine concentrations/frequencies and emollients and so we will not test these formally, but will use exploratory analyses to assess whether there is any evidence supporting individual interactions testing each one, one at a time. As this is a pilot trial and the two co-primary outcomes capture different aspects (efficacy and safety) there will be no adjustment for multiple testing, nor for the two different bacterial load outcomes since we expect a reduction in colonisation on the skin, but potentially no change in colonisation peri-rectally.

Similar mixed models will be used to analyse change in skin temperature after vs before each application, including individual as a random effect, the change in temperature as the outcome and the pre-application (baseline) temperature as a factor. Acquisition and loss of each species will be analysed as a binary outcome variable, compared with baseline, in the relevant population (those without the species of interest at baseline, or with the species of interest at baseline, respectively).

Presence/absence of each species at each assessment will also be compared between randomised groups using mixed models, again with individual as a random effect, and with baseline presence as a factor.

Frequency of SAEs will be tabulated by randomised group, and compared using exact logistic models (or time-to-event models if AEs occur in >10% of the trial population overall). SAEs will also be tabulated by grade. Similar analyses will be conducted for the AEs in **Appendix II**.

A Statistical Analysis Plan will be written and approved by the Trial Management Group (TMG) and the DMC before the first interim analysis is reviewed by the DMC.

10 REGULATORY & ETHICAL ISSUES

10.1 COMPLIANCE

10.1.1 REGULATORY COMPLIANCE

The trial complies with the principles of the 1996 version of the Declaration of Helsinki.

It will also be conducted in compliance with the approved protocol, the principles of ICH Good Clinical Practice (GCP) as laid down by the ICH topic E6 (R2).

10.1.2 SITE COMPLIANCE

The site will comply with the above and also with applicable national regulations. An agreement will be in place between the site and SGUL (Sponsor), setting out respective roles and responsibilities (see **Section 13 - Finance**).

The site will inform the MRC CTU and SGUL as soon as they are aware of a possible serious breach of compliance, so that they can report this breach if necessary as per relevant regulatory requirements. For the purposes of this regulation, a 'serious breach' is one that is likely to affect to a significant degree:

- The safety or physical or mental integrity of the subjects in the trial, or
- The scientific value of the trial

10.1.3 DATA COLLECTION & RETENTION

CRFs/worksheets, clinical notes and administrative documentation should be kept in a secure location (for example, locked filing cabinets in a room with restricted access) and held for a minimum of 25 years after the end of the trial. During this period, all data should be accessible, with suitable notice, to the competent or equivalent authorities, the Sponsor, and other relevant parties in accordance with the applicable regulations. The data may be subject to an audit by the competent authorities. Medical files of trial participants should be retained in accordance with the maximum period of time permitted by the hospital, institution or private practice.

10.2 ETHICAL CONDUCT

10.2.1 ETHICAL CONSIDERATIONS

The main ethical concern in this trial relates to potential skin damage, which could lead to infection, and potentially to scarring of the skin. Chlorhexidine is widely used for skin disinfection in neonates. The main risk is with high concentrations (>2%) in extremely low birth weight/preterm neonates (<1kg/<28 weeks), among whom skin reactions have been observed, particularly when chlorhexidine is combined with alcohol. The MHRA accordingly warns about use of high concentrations in this group. Hypothermia has also been observed after skin cleansing with chlorhexidine on the first day of life. This trial will exclude neonates less than 1kg at enrolment and below 28 weeks' gestational age, and start application 1-6 days after birth, which will minimise, although not entirely eradicate these risks. However, close and regular monitoring of the skin with a standardised skin condition scoring system, and temperature checking after application will allow chlorhexidine to be discontinued at the discretion of the local clinician.

Neurotoxicity, which is a theoretical concern, has never been reported in neonates despite widespread use of chlorhexidine, and large studies including daily application of chlorhexidine 4% to the cord, which has been associated with higher blood levels of chlorhexidine than skin application.

Given the absence of known toxicity even in the context of detectable blood chlorhexidine levels, this trial will follow other previous large studies in not measuring blood levels, but will assess neonates for any clinical signs of neurological disturbance (see **Appendix II**).

Importantly, safety concerns are one the main justifications for this pilot, in order to identify the safest but most effective regimen which could be tested in a larger trial aiming to reduce neonatal sepsis and mortality.

Confidentiality of the babies and their parents/care-givers will be maintained throughout the trial. Data submitted on CRFs/worksheets to trial sites and CTU, will be identified only by the trial number and date of birth (including random check letters to improve accuracy of identification). Date of birth is essential given all participants will be under 1 month of age.

Parents and/or carers will be informed fully of known risks and possible benefits by means of a patient information sheet, and this will be reinforced by discussions with the trial research teams at the individual sites prior to enrolment.

For the staff involved in the trial, any risk is expected to be minimal and related to skin irritation with chlorhexidine, or in very rare cases (<1/10,000 as per summary product characteristics), allergic responses to chlorhexidine. Staff with a previous history of sensitivity to chlorhexidine will not take part in application of chlorhexidine.

10.2.2 ETHICAL APPROVALS

Before initiation of the trial at clinical sites, the protocol, all informed consent forms, and information materials to be given to the families will be submitted to an ethics committee for approval. Any further amendments will be submitted and approved by the ethics committee.

The rights of the parent/carer to refuse for their baby to participate in the trial without giving a reason must be respected. After the baby has entered into the trial, the clinician remains free to give alternative treatment to that specified in the protocol, at any stage, if he/she feels it to be in the best interest of the baby. The reason for doing so, however, should be recorded; the baby will remain within the trial for the purpose of follow-up and for data analysis by the treatment regimen to which they were randomised.

10.3 TRIAL CLOSURE

End of trial is defined as 12 months after the last scheduled follow-up visit of the last randomised baby. This is to ensure sufficient time for data submission, data cleaning, verification of queries, database lock and final analysis. Each site will be closed once data cleaning is completed at that site, and the ethics committee and any relevant regulatory authorities will be informed.

11 INDEMNITY

The Sponsor of the trial is St George's, University of London. In consideration of the agreement by the Principal Investigator at each site to supervise the trial, the sponsor undertakes to indemnify the Principal Investigator at each site and the institutions which participate in the trial and their employees and agents in respect of any claims made against them by any third party which arises out of or as a result of the supervision or conduct of the trial (including any claim arising in respect of the technical procedures described in the protocol which babies would not have been exposed to but for their participation in the trial). Full details of the Indemnity agreement are given in a separate document. Cover against claims arising from medical negligence is not included.

12 FINANCE

The NeoCHG trial is funded by the MRC/NIHR/DfID/Wellcome Joint Global Health Trials Call 9 – Trial Development Grant. The Medical Research Council also provides core support to the MRC CTU.

13 OVERSIGHT & TRIAL COMMITTEES

There are several groups involved with the oversight of the trial, detailed below.

13.1 TRIAL MANAGEMENT GROUP (TMG)

A Trial Management Group (TMG) will be formed comprising the Chief Investigator, other lead investigators (clinical and non-clinical) and members of the MRC CTU. The TMG will be responsible for the day-to-day running and management of the trial. It will meet approximately every other month. The full details can be found in the TMG Charter.

13.2 INDEPENDENT DATA MONITORING COMMITTEE (DMC)

An independent Data Monitoring Committee (DMC) will be formed. The Chair will be Prof Gary Damstadt (Stanford University) and the independent member will be Prof Jay Berkley (KEMRI Wellcome Trusts, Kilifi, Kenya). The DMC will be the only group who sees the confidential, accumulating data for the trial. Reports to the DMC will be produced by the MRC CTU statistician(s). The DMC will meet at the beginning of trial to become familiar with the protocol, then approximately halfway through expected recruitment. The DMC will consider data using the statistical analysis plan (see **Section 9.5**) and will advise the TMG. The DMC can recommend premature closure or reporting of the trial, or that recruitment to any randomised arm be discontinued.

Further details of DMC functioning, and the procedures for interim analysis and monitoring are provided in the DMC Charter.

13.3 ROLE OF STUDY SPONSOR

St George's, University of London, is the Sponsor and delegates this responsibility to the MRC CTU at UCL to oversee the implementation of the trial by ensuring that arrangements are put into place for adequate management, monitoring, analysis and reporting of the trial.

14 PATIENT AND PUBLIC INVOLVEMENT

There are no existing patient and public involvement (PPI) groups for premature babies or those admitted to neonatal units at participating sites. Furthermore, this is a sensitive area given the acutely sick nature of babies admitted to these units and the high risks of poor outcomes. Within this pilot trial, there is no specific resource to set up formal patient and public involvement, but we will explore with the parents/carers of babies recruited into this pilot trial their potential interest in being involved in future engagement around a larger trial, particularly in terms of the potential acceptability of the intervention, and differing cultural practices around use of skin products and emollients in premature newborn babies. Therefore, this pilot trial will assess the feasibility of establishing parent/carer groups and identifying possible parent/carer representatives for a future larger trial.

15 PUBLICATION AND DISSEMINATION OF RESULTS

The TMG is responsible for access to the data and bacterial isolates generated from the NeoCHG trial; NeoCHG trial data are not the property of individual participating investigators or healthcare facilities where the data were generated.

- Publications include papers (including abstracts) for presentation at national and international meetings, as well as the preparation of manuscripts for peer-reviewed publication. In order to avoid disputes regarding authorship, it is important to establish a consensus approach that will provide a framework for all publications derived in full or in part from this clinical trial. The following approach is derived from the Lancet and from the publication policies used in other clinical trials coordinated by the MRC CTU at UCL.

- All publications are to be approved by the TMG before submission for publication. Any publication arising before the end of the trial (not by randomised groups) will also be approved by the DMC in order to ensure that the primary objective of the trial (the randomised comparison) is not compromised. The TMG will resolve problems of authorship and maintain the quality of publications.

- In line with MRC policy that the results of publicly-funded research should be freely available, manuscripts arising from the trial will, wherever possible, be submitted to peer-reviewed journals which enable Open Access via UK PubMed Central (PMC) within six months of the official date of final publication.

- For all publications, the TMG will nominate a chairperson or approve an individual’s request to chair a manuscript writing committee. The chair will usually be the primary or senior author. The chairperson is responsible for identifying fellow authors and for determining with that group the order of authorship that will appear on the manuscript. The TMG will resolve any problems of authorship and maintain the quality of publications.

- The TMG will maintain a list of investigators to be presented in an appendix at the end of the paper. This list will include investigators who contributed to the investigation being reported but who are not members of the writing committee, together with all relevant expert advisors and members of the DMC. All families who participated in the trial will be thanked as a group (not by name). In principle, sub-study reports should include all investigators for the main trial, although in some instances where a smaller number of investigators have made any form of contribution, it may be appropriate to abbreviate the listing. All headline authors in any publication arising from the main trial or sub-studies must have a made a substantive academic or project management contribution to the work that is being presented. “Substantive” must be defined by a written declaration of exactly what the contribution of any individual is believed to have been. In addition to fulfilling the criteria based on contribution, additional features that will be considered in selecting an authorship group will include the recruitment of babies who contributed data to any set of analyses contained in the manuscript and/or the conduct of analyses (laboratory and statistical), leadership and coordination of the project in the absence of a clear academic contribution.

- The data derived from this clinical trial are considered the property of the Sponsor. The presentation or publication of any data collected by the participating investigators on

babies entered into this trial is under the direct control of the TMG (and the DMC before the end of the trial). This is true whether the publication or presentation is concerned directly with the results of the trial or is associated with the trial in some other way. However, although individual participating investigators will not have any inherent right to perform analyses or interpretations or to make public presentations or seek publication of any of the data other than under the auspices of and with the approval of the TMG (and the DMC before the end of the trial), they will be encouraged to develop sub-studies or propose analyses subject to the approval by the TMG (and the DMC before the end of the trial). Any requests for access to raw data will be welcomed as long as they are scientifically valid and do not conflict with the integrity of the trial or ongoing analyses by the trial team.

Outcome data by randomised group will not be revealed to the participating investigators until the data collection phase and primary full analysis of the trial has been completed. This policy safeguards against possible bias affecting the data collection. The DMC will be monitoring the outcome results and may recommend that the trial be stopped for safety reasons.

16 DATA AND/OR SAMPLE SHARING

Data will be shared according to the MRC CTU's controlled access approach, based on the following principles:

- No data should be released that would compromise an ongoing trial or study.
- There must be a strong scientific or other legitimate rationale for the data to be used for the requested purpose.
- Investigators who have invested time and effort into developing a trial or study should have a period of exclusivity in which to pursue their aims with the data, before key trial data are made available to other researchers.
- The resources required to process requests should not be under-estimated, particularly successful requests which lead to preparing data for release. Therefore, adequate resources must be available in order to comply in a timely manner or at all, and the scientific aims of the study must justify the use of such resources.
- Data exchange complies with Information Governance and Data Security Policies in all of the relevant countries.

Data will be available for sharing after the trial has reported the primary outcome. Researchers wishing to access trial data should contact the TMG in the first instance.

17 PROTOCOL AMENDMENTS

Not applicable.

APPENDIX I SKIN SCORE ASSESSMENT

Skin toxicity score

Adapted from Lund & Osborne.[31] The skin should be scored on each of the three domains (dryness, erythema and skin breakdown), and then the total score is the sum of the three components (minimum score 0, maximum score 12).

Highest grade from:
Grade 1 = Score of ≥1 in ≥2 categories
Grade 2 = Score of ≥2 in ≥2 categories
Grade 3 = Score of ≥3 in ≥2 categories
Grade 4 = Score of ≥4 in ≥2 categories

Score	Description
**Domain 1**	**Dryness**
0	Normal
1	Dry skin +/- scaling <50%
2	Dry skin + scaling >50% +/- cracking/fissures <50%
3	Very dry + cracking / fissures >50%
4	Very dry + cracking / fissures >75%
**Domain 2**	**Erythema**
0	Normal
1	Erythema <25%
2	Erythema 25-50%
3	Severe Erythema 50-75%
4	Severe Erythema >75%
**Domain 3**	**Skin breakdown**
0	Normal
1	Skin breakdown/ulceration/vesicles <5%
2	Skin breakdown/ulceration/vesicles 5-25%
3	Skin breakdown/ulceration/vesicles 25-50%
4	Skin breakdown/ulceration/vesicles >50%

Figure 3: Skin Affected Area assessment
Modified Lund and Browder chart for estimation of BSA burn involvement in infants and children. (From Harwood-Nuss A, Wolfson A, Linden C. *The clinical practice of emergency medicine*. Philadelphia: Lippincott-Raven; 1996:1207[40]

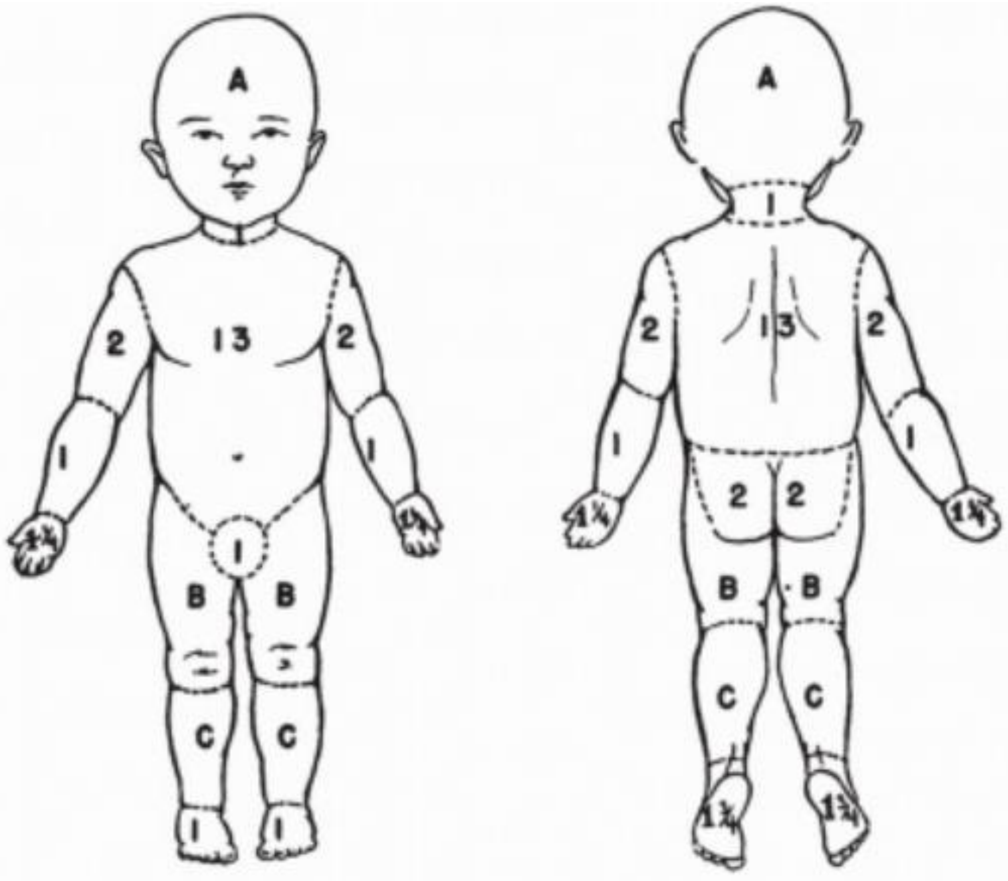

RELATIVE PERCENTAGES OF AREAS AFFECTED BY GROWTH

Area	Age 0	1	5
A = ½ of Head	9½	8½	6½
B = ½ of One Thigh	2¾	3¼	4
C = ½ of One Leg	2½	2½	2¾

% BURN BY AREAS

Probable 3rd° Burn { Head____ Neck____ Body____ Up. Arm____ Forearm____ Hands____
Genitals____ Buttocks____ Thighs____ Legs____ Feet____

Total Burn { Head____ Neck____ Body____ Up. Arm____ Forearm____ Hands____
Genitals____ Buttocks____ Thighs____ Legs____ Feet____

Sum of All Areas____ Probably 3rd°____ Total Burn____

APPENDIX II ADVERSE EVENT GRADING

Adapted DAIDs score for neonates, reproduced from the IMPAACT study.[38]

Parameter	Grade 0 Normal	Grade 1 Mild	Grade 2 Moderate	Grade 3 Severe	Grade 4 Potentially Life-Threatening
Apnea*<br><br>*Apnea spell defined as apnea event >20s or associated with bradycardia, hypoxia or cyanosis	No apneas	< 6 spells* per day	6<12 spells* per day or nasal cannula for apnea	12 or more spells* per day or nasal continuous positive airway pressure (NCPAP) for apnea	Requires intubation for apnea
Anemia	Hb > 10	Hgb 8-10 g/dL	Hgb ≤ 8 g/dL	Requires packed red cell transfusion, no clinical signs	Requires packed red cell transfusion, clinical signs of shock
Congenital Anomalies	None	Minor (no impairment of function)	Minor (no impairment of function), future treatment may be needed	Major (impairment of function), no immediate treatment needed	Major (impairment of function), immediate treatment needed
Congenital Heart Disease	No congenital heart disease	Minor (no impairment of function), no treatment needed	Minor (no impairment of function), future treatment may be needed	Major (impairment of function), no immediate treatment needed	Major (impairment of function), immediate treatment needed
Elecotrolyte/ Metabolic Disorders	None	------------------	Electrolyte/Metabolic disorder, no systemic signs	-----------------------	Electrolyte/Metabolic disorder with systemic signs
GI dysfunction [including necrotizing entercolitis(NEC)]	None	Not ill, abnormal abdominal exam, no treatment needed (e.g., abdominal distension but not NPO)	Mild illness, NPO <3 days (e.g., R/O necrotizing entercolitis (NEC) evaluation, Stage I NEC)	Moderate illness, NPO 3-7 days, or medical treatment (e.g., Stage II NEC)	Severe illness, NPO>7 days, or surgical treatment needed (e.g., Stage III NEC)
Hypertension	No BP performed	Systolic BP> 80-100, no treatment	Systemic BP > 100, no treatment	Treated with one agent	Treatment with multiple agents
Hypotension	No BP performed	Mild clinical signs, no treatment needed	Symptomatic, treated with IV fluids	Symptomatic, treated with single medication	Clinical signs of shock or requiring use of multiple medications
Intraventricular Hemmorhage	No abnormality noted or not assessed	Germinal matrix hemorrhage	Blood in ventricle, no enlargement	Blood in ventricle, with ventricular enlargement	Parenchymal hemorrhage and/or need for ventricular drainage
Jaundice	No jaundice	Mild jaundice, no treatment	Phototherapy and/or IVIG	Exchange transfusion	Acute bilirubin encephalopathy
Neonatal Abstinence Syndrome (NAS)	No history of abstinence syndrome	NAS signs, no medical treatment	NAS controlled with single drug	NAS controlled with two drugs	NAS with seizures
Neurologic compromise	Normal neurological examinations	Mildly abnormal neurologic exam, no clinical or EEG seizure activity	Stage I encephalopathy or possible clinical or EEG seizure activity but no treatment	Stage II encephalopathy or single drug seizure therapy	Stage III encephalopathy or multiple drug seizure therapy
Neutropenia	ANC> 1000/mm3	ANC<1000/mm^3	ANC<500/mm^3	Treated with GCSF	WBC transfusion
Patent Ductus Arteriosus	No murmur	Clinical signs, no treatment	Treatment with fluid restriction or diuretics	Treatment with indomethacin or ibuprofen	Surgical ligation
Persistent Pulmonary Hypertension	No difference in pre- and	Supplemental O_2 but no mechanical ventilation	Conventional ventilator < 5 days	Conventional ventilation 5-10 days, alternative ventilation (e.g., high frequency	Mechanical ventilation > 10 days

Parameter	Grade 0 Normal	Grade 1 Mild	Grade 2 Moderate	Grade 3 Severe	Grade 4 Potentially Life-Threatening
	post-ductal difference			oscillatory ventilation (HFOV), sildenafil and/or nitric oxide	
Renal Dysfunction	Wet diapers documente d	Urine output 1 < 1.5 mL/kg/hr	Urine output 0.5 <1.0 mL/kg/hr	Urine output 0 < 0.5 mL/kg/hr	Prolonged anuria
Respiratory Insufficiency	Baby is in room air – no supplement al oxygen	Nasal cannula oxygen with FiO2 <0.5	Continuous positive airway pressure (CPAP) or nasal cannula with FiO2>0.5	Conventional ventilation	Alternative ventilation (e.g., HFOV)
Retinopathy of Prematurity (ROP)	Normal vascularizati on or not assessed	Incomplete vascularization	Pre-threshold ROP	Threshold ROP or ROP treatment	Retinal detachment
Sepsis	No sepsis symptoms and signs	Septic evaluation, no treatment	R/O sepsis, antibiotics for ≤72 hours	A confirmed blood stream infection with a course of antibiotic treatment, no septic shock or meningitis	A confirmed blood stream infection with a course of antibiotic treatment with septic shock or meningitis
Thrombocytopenia	>100 000	75,000-100,000	50,000< 75,000	25,000<50,000	< 25,000 or platelet transfusion

REFERENCES

1. Seale, A. C. *et al.* Neonatal severe bacterial infection impairment estimates in South Asia, sub-Saharan Africa, and Latin America for 2010. *Pediatr. Res.* **74**, 73–85 (2013).
2. Seale, A. C. *et al.* Estimates of possible severe bacterial infection in neonates in sub-Saharan Africa, south Asia, and Latin America for 2012: a systematic review and meta-analysis. *Lancet Infect. Dis.* **14**, 731–741 (2014).
3. Seale, A. C. *et al.* Estimates of possible severe bacterial infection in neonates in sub-Saharan Africa, south Asia, and Latin America for 2012: a systematic review and meta-analysis. *Lancet Infect. Dis.* **14**, 731–741 (2014).
4. Zaidi, A. K. *et al.* Hospital-acquired neonatal infections in developing countries. *Lancet* **365**, 1175–1188 (2005).
5. Campbell, O. M. R. *et al.* The scale, scope, coverage, and capability of childbirth care. *Lancet (London, England)* **388**, 2193–2208 (2016).
6. Investigators of the Delhi Neonatal Infection Study (DeNIS) collaboration. Characterisation and antimicrobial resistance of sepsis pathogens in neonates born in tertiary care centres in Delhi, India: a cohort study. *Lancet Glob. Heal.* **4**, e752–e760 (2016).
7. Chaurasia, S. *et al.* Neonatal sepsis in South Asia: huge burden and spiralling antimicrobial resistance. *BMJ* **364**, k5314 (2019).
8. Laxminarayan, R. *et al.* Access to effective antimicrobials: a worldwide challenge. *Lancet* **387**, 168–175 (2016).
9. Hamer, D. H. *et al.* Etiology of Bacteremia in Young Infants in Six Countries. *Pediatr. Infect. Dis. J.* **34**, e1–e8 (2015).
10. Huynh, B.-T. *et al.* Burden of bacterial resistance among neonatal infections in low income countries: how convincing is the epidemiological evidence? *BMC Infect. Dis.* **15**, 127 (2015).
11. Cassini, A. *et al.* Attributable deaths and disability-adjusted life-years caused by infections with antibiotic-resistant bacteria in the EU and the European Economic Area in 2015: a population-level modelling analysis. *Lancet. Infect. Dis.* **19**, 56–66 (2019).
12. UN. *Global Strategy for Women's, Children's, and Adolescents' Health (2016-2030)*. (2015).
13. Johnson, J. *et al.* Trends in Chlorhexidine Use in US Neonatal Intensive Care Units: Results From a Follow-Up National Survey. *Infect. Control Hosp. Epidemiol.* **37**, 1116–8 (2016).
14. Sinha, A., Sazawal, S., Pradhan, A., Ramji, S. & Opiyo, N. Chlorhexidine skin or cord care for prevention of mortality and infections in neonates. *Cochrane Database of Systematic Reviews* (2015). doi:10.1002/14651858.CD007835.pub2
15. Arifeen, S. El *et al.* The effect of cord cleansing with chlorhexidine on neonatal mortality in rural Bangladesh: A community-based, cluster-randomised trial. *Lancet* (2012). doi:10.1016/S0140-6736(11)61848-5
16. Semrau, K. E. A. *et al.* Effectiveness of 4% chlorhexidine umbilical cord care on neonatal mortality in Southern Province, Zambia (ZamCAT): a cluster-randomised controlled trial. *Lancet Glob. Heal.* **4**, e827–e836 (2016).
17. Darmstadt, G. L. *et al.* Safety and effect of chlorhexidine skin cleansing on skin flora of neonates in Bangladesh. *Pediatr. Infect. Dis. J.* **26**, 492–5 (2007).
18. Johnson, J. *et al.* The impact of chlorhexidine gluconate bathing on skin bacterial burden of neonates admitted to the Neonatal Intensive Care Unit. *J. Perinatol.* **39**, 63–71 (2019).
19. Sankar, M. J., Chandrasekaran, A., Ravindranath, A., Agarwal, R. & Paul, V. K. Umbilical cord cleansing with chlorhexidine in neonates: A systematic review. *Journal of Perinatology* (2016). doi:10.1038/jp.2016.28
20. Sankar, M. J. *et al.* Does skin cleansing with chlorhexidine affect skin condition, temperature and colonization in hospitalized preterm low birth weight infants?: a randomized clinical trial. *J. Perinatol.* **29**, 795 (2009).
21. Tielsch, J. M. *et al.* Impact of Newborn Skin-Cleansing With Chlorhexidine on Neonatal

Mortality in Southern Nepal: A Community-Based, Cluster-Randomized Trial. *Pediatrics* **119**, e330–e340 (2007).
22. Saleem, S. *et al.* Chlorhexidine vaginal and infant wipes to reduce perinatal mortality and morbidity: a randomized controlled trial. *Obstet. Gynecol.* **115**, 1225–32 (2010).
23. Sankar, M. J. & Paul, V. K. Efficacy and safety of whole body skin cleansing with chlorhexidine in neonates--a systemic review. *Pediatr. Infect. Dis. J.* **32**, e227-34 (2013).
24. Cutland, C. L. *et al.* Chlorhexidine maternal-vaginal and neonate body wipes in sepsis and vertical transmission of pathogenic bacteria in South Africa: a randomised, controlled trial. *Lancet (London, England)* **374**, 1909–16 (2009).
25. Cleminson, J. & McGuire, W. Topical emollient for preventing infection in preterm infants. *Cochrane Database Syst. Rev.* CD001150 (2016). doi:10.1002/14651858.CD001150.pub3
26. Darmstadt, G. L. *et al.* Topically applied sunflower seed oil prevents invasive bacterial infections in preterm infants in Egypt: a randomized, controlled clinical trial. *Pediatr. Infect. Dis. J.* **23**, 719–25 (2004).
27. Chapman, A. K., Aucott, S. W. & Milstone, A. M. Safety of chlorhexidine gluconate used for skin antisepsis in the preterm infant. *J. Perinatol.* **32**, 4–9 (2012).
28. Lashkari, H. P., Chow, P. & Godambe, S. Aqueous 2% chlorhexidine-induced chemical burns in an extremely premature infant: Figure 1. *Arch. Dis. Child. - Fetal Neonatal Ed.* **97**, F64–F64 (2012).
29. Sathiyamurthy, S., Banerjee, J. & Godambe, S. V. Antiseptic use in the neonatal intensive care unit - a dilemma in clinical practice: An evidence based review. *World J. Clin. Pediatr.* **5**, 159–71 (2016).
30. Andersen, C., Hart, J., Vemgal, P. & Harrison, C. Prospective evaluation of a multi-factorial prevention strategy on the impact of nosocomial infection in very-low-birthweight infants. *J. Hosp. Infect.* **61**, 162–7 (2005).
31. Lund, C. H. & Osborne, J. W. Validity and reliability of the Neonatal Skin Condition score. *JOGNN - J. Obstet. Gynecol. Neonatal Nurs.* **33**, 320–327 (2004).
32. Mullany, L. C. *et al.* A randomized controlled trial of the impact of chlorhexidine skin cleansing on bacterial colonization of hospital-born infants in Nepal. *Pediatr. Infect. Dis. J.* **27**, 505–11 (2008).
33. Mwananyanda, L. *et al.* Preventing Bloodstream Infections and Death in Zambian Neonates: Impact of a Low-cost Infection Control Bundle. *Clin. Infect. Dis.* (2018). doi:10.1093/cid/ciy1114
34. WHO | Chlorhexidine 7,1% digluconate (CHX) aqueous solution or gel (10ml): Reports of serious eye injury due to errors in administration. *WHO* (2019).
35. Pereira, L. *et al.* Randomized study of vaginal and neonatal cleansing with 1% chlorhexidine. *Int. J. Gynecol. Obstet.* **112**, 234–238 (2011).
36. Association of Anaesthetists of Great Britain and Ireland, J. P. *et al.* Safety guideline: skin antisepsis for central neuraxial blockade. *Anaesthesia* **69**, 1279–86 (2014).
37. MHRA. Chlorhexidine solutions: risk of chemical burn injury to skin in premature infants : MHRA. Available at: https://webarchive.nationalarchives.gov.uk/+/http://www.mhra.gov.uk/Safetyinformation/DrugSafetyUpdate/CON428307. (Accessed: 2nd September 2019)
38. IMPAACT P1106: Pharmacokinetic Characteristics of Antiretrovirals and Tuberculosis Medicines in Low Birth Weight Infants - Full Text View - ClinicalTrials.gov. Available at: https://clinicaltrials.gov/ct2/show/NCT02383849. (Accessed: 9th January 2020)
39. Hernán, M. A. & Robins, J. M. Per-Protocol Analyses of Pragmatic Trials. *N. Engl. J. Med.* **377**, 1391–1398 (2017).
40. Harwood-Nuss A, Wolfson A, L. C. *The clinical practice of emergency medicine*. (1996).
